# Synergistic anticancer activity of frankincense aqueous extract with sorafenib in HepG2 cells and its UHPLC–QTOF–MS/MS-based metabolomic profiling

**DOI:** 10.1038/s41598-026-42328-y

**Published:** 2026-03-27

**Authors:** Wesam Ragab, Kamel Mahmoud, Seham Salah El-Din El-Hawary, Osama M. Gomaa, Rasha M. Allam, Abeer S. Moawad, Rabab Mohammed

**Affiliations:** 1 Pharmacognosy Department, Faculty of Pharmacy, MUST University, 6th October City, Giza, 12566 Egypt; 2 Pharmacognosy Department, Faculty of Pharmacy, Cairo University, Cairo, 11562 Egypt; 3 Pharmacology Department, Medical and Clinical Research Institute, National Research Centre, 33 El-Bohouth St., Dokki, P.O.12622, Cairo, Egypt; 4 Pharmacognosy Department, Faculty of Pharmacy, Beni-Suef University, Beni-Suef, 62514 Egypt

**Keywords:** Frankincense, *Boswellia*, Sorafenib, *UHPLC-QTOF-MS/MS*, HepG2, Synergistic cytotoxicity, CompuSyn, Cancer, Drug discovery, Oncology

## Abstract

Liver cancer remains a major global health challenge, with limited therapeutic options for advanced stages. Sorafenib, the standard first-line systemic therapy, provides only modest survival benefits and is frequently associated with drug resistance and adverse effects, necessitating the exploration of safer and more effective combination therapies. Frankincense, the olibanum gum resin from *Boswellia sacra* Flück.(Burseraceae), has long been used as a traditional medicinal remedy and is known to target multiple biological pathways, with diverse therapeutic activities. Owing to its reported multi-target anticancer, anti-inflammatory, and pro-apoptotic properties, frankincense represents a promising candidate for combination therapy aimed at enhancing sorafenib efficacy while potentially reducing required doses and associated toxicity. This study aimed to evaluate the cytotoxic activity of frankincense aqueous extract (FrAE) alone and in combination with sorafenib (SOR) against the hepatoblastoma cell line HepG2. Phytochemical analysis tentatively identified five diterpenoids and sixteen triterpenoids in FrAE. Both FrAE and SOR exhibited dose-dependent cytotoxicity, and their combination selectively enhanced cytotoxic effects, reducing the effective concentration of SOR and demonstrating strong synergism (CI = 0.298). Mechanistically, the combination induced integrated anticancer effects characterized by enhanced apoptosis and necrosis, activation of autophagic signaling, and marked inhibition of HepG2 cell migration, accompanied by cell cycle disruption across multiple phases. Molecular docking further supported these findings by demonstrating favorable binding of FrAE-derived terpenoids to key apoptotic (BCL-2, p53) and autophagic (mTOR, LC3C) regulators. Collectively, these findings suggest that the aqueous extract of frankincense enhances the anticancer efficacy of sorafenib, representing a promising adjuvant strategy for the management of liver cancer.

**Supplementary Information:**

The online version contains supplementary material available at 10.1038/s41598-026-42328-y.

## Introduction

**P**rimary liver cancer ranks as the seventh most prevalent cancer and the second leading cause of cancer-related deaths^1^. It is mainly represented by hepatocellular carcinoma (HCC), one of the most lethal malignancies globally^2^. Over the past four decades, the incidence of HCC has increased significantly, with the corresponding mortality rate continually rising^3^. Although advances in therapy have improved overall survival rates, high recurrence and metastasis remain the leading causes of death among patients with HCC^4^. Furthermore, the majority of HCC cases are diagnosed in advanced stages, where standard chemotherapy shows limited efficacy, emphasizing the crucial need for more effective therapeutic strategies^5^.

**C**urrently, sorafenib remains the only approved targeted therapy for advanced liver cancer^6^. It is a multikinase inhibitor that has become one of the standard chemotherapeutic agents for the treatment of advanced HCC, blocking a range of kinases involved in the regulation of tumor growth, proliferation, and angiogenesis^7^. Multidrug therapy is a common practice in oncology, as it can achieve better therapeutic outcomes than monotherapy while minimizing adverse effects. As a result, researchers have focused on the use of herbal extracts or active natural products in combination with sorafenib to enhance its chemotherapeutic efficacy^8^.

**N**atural products have been used as lead compounds for anticancer agents for several decades, and more than half of the chemotherapeutic drugs currently used in clinical settings are either natural products or their semi-synthetic derivatives^9^. Plant-derived natural products have long been integral to drug discovery, offering structural diversity, broad availability, and low toxicity. Their molecular complexity enables them to target multiple cancer pathways, and recent research into combining these compounds with targeted therapies has demonstrated anticancer efficacy^10^. Frankincense is the olibanum gum resin obtained from the shrubs of the *Boswellia* species of the Burseraceae family. Among *Boswellia* species, *B. sacra* Flück. (syn. *B.* carteri Birdw.) is a species native to the Arabian Peninsula (Oman, Yemen) and Somalia. To obtain these resins, individual trees are usually incised during six months of the dry season^11^. Frankincense and its chemical components have anti-inflammatory, anticancer, antibacterial, anti-diabetic, neuroprotective, analgesic, anti-thrombotic, hepatoprotective, and cardioprotective actions^12–15^. Frankincense is primarily composed of diterpenoids and triterpenoids, with triterpenoids being the most abundant metabolites^16^. It has been reported that pentacyclic triterpenoids, especially boswellic acids, are the primary constituents responsible for its activity^17^. These compounds have demonstrated potential cytotoxic effects against various cancers, including prostate, cervical, breast, colorectal, pancreatic, and bladder cancers^18^. In this study, we used the HepG2 cell line, originally derived from a human hepatoblastoma and widely employed as an in vitro model in liver cancer research, including studies of HCC. It retains many hepatocyte-like features and relevant signaling pathways, making it suitable for mechanistic and cytotoxicity screening^19,20^. Using this model, the current study aimed to investigate the potential chemomodulatory effects of frankincense aqueous extract (FrAE) on the cytotoxic profile of sorafenib in liver cancer cells.

## Materials and methods

### Water decoction of frankincense

Frankincense (the dried oleo-gum-resin of *Boswellia sacra* Flück.) was obtained from plants grown in Oman and authenticated by Dr. Ibrahim Elgarf, Professor of Taxonomy at Cairo University, Egypt. A voucher specimen (No. ID- BUPD-133) was deposited in the herbarium of Phamacognosy department, Faculty of Pharmacy, Beni-Suef University, Egypt. The FrAE was prepared by boiling 50 g of dried frankincense powder in 500 ml of distilled water (1:10 *w/v*) for two cycles. The resulting supernatants were filtered using a Buchner funnel with vacuum filtration. The collected filtrates were evaporated using a rotary evaporator at 55 °C and then lyophilized^21^ to yield 27.3 g of a yellowish-white residue.

### UHPLC-QTOF-MS/MS Profiling of frankincense extract

Qualitative profiling of FrAE was performed using an Agilent 1290 Infinity II UHPLC system coupled to an Agilent 6545 ESI-Q-TOF–MS operated in both positive and negative ionization modes. A 1 µL aliquot of the extract (1 mg/ml in methanol) was injected onto a Kinetex phenyl-hexyl column (1.7 µm, 2.1 × 50 mm). The column and autosampler temperatures were maintained at 45 °C and 10 °C, respectively. The mobile phase consisted of solvent A (H₂O with 0.1% formic acid) and solvent B (95% acetonitrile, 5% H₂O with 0.1% formic acid), with an initial 1-min isocratic elution at 90% A followed by a 9-min linear gradient to 100% B at a flow rate of 0.4 mL/min. Electrospray ionization (ESI) conditions were as follows: capillary temperature, 320 °C; source voltage, 3.5 kV; and sheath gas flow, 11 L/min. Mass spectra were acquired at 6 scans/s with a minimum ion intensity threshold of 1000 counts, an isolation width of ~ 1.3 m/z, and a maximum of 9 precursors per cycle. Ramped collision energy was applied using the formula: 5 × (m/z)/100 + 10 eV. Internal lock masses used for calibration included purine [M + H]⁺ (m/z 121.050873) and hexakis (1H,1H,3H-tetrafluoropropoxy) phosphazene [M + H]⁺ (m/z 922.009798) in positive mode, and trifluoroacetic acid (TFA) [M – H]⁻ (m/z 112.985587) and phosphazene + TFA [M + TFA – H]⁻ (m/z 1033.988109) in negative mode. Data were converted from Agilent MassHunter (.d) format to mzXML using MSConvert software^22^.

### Quantitative analysis using HPLC

11-keto-β-boswellic acid (KBA), obtained from Nawah Scientific Inc. (Mokatam, Cairo, Egypt), and FrAE were analyzed using a Waters 2690 Alliance HPLC system equipped with a Waters 996 photodiode array detector and an Inertsil ODS Column (4.6 × 150 mm, 5 μm).

Quantification of KBA in FrAE was performed according to a modified method developed by Tawab, et al.^23^. A mixture of 0.1% orthophosphoric acid and acetonitrile was used at a flow rate of 1 ml/min; detection was performed at 250 nm, and the injection volume was 10 μL. A serial dilution of the standard stock solution was completed, and a calibration curve was established. To determine linearity, six concentrations of KBA, ranging from 10 to 60 μg/ml, were prepared from the stock solution.

### Sample preparation for biological assays

FrAE and SOR were dissolved in DMSO (Sigma-Aldrich) to prepare stock solutions of 50 mg/ml and 50 mM, respectively. This approach ensured that the final DMSO concentration in the biological assays remained within a subcytotoxic range (0.1%-0.5%).

### Cell culture

HepG2, a hepatoblastoma cancer cell line, and BNL CL.2, a mouse normal hepatocytes cell line, were obtained from Nawah Scientific Inc. Cells were maintained in DMEM media supplemented with 100 µg/ml of streptomycin, 100 units/ml of penicillin, and 10% of heat-inactivated fetal bovine serum in a humidified, 5% (*v/v*) CO_2_ atmosphere at 37 °C^24^.

### *Sulforhodamine B (SRB) *In vitro* cell viability assay*

Cell viability was evaluated using the sulforhodamine B (SRB) assay. HepG2 cells and BNL CL.2 cells (5 × 10^3^ cells/well) were seeded in 96-well plates in 100 μL of complete medium and incubated for 24 h. HepG2 cells were exposed to FrAE and the standard chemotherapy, SOR, at various concentrations (1, 3, 10, 30, and 100 µg/ml and 1, 3, 10, 30, and 100 µM, respectively). In contrast, BNL CL.2 cells were exposed to FrAE and the standard chemotherapy, SOR, at various concentrations (1, 10, 30, 100, and 300 µg/ml and 1, 10, 30, 100, and 300 µM, respectively). Equitoxic concentrations of FrAE and SOR were combined. After treatment for 72 h, cells were fixed with 150 µL of 10% trichloroacetic acid (TCA; Merck) at 4 °C for 1 h. After washing five times with distilled water, 70 µL of 0.4% (w/v) SRB solution (Sigma-Aldrich) was added, and plates were incubated for 10 min at room temperature in the dark. Excess dye was removed by washing the plates three times with 1% acetic acid (Chem-Lab), and they were allowed to air dry. Subsequently, 150 µL of 10 mM Tris buffer (pH 10.5; Chem-Lab) was added to solubilize the bound dye. Absorbance was measured at 540 nm using a BMG LABTECH® FLUOstar Omega microplate reader (Ortenberg, Germany)^25^.

### Evaluation of the selectivity index (SI)

The selectivity of FrAE, SOR, and their combination toward cancer cells was assessed using the selectivity index (SI). This parameter reflects a compound’s preferential toxicity toward cancer cells relative to normal cells.

$$SI\, = \,IC_{50} for \, normal \, cell \, line \, / \, IC_{50} for \, cancer \, cell \, line$$

Compounds with an SI value greater than 2 are considered selectively toxic to cancer cells, whereas an SI below 2 indicates low selectivity, suggesting potential cytotoxic effects on normal cells as well^26^.

### Assessment of morphological changes

Morphological changes in HepG2 and BNL CL.2 cells after treatment with FrAE and SOR were analyzed microscopically. HepG2 and BNL CL.2 cells were seeded into 6-well plates and treated with FrAE and SOR at different concentrations for 72 h. Cells were imaged at 100 × magnification using an inverted phase-contrast microscope (PrimoVert, Carl Zeiss).

### Evaluation of synergism

The effects of the combination were analyzed with CompuSyn software. The CompuSyn software calculates the combination index (CI) using the median-effect principle. This software is based on Chou and Talalay’s multiple drug effect Equations^27^.

### Flow cytometric analysis

#### Cell cycle analysis

 HepG2 cells were treated with the pre-calculated IC_50_ of FrAE and SOR, alone or in combination, for 48 h. Then, cells were harvested by trypsinization, washed twice with phosphate-buffered saline (PBS), fixed in ice-cold 60% ethanol at 4 ºC, and re-washed in PBS. After that, the cells are resuspended in 500 μL of propidium iodide (PI) with RNase staining buffer (BD, Franklin Lakes, NJ, USA) and incubated for 30 min. Lastly, FACS analyses were performed using the ACEA Novocyte™ flow cytometer (ACEA Biosciences Inc., San Diego, USA). For every sample, data from 12,000 cells were collected, and the distribution of cell cycle phases was analyzed using ACEA Novo Express™ software (ACEA Biosciences Inc.)^28^.

#### Apoptosis detection (annexin V-FITC/PI assay)

 HepG2 cells were treated for 48 h with the pre-calculated IC_50_ of FrAE and SOR, alone or in combination. Then, they were trypsinized and washed twice with PBS. Apoptosis was assessed using the Annexin V-FITC/PI Apoptosis Detection Kit (BD Biosciences, San Diego, USA) according to the manufacturer’s instructions. Briefly, cells were resuspended in 0.5 mL of the binding buffer. Then, the staining solution (5 μL of Annexin V-FITC and 5 μL of PI) was added and incubated for 15 min at room temperature in the dark. Finally, the cells were subjected to FACS analysis using an ACEA Novocyte™ flow cytometer (ACEA Biosciences Inc.)^29^.

#### Autophagy detection (acridine orange staining)

 For autophagic assessment in response to FrAE and SOR alone or combined for 48 h, HepG2 cells were trypsinized and washed twice with ice-cold PBS. Then, 0.5 mL of the staining solution (1 μg/ml acridine orange in PBS) was added and incubated in the dark at room temperature for 30 min. Cells were adjusted to analyze 12,000 events by flow cytometric analysis using an ACEA Novocyte™ flow cytometer, and fluorescent signals were analyzed with the FL1 signal detector (488 nm excitation/530 nm emission). The net fluorescent intensities (NFI) were quantified^30^.

### Cell migratory potential by cell scratch assay

The effect of combining FrAE with SOR on the migration of HepG2 cells was assessed using an in vitro scratch assay. HepG2 cells were seeded at 90% confluence in 12-well plates in triplicate for each condition. After 24 h, a scratch was introduced across the center of each well using a 1 mm pipette tip. Cells were washed twice with PBS to remove cell debris and then incubated with fresh media alone or containing FrAE and/or SOR. Cells were allowed to migrate into the wound surface, and the average distance of migrating cells was determined by inverted microscopy at designated time points (0, 24, 48, 72, and 96 h)^31^**.**

$${\mathbf{Wound}} \, {\mathbf{closure}}\,\left( \% \right){\text{was calculated as}}:\% {\text{ closure}}\, = \,\left[ {\left( {{\mathrm{At}}\, = \,0 {\mathrm{h}}\, - \,{\mathrm{At}}\, = \,\Delta {\mathrm{h}}} \right) \, /{\text{ At}}\, = \,0 {\mathrm{h}}} \right]\, \times \,{1}00,$$

where At = 0 h is the wound area at time zero, and At = Δh is the area after h hours.

### Quantitative real-time PCR (qRT-PCR)

Total RNA was extracted from the samples using the TransZol Up Plus RNA kit (Code #ER501-01; Trans, China) according to the manufacturer’s instructions. RNA concentration and purity were assessed using a FLUOstar Omega plate reader spectrophotometer (BMG LABTECH) by measuring the A260/280 ratio. The isolated RNA was immediately reverse-transcribed into complementary DNA (cDNA) using the EasyScript® First-Strand cDNA Synthesis SuperMix (Cat. No. AE301; TransGene, China), according to the manufacturer’s instructions. Quantitative real-time PCR (qPCR) was performed on a Bio-Rad CFX Opus 96 system using Xpert Fast SYBR® (uni) master mix (Cat. No. GE20.0100; Porto, Portugal). The reaction volume was 20 µL, including 2 µL of cDNA, 0.2 µM each of forward and reverse primers, 10 µL of SYBR Green master mix, and 6 µL of nuclease-free water. The primer sequences used in this study were designed using Primer3 software and synthesized by Vivantis Technologies (Selangor, Malaysia). Relative gene expression levels were calculated using the 2 − ΔΔCT method^32^. The primer sequences for human GAPDH, LC3B (MARCH 1), and P21 are listed below:

- **GAPDH sequences**
- F: 5’-GTC TCC TCT GAC TTC AAC AGC G-3’
- R: 5’-ACC ACC CTG TTG CTG TAG CCA A-3’
- **LC3B sequences**
- F: 5’-GAG AAG CAG CTT CCT GTT CTG G-3’
- R: 5’-GTG TCC GTT CAC CAA CAG GAA G-3’
- **P21 sequences**
- F: 5’-AGGTGGACCTGGAGACTCTCAG-3’
- R: 5’-TCCTCTTGGAGAAGATCAGCCG-3’

### Data analysis

Dose–response curves were analyzed using the E_**max**_ model, and IC_50_ (half-maximal inhibitory concentration) was calculated as previously described^33^. All analyses were performed using GraphPad Prism version 10.1.0 (GraphPad Software Inc., San Diego, CA). All data were expressed as mean ± SD, with statistical significance indicated when *P* ≤ 0.05. Statistical comparisons between control and treated groups were determined using one-way ANOVA with the Tukey–Kramer post hoc test.

### Molecular docking

Docking simulations were performed to determine binding affinities and to examine interactions between the compounds from FrAE and the active sites of two apoptosis-related proteins (BCL-2 and p53) and two autophagy-related proteins (mTOR and LC3C). The crystal structures of the proteins (PDB IDs: 8HOI, 3ZME, 9F44, and 3WAM, respectively) were downloaded from the Protein Data Bank (PDB). Docking was performed using PyRX 0.8 software with the AutoDock Vina plugin. Protein preparation involved removing water molecules, co-crystallized solvents, ligands, and other heteroatoms. All ligands were energy-minimized and converted to PDBQT format using the Open Babel tool. Validation of the docking simulation was achieved through redocking of the co-crystallized ligands. Docked conformations yielded RMSD values ranging from 0.724 to 2.690 Å, as calculated by the DockRMSD server^34^, which validated our method. The alignment of co-crystallized ligands with the docked ligand poses can be found in the supplementary material (Suppl. Table 4**)**. Analysis and visualization of 2D and 3D images were performed using BIOVIA Discovery Studio Visualizer (v25.1.0.24284). Grid box coordinates of the binding center and dimensions (Å) for different proteins were set as illustrated in Table 1, while exhaustiveness was set at 8.

**Table 1:** Grid box coordinates of binding center and grid dimensions in (Å) applied for AutoDock-based molecular docking against BCL-2 (PDB ID: 8HOI), p53 (PDB ID: 3ZME), LC3C (PDB ID: 3WAM), and mTOR (PDB ID: 9F44).

		BCL2_8hoi pdbqt	p53_3zme pdbqt	(LC3C)_3wam pdbqt	mtor_9f44 .pdbqt
Grid box center coordinates	Center_x	17.2113308783	92.0787285295	-39.7753141081	309.375172354
	Center_y	-2.69690013105	94.2030446944	9.79580838975	372.82450863
	Center_z	40.6986988075	-45.5318180995	0.152833063877	305.172037782
Grid box dimensions	Size_x	12.8325389022	15.1618690899	9.9706129295	12.6866488566
	Size_y	16.5744002484	20.6493786467	10.3049595834	8.71117493573
	Size_z	20.5163920487	16.5262232558	10.5122589244	14.4067364049

## Results and discussion

### UHPLC-QTOF-MS/MS Qualitative profiling of frankincense extract

**Fig. 1:**
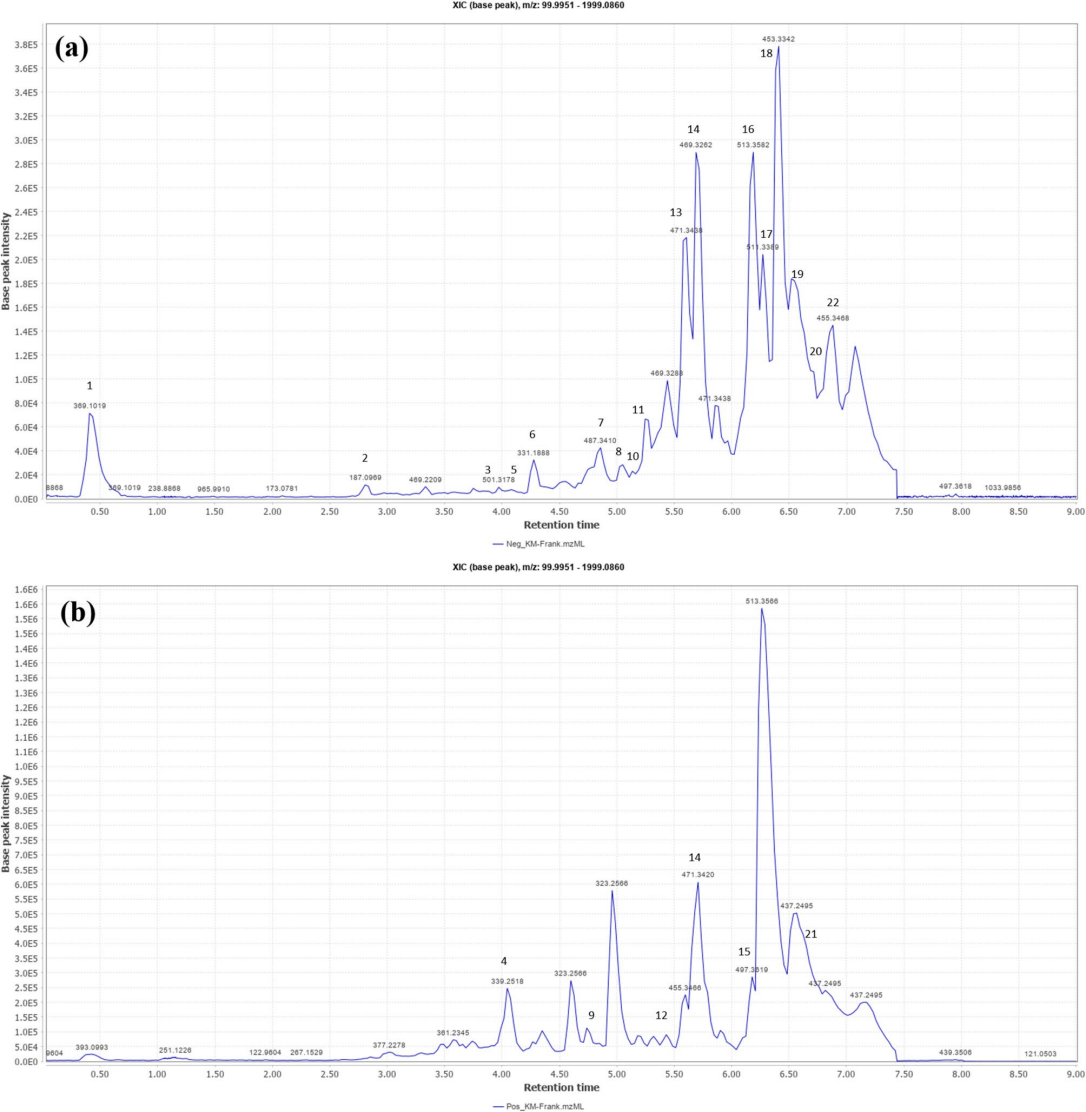
Total ion chromatogram (base peak) of frankincense aqueous extract (FrAE) in the **(a)** negative and **(b)** positive ionization modes.

The Base peak chromatograms of the FrAE obtained in negative and positive ionization modes revealed the annotation of 22 compounds belonging to various chemical classes. Specifically, two cembrane diterpenes and three prenyl aromadendrane diterpenes, two oleane-type triterpenes, four urasane-type triterpenes, three lupane-type triterpenes, and four tricullane-type triterpenes were tentatively identified (Table 2**, **Fig. 1). Compound identification was based on retention time, accurate mass, fragmentation pattern, and comparisons with MS/MS literature data^16,35,36^.

**Table 2:** *UHPLC–QTOF–MS/MS*–based tentative identification of phytochemicals in Frankincense aqueous extract (Oleo-gum resin of the *Boswellia sacra* Flück).

Comp No	Mol. Formula	Name	Class	m/z [M-H]¯	m/z [M + H] +	Rt	PPM	MS/MS fragmentation	Ref
1	C_13_H_21_O_12_	Methyl-hexosyl-uronic acid-hexoside	Glycan	369.1029		0.42	-2.57	309.0815, 249.0578, 207.0505, 189.0392, 113.0238	^37,38^
2	C_9_H_15_O_4_	Azelaic acid	Saturated dicarboxylic acid	187.0966		2.79	-5.25	169.0827, 143.1055, 125.0949, 97.0647	^39^
4	C_20_H_35_O_4_	Boscartin C	Cembrane (diterpene)		339.2537	4.03	2.1	321.2432, 293.2034	^35^
9	C_20_H_35_O_3_	Epoxy cembradiene-diol (1,4-Epoxy-8,13-cembrandien-5,12-diol)	Cembrane (diterpene)		323.2583	4.97	0.71	306.2545, 305.2465, 278.2542	^35^
6	C_20_H_27_O_4_	Boscartol L or K	Prenyl aroma dendrane-diterpene	331.1905		4.26	-2.97	313.1776, 287.1962	^35^
8	C_20_H_27_O_3_	Boscartol G	Prenyl aroma dendrane-diterpene	315.1954		4.87	-3.71	297.2359, 205.8933, 125.7870	^35^
12	C_20_H_33_O_2_	Boscartol C	Prenyl aroma dendrane-diterpene		305.2476	5.58	0.31	287.2345, 245.1874, 207.1703, 113.0935	^35^
3	C_30_H_45_O_6_	Unknown	Triterpene	501.3219		3.98	-0.52	483.3033, 469.2961, 457.3267, 371.2591	
5	C_30_H_47_O_7_	Unknown	Triterpene	519.3309		4.17	-3.52	501.3210, 487.3060	
7	C_30_H_47_O_5_	Trihydroxy-oleanenoic acid (3,6,7-Trihydroxyolean-12-en-27-oic-acid)	Oleane triterpene	487.3414		4.84	-3.07	469.3262, 443.3091, 383.2939, 373.2683	^16,35^
10	C_30_H_45_O_5_	Atricin B	Oleane triterpene	485.3263		5.34	-1.95	467.3130, 449.3078, 431.2886	^35^
21	C_30_H_49_O	α-Amyrenone	Oleane triterpene		425.3772	6.77	-1.39	407.3260, 287.1992, 269.2228	^16^
		β-Amyrenone	Ursane triterpene					407.3260, 329.2844, 323.2379	
13	C_30_H_47_O_4_	11-hydroxy-β-boswellic acid	Ursane triterpene	471.3466		5.59	-2.94	409.3428, 393.3143, 377.2828, 253.1558, 233.1912	^35,36^
14	C_30_H_45_O_4_	11-Keto-β-boswellic acid	Ursane triterpene	469.3314	471.3472	5.73	-1.99	451.3188, 407.3297, 391.2933, 339.2283, 321.2144, 271.1438	^35,36^
15	C_32_H_49_O_4_	3-O-Acetyl-9,11-dehydro-β-boswellic acid	Ursane triterpene		497.3629	6.16	0.73	437.3367, 419.3275, 299.1977	^35^
17	C_32_H_47_O_5_	3-O-Acetyl 11-keto-β-boswellic acid	Ursane triterpene	511.3416		6.28	-2.54	467.3505, 453.5042, 439.6259	^35,36^
20	C_33_H_51_O_5_	3-O-Acetyl-11-methoxy-β-boswellic acid	Ursane triterpene	527.3727		6.76	-2.84	513.0829, 493.9458, 486.9886, 462.9933, 455.3468, 443.2580, 423.3238	^16,35^
16	C_32_H_49_O_5_	3-O-Acetyl-27-hydroxy-lupeolic acid	Lupane triterpene	513.3572		6.20	-2.63	498.0741, 495.3432, 469.3638, 453.3342, 431.2742	^16,35^
20	C_33_H_51_O_5_	3-O-Acetyl-27-hydroxy-lupeolic acid methyl ester	Lupane triterpene	527.3727		6.76	-2.84	513.0829, 493.9458, 486.9886, 462.9933, 455.3468, 443.2580, 423.3238	^16,35^
21	C_30_H_49_O	Lupenone	Lupane triterpene		425.3772	6.77	-1.39	407.3260, 217.1951, 205.1212	^16,35^
11	C_32_H_47_O_6_	Boscartene A	Tricullane triterpene	527.3366		5.36	-2.3	509.3184, 467.1804, 370.2518	^35^
18	C_30_H_45_O_3_	3-oxo-tirucallic acid; β-Elemonic acid	Tricullane triterpene	453.3363		6.39	-2.47	371.2591, 353.2438, 339.2666, 269.1867	^35,36^
19	C_32_H_49_O_4_	3-O-Acetoxy-tirucallic acid	Tricullane triterpene	497.3623		6.72	-2.68	437.3363, 415.2847, 397.2692, 355.2626	^35,36^
22	C_30_H_47_O_3_	3-hydroxytirucallic acid; α-Elemolic acid	Tricullane triterpene	455.3518		6.86	-2.79	437.3363, 409.3428, 377.3166, 361.2876, 237.2960	^35,36^

### Identification of different types of compounds from frankincense extract

#### Identification of diterpenes

 Five compounds represented this chemical class (Compounds 4, 6, 8, 9, and 12), of which three compounds (6, 8, and 12) were tentatively identified as prenyl aromadendrane, while the remaining two were tentatively identified as cembranes.

#### Cembrane-type diterpenes

 Compounds 4 and 9 [m/z 339.2537 (C_20_H_35_O_4_) −] and [m/z 323.2583 (C_20_H_35_O_3_) −] were tentatively identified as Boscartin C (Fig. 4a) and Epoxy cembradiene-diol, respectively. The MS fragmentation of this class involves the loss of methyl groups and the elimination of water^35,40^.

#### Prenyl aromadendrane-type diterpenes

 Compounds 6, 8, and 12 [m/z 331.1905 (C_20_H_27_O_4_) +], [m/z 315.1954 (C_20_H_27_O_3_) +], and [m/z 305.2476 (C_20_H_33_O_2_) −] were tentatively identified as Boscartol L or K (Fig. 4b), Boscartol G, and Boscartol C, respectively. The MS fragmentation of this class includes the cleavage of a long-chain substituent from the main ring. Compound 6 showed peaks at 313 Da (M-H-H_2_O), 287 Da (M-H–CO), and 271 Da (M-H-CH_3_)^35,41,42^.

#### Identification of triterpenes

**Fig. 2:**
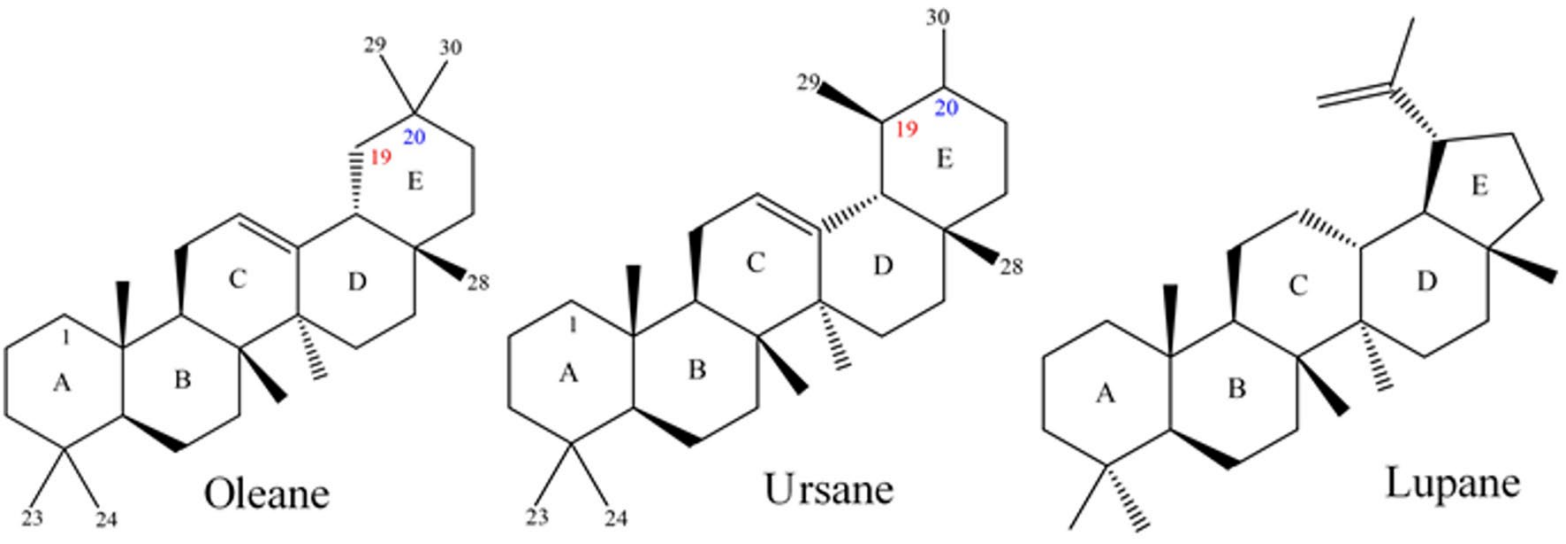
Representative skeletons of pentacyclic triterpenes: oleane, ursane, and lupane.

**Fig. 3:**
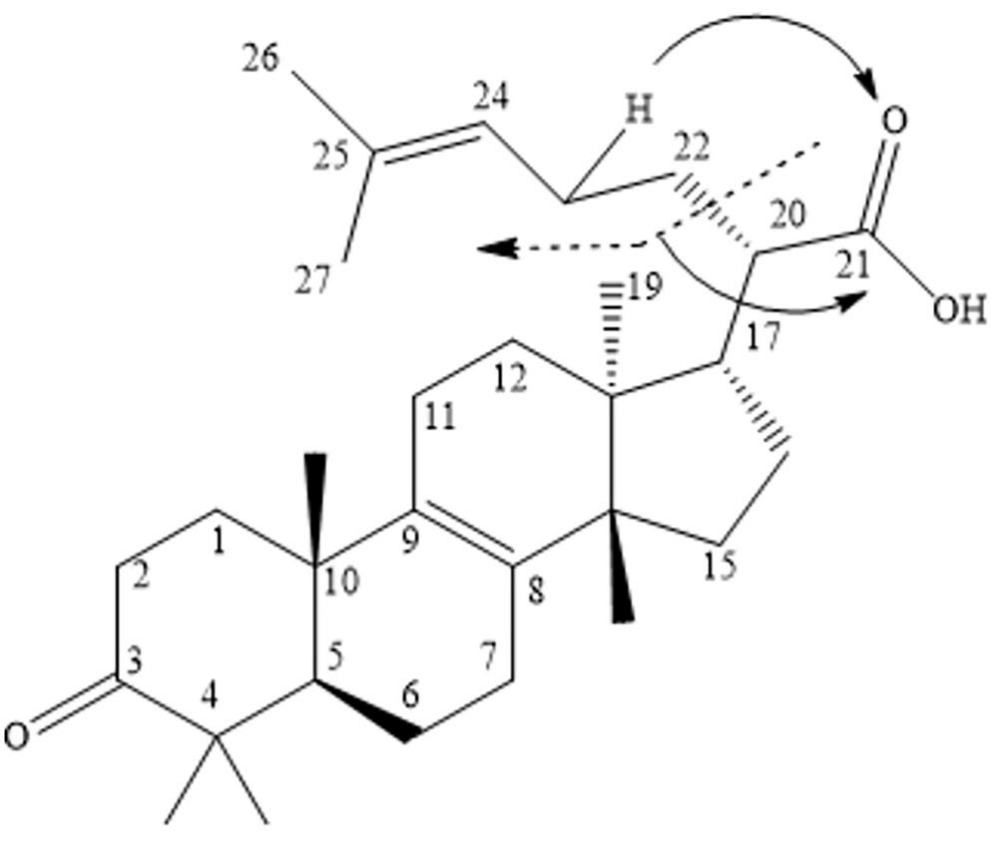
McLafferty rearrangement (MFR) of tricullane-type triterpenes.

Frankincense triterpenes are mostly oleanes, ursanes, lupanes, or tricullanes derivatives^18^. The fragmentation of triterpenes resulted in many characteristic peaks, primarily due to the loss of water, a methyl group, and carbon dioxide, or to a retro-Diels–Alder (RDA) reaction^43^. Among the 13 tentatively identified triterpenoid metabolites, five boswellic acids (BAs) were tentatively identified, each containing a penta-cyclic triterpene ring. BAs exist as ursane (β) and oleane (α) types, depending on the position of the two methyl groups at C-19/C-20, as shown in Fig. 2. However, (β-) and (α-) BAs could not be differentiated by MS spectra. Another class of triterpenoids, the tricullanes, follows the McLafferty rearrangement (MFR) at the C-21 acid, followed by breakage of the bond between C-20 and C-17^44^ as shown in Fig. 3.

#### Oleane-type triterpenes

**Fig. 4:**
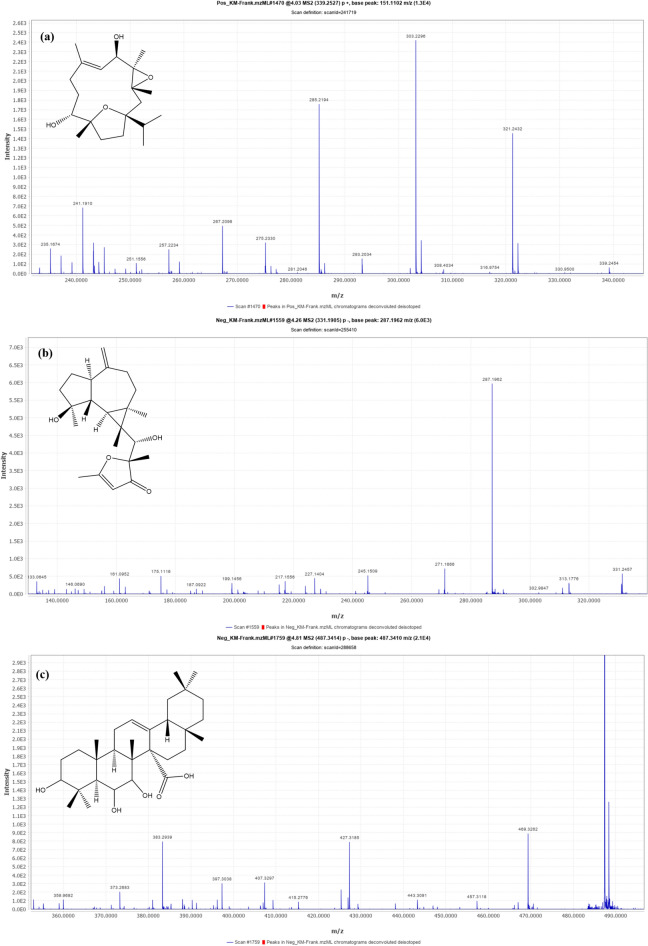
*UHPLC-QTOF-MS/MS* spectra of the main identified compounds in FrAE: **(a)** Boscartin C (Cembrane diterpene), **(b)** Boscartol L (Prenyl aroma dendrane diterpene), and **(c)** 3,6,7-Trihydroxyolean-12-en-27-oic acid (Oleane triterpene).

 Compounds 7 and 10 were tentatively identified as trihydroxy-oleanenoic acid and atricin B, respectively, based on mass data and by referring to the literature. **Compound 7** [m/z 487.3414 (C_20_H_35_O_3_) −] (Fig. 4c) underwent dehydration to give a peak at 469.3 Da (M-H-H_2_O), along with two additional daughter peaks at 443.3 Da (M-H-CO_2_) and 427.3 Da (M-H-CH_3_), respectively. RDA fragmentation produced characteristic fragments at 239.3 Da (containing rings A and B) and 247.3 Da (containing rings D and E)^35^.

#### Ursane-type triterpenes

**Fig. 5:**
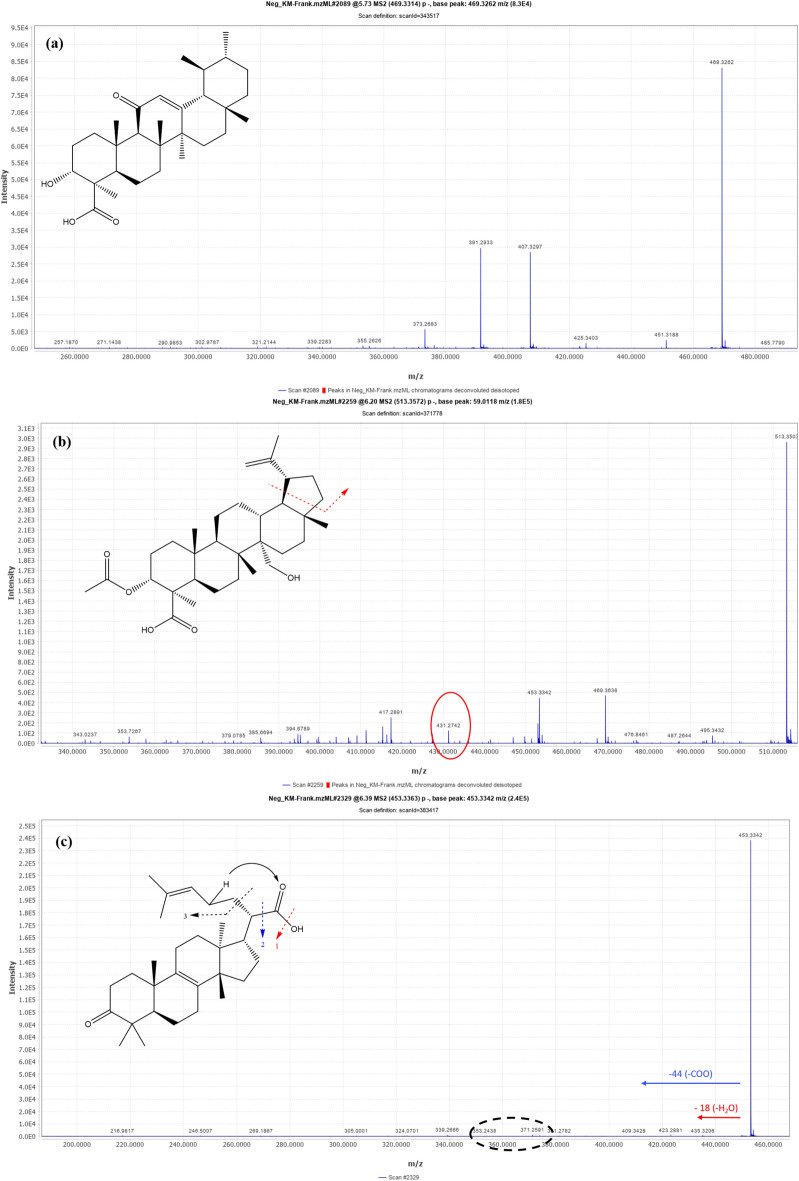
*UHPLC-QTOF-MS/MS* spectra of the main identified compounds in FrAE **(a)** 11-Keto-β-boswellic acid (Ursane triterpene), **(b)** 3-*O*-Acetyl-27-hydroxy-lupeolic acid (Lupane triterpene), and **(c)** 3-oxo-tirucallic acid (Tricullane triterpene).

 Based on fragmentation data and by referring to the literature, Compounds 13, 14, 15, and 17 were tentatively identified as 11-hydroxy-β-boswellic acid, 11-Keto-β-boswellic acid, 3-*O*-Acetyl-9,11-dehydro-β-boswellic acid, and 3-*O*-Acetyl-11-keto-β-boswellic acid, respectively. Compound 14 [m/z 469.3314 (C_30_H_45_O_4_) −] showed its quasi-molecular ion peak (M-H) at 469.3 Da along with its MS^2^ fragment at 451.73 Da (M-H-H_2_O) due to dehydration, which further underwent RDA rearrangement to give a characteristic peak at 231 Da, a fragment (containing rings D and E)^35,36^. Moreover, a characteristic daughter peak was detected at 407.3 Da, corresponding to the elimination of carbon dioxide and water (M-H–CO2–H2O). This pattern is characteristic of a structure with an 11-keto group; therefore, compound 14 was tentatively identified as 11-keto-β-boswellic acid (Fig. 5a).

#### Lupane-type triterpenes

 Lupanes were represented by three compounds (16, 20, and 21). Compound 16 [m/z 513.3572 (C_32_H_49_O_5_) −] was tentatively identified as 3-O-Acetyl-27-hydroxy-lupeolic acid (Fig. 5b). It showed its quasi-molecular ion peak (M-H) at 513.3 Da, along with an MS^2^ fragment at 495.3 Da (M-H-H_2_O) due to dehydration, together with a characteristic fragment peak at 431.2 Da resulting from the cleavage of ring E. Additional characteristic mass fragments were observed at 469.3 Da (M-H-CO_2_), corresponding to the loss of carbon dioxide, and at 453.3 Da (M-H-CH_3_COO), corresponding to the elimination of the acetyl group^16,35^.

#### Tricullane-type triterpenes

 Compounds 11, 18, 19, and 22 were the representatives of tricullanes. Their fragmentation follows McLafferty rearrangement (MFR). Compound 18 showed a quasi-molecular ion peak (M-H) at 453.3 Da. It showed characteristic MS^2^ fragments at 435 Da (M-H-H_2_O), and at 409 Da (M-H-CO_2_) due to loss of carbon dioxide, along with McLafferty rearrangement fragments at 371 Da and 353 Da. Based on mass data and reference to the literature, this compound was tentatively identified as 3-oxo-tirucallic acid^36^. Compound 19 exhibited a quasi-molecular ion peaks (M-H) at 497.3 Da and a fragment at 437 Da (M-H-CH3OO), along with daughter peaks resulting from the McLafferty rearrangement at 415.2 Da and 355.2 Da. According to the literature, it was tentatively identified as 3-*O*-acetoxy tirucallic acid^36^. Compound 22 showed a quasi-molecular ion peak (M-H) at 455 Da, along with a daughter peak at 437.3 Da (M-H-H_2_O), in addition to McLafferty rearrangement fragments at 377 Da and 361 Da. It was characterized as 3-hydroxytirucallic acid or α-Elemolic acid (Fig. 5c)^36^.

**Fig. 6:**
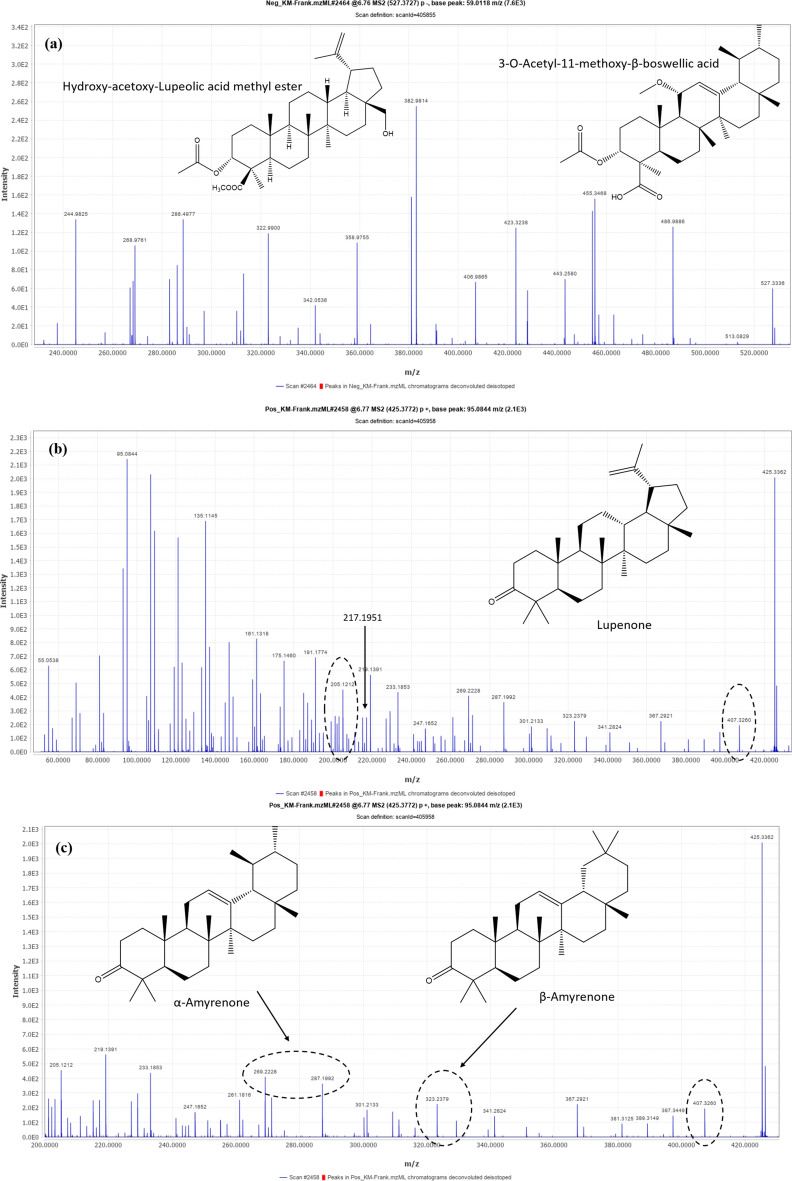
*UHPLC-QTOF-MS/MS* spectra of **(a)** Compound no.20 [m/z 527.3727 (C_33_H_51_O_5_) −], and **(b) & (c)** Compound no.21 [m/z 425.3772 (C_30_H_49_O) +].

Identification of **compound 20** [m/z 527.3727 (C_33_H_51_O_5_) −] was challenging, as the characteristic fragment resulting from the cleavage of ring E was not obesrved in the MS^2^ spectra. It could be tentatively identified as 3-*O*-Acetyl-11-methoxy-β-BA or 3-*O*-Acetyl-27-hydroxy-lupeolic acid methyl ester (Fig. 6a)^16,35^.

Identification of **compound 21** [m/z 425.3772 (C_30_H_49_O) +] was also challenging. Based on mass data and by reference to the literature, it was putatively identified as Lupenone (Fig. 6b) or α/β-Amyrenone (Fig. 6c)^16,35^. The MS^2^ spectrum is is highly congested, and the characteristic fragment peaks of Lupenone and α/β-Amyrenone were detected. The identification of these compounds is therefore regarded as putative rather than tentative.

*UHPLC-QTOF-MS/MS* spectra of the remaining compounds identified in FrAE are provided in the supplementary material file.

### Key terpenoids identified in FrAE and their reported cytotoxic pathways

Metabolomic profiling of FrAE revealed a diverse array of terpenoids with reported anticancer relevance, predominantly diterpenoids and triterpenoids.

#### Diterpenoids

 Several cembranoid diterpenes have been reported in *Boswellia* species. While some cembranoids showed limited or no direct cytotoxicity across tested human cancer cell lines, the cembrane scaffold is generally associated with modulation of cell signaling and oxidative stress pathways. Notably, prenylaromadendrane-type diterpenoids (boscartols) isolated from *B. sacra* resin exhibited marked cytotoxicity against U87-MG glioma cells at low micromolar concentrations, surpassing 5-fluorouracil. Related cembranoids and prenyl-aromadendranoids have also demonstrated anti-inflammatory, antioxidant, hepatoprotective, and apoptosis-modulating activities, suggesting indirect or context-dependent anticancer potential^45–49^.

### Triterpenoids

**Fig. 7:**
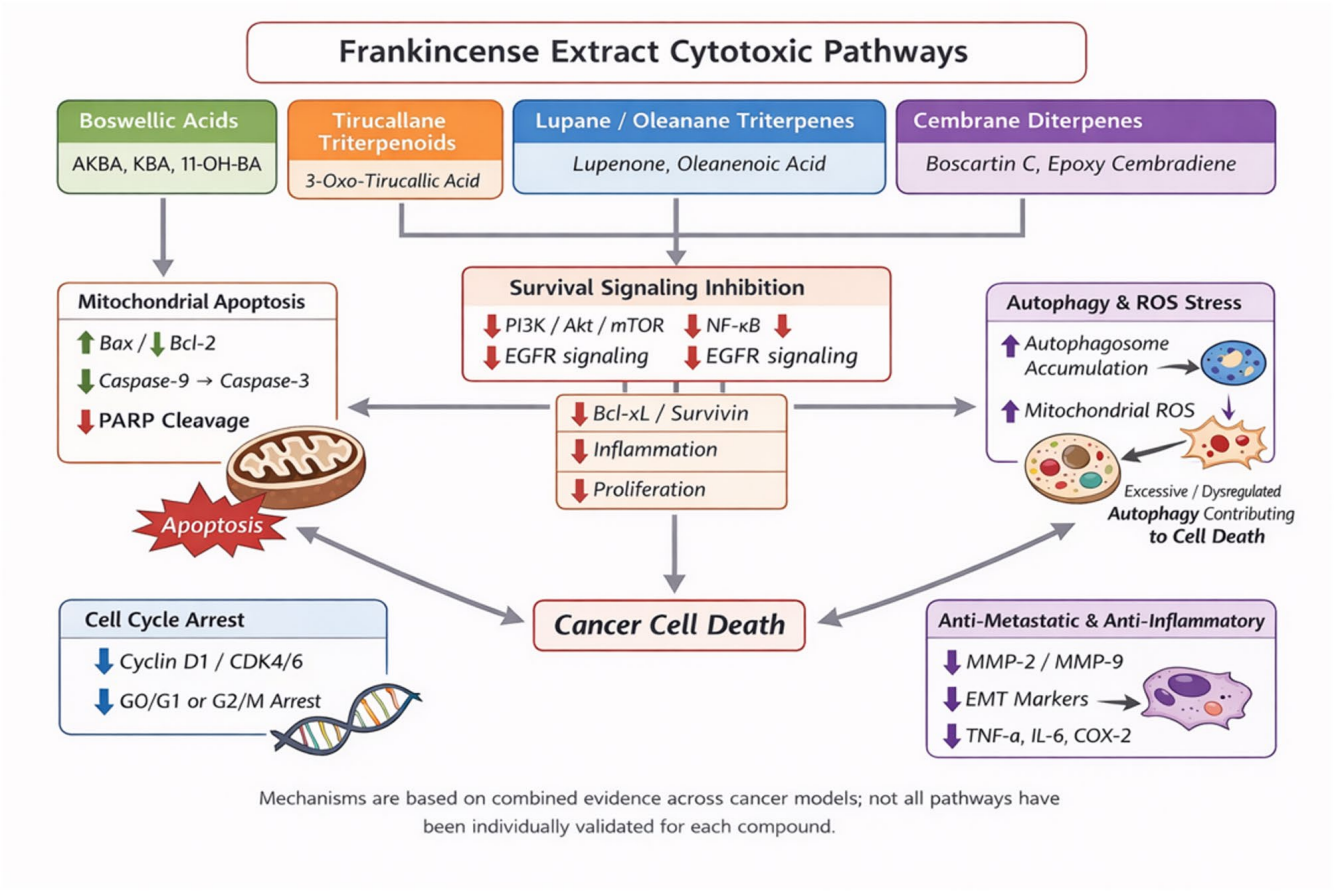
Schematic representation of proposed molecular mechanisms underlying the cytotoxic and anti-metastatic effects of frankincense-derived terpenoids in cancer cells. (This integrative mechanistic model is based on cumulative evidence from hepatocellular carcinoma and other cancer models; not all pathways have been individually validated for each identified compound).

**Fig. 8:**
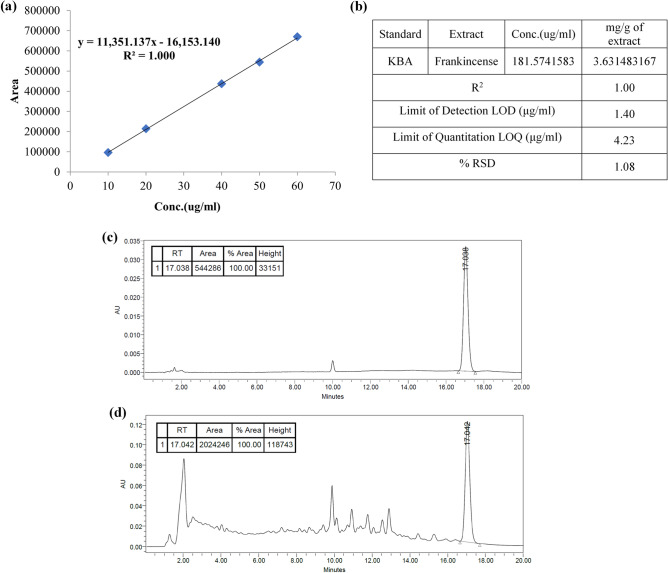
(**a**) The linearity of the KBA standard solution. (**b**) Measured concentration of KBA in FrAE, LOD, LOQ, and % RSD (**c**) and (**d**) HPLC chromatogram at 250 nm of a standard solution of 11-keto-β-boswellic acid and FrAE at retention times 17.038 and 17.042 min, respectively.

1. **Boswellic acid derivatives:** Pentacyclic triterpenoids of the boswellic acid family are well-established bioactives in *Boswellia* resins. These compounds exhibit dose-dependent cytotoxic and anti-proliferative effects across multiple cancer models, mediated by apoptosis induction, cell-cycle arrest, NF-κB suppression, and inhibition of PI3K/Akt and MAPK signaling pathways^11,48^.
2. **Lupane- and oleane-type triterpenoids:** Trihydroxy-oleanenoic acid, 3-O-acetyl-27-hydroxy-lupeolic acid, its methyl ester, and lupenone were detected. Although direct cytotoxicity data for these specific compounds from frankincense remain limited, structurally related triterpenoids have been widely reported to exert anti-proliferative, pro-apoptotic, and anti-metastatic effects in cancer models through mitochondrial dysfunction, modulation of reactive oxygen species, and inhibition of oncogenic transcription factors^50–52^. The presence of these triterpenoids suggests they may contribute additively or synergistically to the extract’s overall cytotoxic profile.
3. **Tirucallane triterpenoids:** Recent isolation of tirucallane triterpenoids from *B. sacra* resin has demonstrated dose-dependent inhibition of HepG2 and HCT-116 proliferation, with mechanistic evidence suggesting that these compounds upregulate the Bax/Bcl-2 ratio, (Fig. 7) promote caspase-3 activation and PARP cleavage, and decrease phosphorylated EGFR^52,53^.
4. **Quantitative analysis of frankincense extract by HPLC*.*** For quantification of KBA in the FrAE, a calibration curve was established, and the percentage of KBA in FrAE was determined. FrAE was found to contain 0.363% of KBA. The calculated LOD and LOQ were 1.40 μg/ml and 4.23 μg/ml, respectively. The results demonstrated a low relative standard deviation (RSD), confirming the precision of the method (Fig. 8).

### Frankincense selectively inhibits the growth and survival of HepG2 cells and synergizes the cytotoxicity of sorafenib

HCC is an aggressive disease and a leading cause of cancer deaths globally, presenting a significant medical challenge^54,55^. Natural products have shown promising potential in the prevention and treatment of HCC^56,57^. Recently, there has been a growing interest in exploring the anticancer potential of compounds derived from frankincense. In vitro studies have shown that frankincense exhibits cytotoxic effects against various human tumor cell lines^58–60^.

**Fig. 9:**
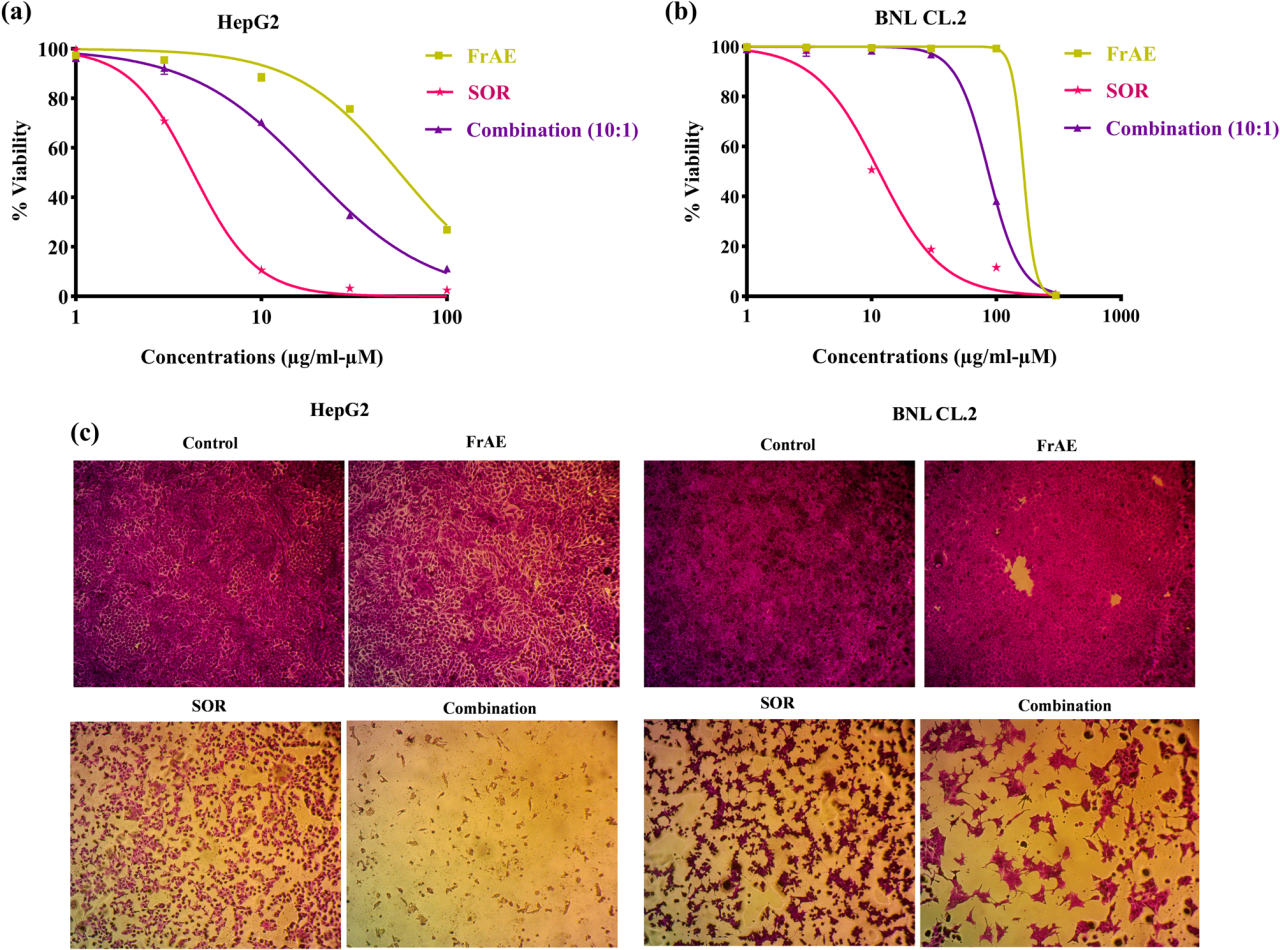
Cytotoxic and chemomodulatory effects of frankincense aqueous extract (FrAE) and sorafenib (SOR), tested individually at concentrations of 1,3,10,30, and 100 µg/ml and µM, respectively, in HepG2 cells **(a)** and at concentrations of 1,3,10,30, 100, and 300 µg/ml and µM, respectively, in BNL CL.2 cells **(b)**, as well as in an equitoxic combination ratio (10:1) on both cell lines following 72 h of treatment. Cell viability was evaluated using the SRB assay, and the data are expressed as the mean ± SD (n = 3). **(c)** Representative microscopic images displaying changes in cell density in HepG2 and BNL CL.2 cell lines following 72 h exposure to 100 µg/ml of FrAE, 30 µM of SOR, and their combination (100 µg/ml FrAE + 10 µM SOR), corresponding to a 10:1 combination ratio, compared with untreated control cells. Cells were stained with SRB dye and visualized using an inverted light microscope at 100 × magnification.

HepG2 liver cancer cells were treated with FrAE and the standard chemotherapeutic agent SOR for 72 h over a concentration range of 1–100 μg/ml and 1– 100 μM, respectively. Cell viability was evaluated using the SRB assay **(**Fig. 9a and c**)**. Both FrAE and SOR showed dose-dependent cytotoxicity, with IC_50_ values of 55.48 ± 1.33 μg/ml and 4.28 ± 0.92 µM, , respectively, indicating cytotoxic activity with an approximate activity ratio of 10:1. When combined at equitoxic concentrations, FrAE significantly enhanced the cytotoxic effect of SOR, as evidenced by a decrease in SOR’s IC_50_ value to 1.84 ± 0.62 μM, accompanied by a concomitant reduction in FrAE’s IC_50_ to 18.44 ± 1.25 μg/ml.

Interestingly, the cytotoxic effects of both FrAE and SOR appeared to be more pronounced in HepG2 cancer cells than in BNL CL.2 normal liver cells, which were treated at concentration of 1– 300 μg/ml and 1– 300 μM, respectively **(**Fig. 9b and c**)**. The IC_50_ values were 165.6 ± 2.71 μg/ml and 11.46 ± 1.19 μM for normal hepatocytes (BNL CL.2), with selectivity indexes (SI) of 2.98 and 2.67, respectively, indicating their relative safety (Table 3). Even when combined at equitoxic concentrations, FrAE and SOR showed IC_50_ values of 89.5 ± 2.22 μg/ml and 8.9 ± 1.13 μM, respectively, in BNL CL.2 cells, which were significantly higher than their IC values in HepG2 cells (FrAE: 18.44 ± 1.25 μg/ml and SOR: 1.84 ± 0.62 μM), underscoring the safety of the combination on normal liver cells and its selectivity towards liver cancer cells.

**Table 3:** Comparison of the selectivity index (SI) of FrAE, SOR, and their combination on BNL CL.2 (normal hepatocytes) versus HepG2 (liver cancer cells). Higher SI values indicate greater preferential cytotoxicity toward malignant cells with reduced toxicity to normal cells. The FrAE/SOR combination exhibits the highest SI.

Extract/Compound	Selectivity index (SI)
FrAE	2.98
SOR	2.67
Combination	4.85

The potent anticancer activity observed with frankincense can be attributed to its largely untapped phytochemicals, including volatile oil, polysaccharides, monoterpenes, diterpenes, and lipophilic pentacyclic triterpene acids^12,61^. These bioactive compounds have demonstrated promising anticancer activity, with a tendency to exhibit fewer and less severe side effects compared to conventional chemotherapeutic agents^62^.

**Fig. 10:**
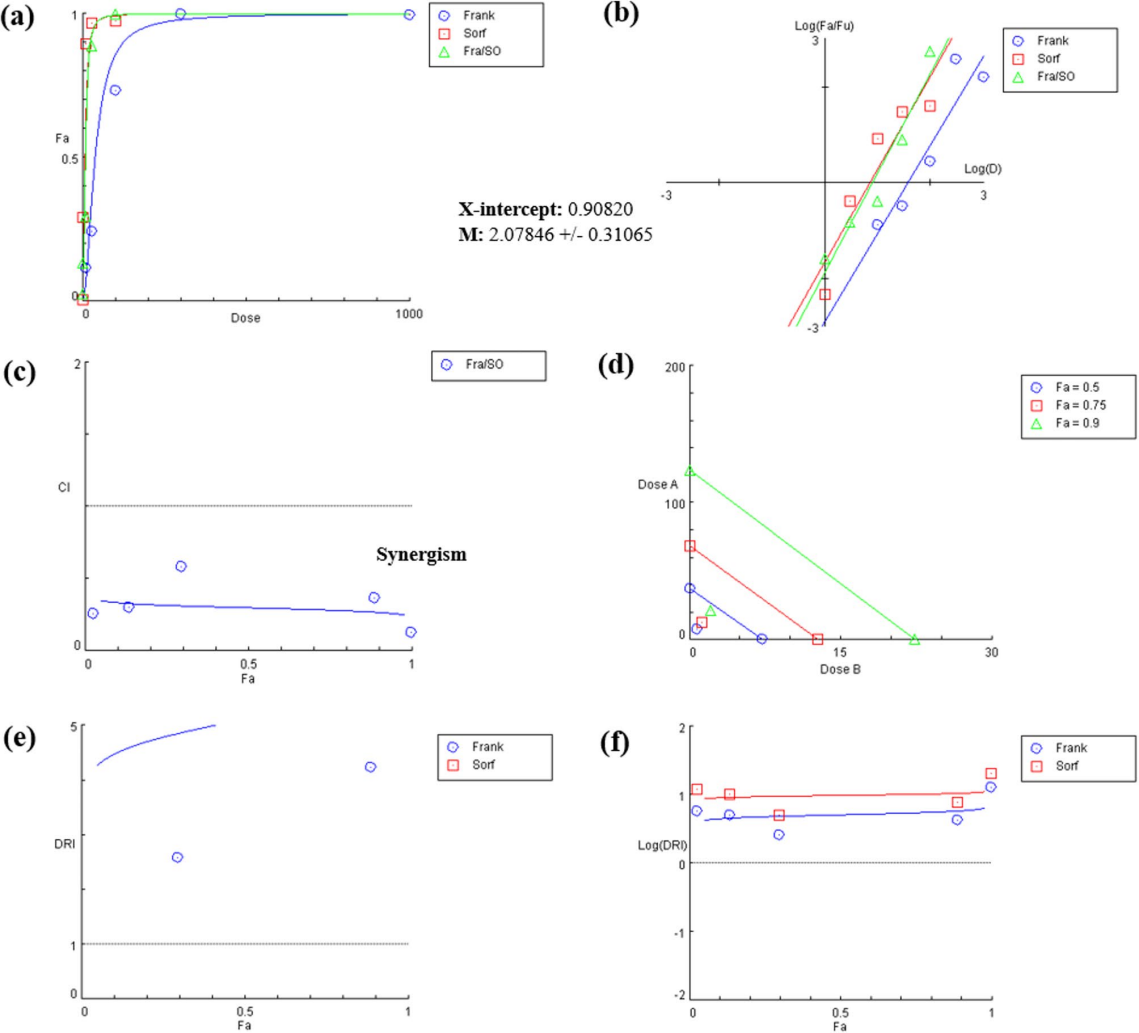
CompuSyn Report for Constant Combination of frankincense aqueous extract and sorafenib (FrAE/SOR). **(a)** Dose–Effect curve, **(b)** Median-Effect Plot. The IC_50_ value is the anti-log of the x-axis intercept, and the m value is the slope of the plot. **(c)** Combination Index (CI) Plot. The synergistic, additive, and antagonistic effects of the combination are defined as CI < 1, CI = 1, and CI > 1, respectively. The 5 combination data points are on the synergism side, with CI < 1. **(d)** Normalized Isobologram. Isobolograms for 50% (Fa 0.5), 75% (Fa 0.75), and 90% (Fa 0.9) inhibition are shown. The data points on the diagonal line indicate an additive effect, those on the lower left indicate synergism, and those on the upper right indicate antagonism. Here, IC_50_, IC_75_ and IC_90_ showed synergism. **(e)** and **(f)** Dose reduction index (DRI) Plot.

Further evaluation of the synergistic effect between SOR and FrAE was confirmed using CompuSyn software.**Further evaluation of the synergistic effect between SOR and FrAE was confirmed using CompuSyn software.** Cytotoxicity was examined in HepG2 cancer cells after treatment with different concentrations of SOR and FrAE at a ratio of 1 (SOR): 10 (FrAE) (Fig. 10a and b). The CI values for all tested doses were < 1, indicating synergism between the two agents (Fig. 10c and d). The CI value of FrAE /SOR at the IC_50_% was 0.298, indicating strong synergism (Table 4). Additionally, a drastic reduction was observed in the SOR dose required to achieve the same level of inhibition when combined with FrAE (Fig. 10e and f). According to the calculated dose reduction index (DRI), it requires 80% less SOR and 90% less FrAE to achieve 50% inhibition at a 1 (SOR): 10 (FrAE) ratio (Table 4).

**Table 4:** Combination index (CI) values for a constant combination of FrAE/SOR at a 10:1 ratio in HepG2 cells, and its effect on the dose reduction of FrAE and SOR at different fractional effect (Fa) levels, 50% (Fa = 0.5) and 97% (Fa = 0.97) growth inhibition.

	Drug/Combination	CI value	Dose of FrAE	Dose of SOR
Data for Fa = 0.5 (at 50% inhibition)	FrAE		37.6047	
	SOR			7.21070
	FrAE /SOR	0.29774	7.35881	0.73588
Data for Fa = 0.97 (at 97% inhibition)	FrAE		247.275	
	SOR			43.1720
	FrAE /SOR	0.24924	39.1868	3.91868

### Flow cytometric analysis

#### Cell cycle modulation in HepG2 cells by frankincense and sorafenib, alone or in combination

**Fig. 11:**
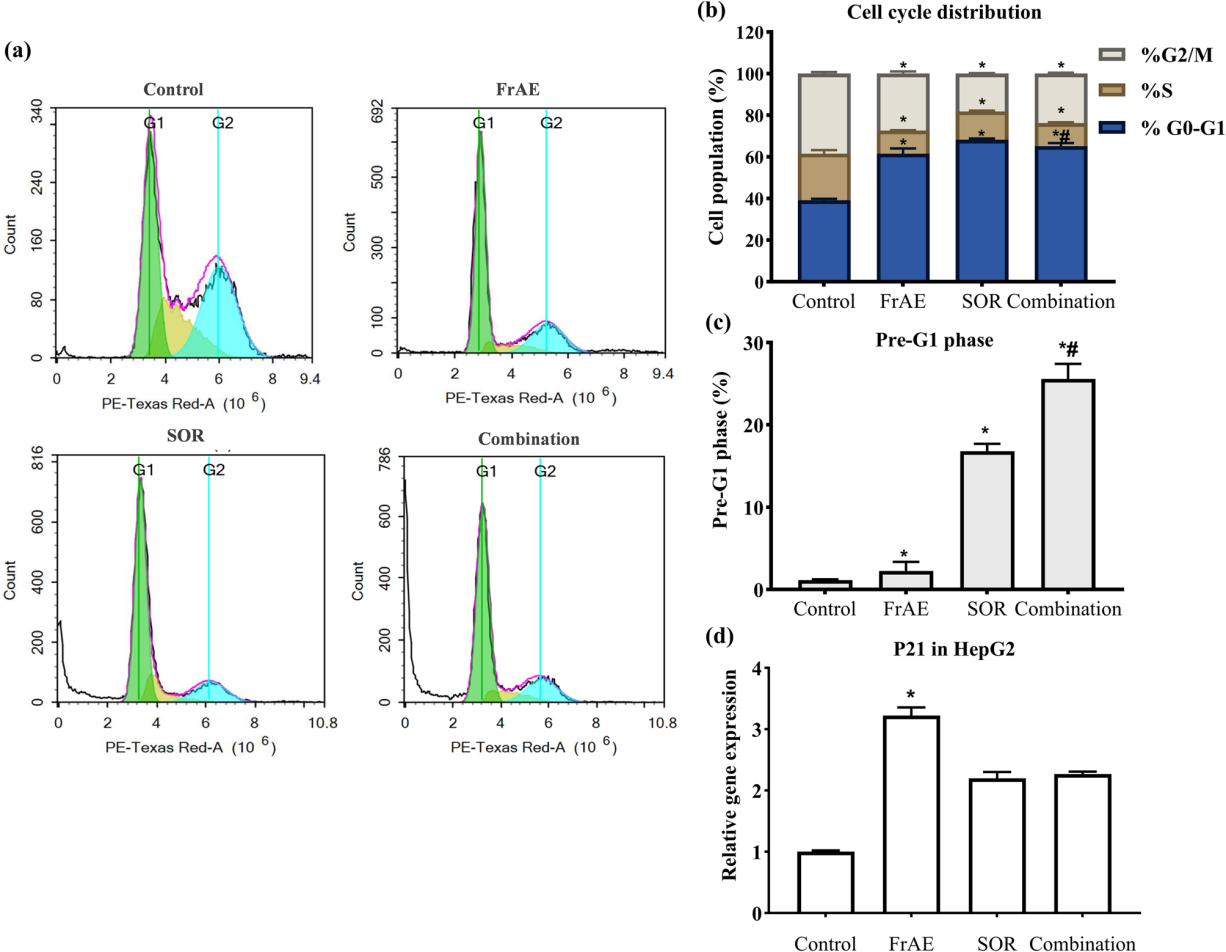
**(a)** Cell cycle distribution of HepG2 cells following 48 h of treatment with frankincense aqueous extract (FrAE, 55.48 µg/ml), sorafenib (SOR, 4.28 µM), and their combination (18.44 µg/ml FrAE + 1.84 µM SOR), corresponding to a 10:1 combination ratio treatment, as determined using DNA cytometry analysis compared with control (untreated cells). **(b)** The percentage of cells in each phase of the cell cycle was depicted as a bar graph of mean ± SD; n = 3. **(c)** The pre-G1 phase was plotted as the percentage of the total cell population.*Statistically significant from control at p < 0.0001, ^**#**^significantly different from Sorafenib group at p < 0.05, and **(d)** mRNA expression levels of the cell cycle-related gene P21 were assessed by qPCR after 24 h of treatment. *Statistically significant from control, ^**#**^significantly different from Sorafenib group at p ≤ 0.0001.

Cell proliferation is closely related to the cell cycle process^63^, and dysregulation of cell cycle machinery is a hallmark of various malignancies, including HCC^64,65^. Cancer cells often evade cell cycle checkpoints to avoid cell cycle arrest and/or apoptosis. Therefore, triggering cell cycle arrest is a crucial mechanism in determining how cancer cells respond to chemotherapeutic agents^66^. DNA flow cytometry was used to assess the cytotoxic effects of FrAE and SOR, alone or in combination, on the cell cycle distribution of the HepG2 cell line **(**Fig. 11**)**.

FrAE treatment demonstrated a potent anti-proliferative effect in HepG2 cells, as evidenced by a significant increase (p < 0.0001) in the proportion of cells in the G0/G1 Phase (non-proliferating cell fraction), rising from 39.12 ± 0.7% in control cells to 61.53 ± 2.5%. Conversely, FrAE markedly decreased the percentage of HepG2 cells in the S phase (DNA synthesis phase), which declined from 22.45 ± 1.7% of control to 11.14 ± 0.27%, along with a significant decrease in the G2/M phase population (mitotic phase), from 38.43 ± 0.8% to 27.33 ± 1.1%. These alterations in cell cycle distribution did not induce a significant increase in cell death, as evidenced by the minimal change in the pre-G1 phase population, from 1.15 ± 0.09% in control cells to 2.26 ± 1.11%.

Treatment with SOR showed a cell cycle distribution pattern in HepG2 cells similar to that elicited by FrAE treatment, albeit with different magnitudes, as demonstrated by a significant increase (p < 0.0001) in the G0/G1 phase population to 68.24 ± 0.6%, concurrent with a marked reduction in both the S-phase and G2/M phase populations to 13.59 ± 0.4% and 18.17 ± 0.3%, respectively, compared with control cells (Fig. 11a and b**)**. Unlike FrAE, these changes in cell cycle distribution were associated with a substantial increase in cell death, as indicated by a pronounced elevation in the pre-G1 phase population to 16.78 ± 0.9% **(**Fig. 11c**)**.

Combining SOR with FrAE induced mitotic arrest, as evidenced by a significant increase (p < 0.01) in the G2/M phase population from 18.17 ± 0.33% to 23.76 ± 0.48% compared with SOR alone **(**Fig. 11a and b**)**. This change in cell cycle distribution was further reflected in a more significant induction of cell death, as evidenced by an increase in the pre-G1 phase from 16.78 ± 0.92% to 25.58 ± 1.84% **(**Fig. 11c**)**. These findings suggest that the combined treatment in HepG2 cells induces the accumulation of cells in the mitotic phase, which can bypass the G2/M cell cycle checkpoint, resulting in premature entry into mitosis and ultimately, cell death through a mitotic catastrophe^67,68^. The FrAE/SOR combination did not result in any significant changes in the G0/G1 or S-phase populations compared with SOR treatment alone.

In general, the G0/G1 and G2/M phases are critical checkpoints in cell cycle regulation^69^. The induction of G0/G1 arrest with frankincense treatment alone, as well as the G2/M arrest observed with combined FrAE/SOR treatment, suggests that FrAE exerts its anticancer effect in HCC by disrupting the cell cycle at various stages. This is consistent with previous studies showing that frankincense has in vitro anticancer activities across a variety of cancers by triggering cell cycle arrest^12,60,70^.

Given that cell cycle arrest is a key approach to effective cancer therapy, these findings suggest that frankincense, either alone or in combination with SOR, may be a promising therapeutic agent in the management of HCC.

Our RT-PCR analysis revealed that treatment of HepG2 cells with FrAE (3.22-fold), SOR (2.19-fold), and the combination (FrAE/SOR; 2.26-fold) for 24 h significantly increased p21 mRNA expression compared to untreated control cells **(**Fig. 11d**).** Notably, the FrAE-treated group showed the highest induction, suggesting that FrAE predominantly mediates G1-phase cell cycle arrest via p21 gene induction^71^. In addition, the relatively lower p21 induction in the SOR and combination groups may reflect the role of p21 in inhibiting the cyclin A/CDK1 and cyclin B/CDK1 complexes, thereby blocking the G2/M transition and triggering cell cycle arrest^72^. These findings highlight the critical regulatory role of p21 in cell cycle control.

### Effect of frankincense and sorafenib alone or combined on apoptotic and necrotic cell death in HepG2 cells

Induction of apoptosis is a well-established, promising approach in the treatment of numerous malignancies, including HCC^73,74^. To discern whether the cytotoxic and synergistic effects of FrAE, SOR, and their combination treatment are mediated through apoptosis or necrosis, HepG2 cells were treated with the IC_50_ concentrations of FrAE and SOR alone and in combination for 48 h, cell death modes were then evaluated using PI and Annexin-V/FITC staining coupled with flow cytometry.

**Fig. 12:**
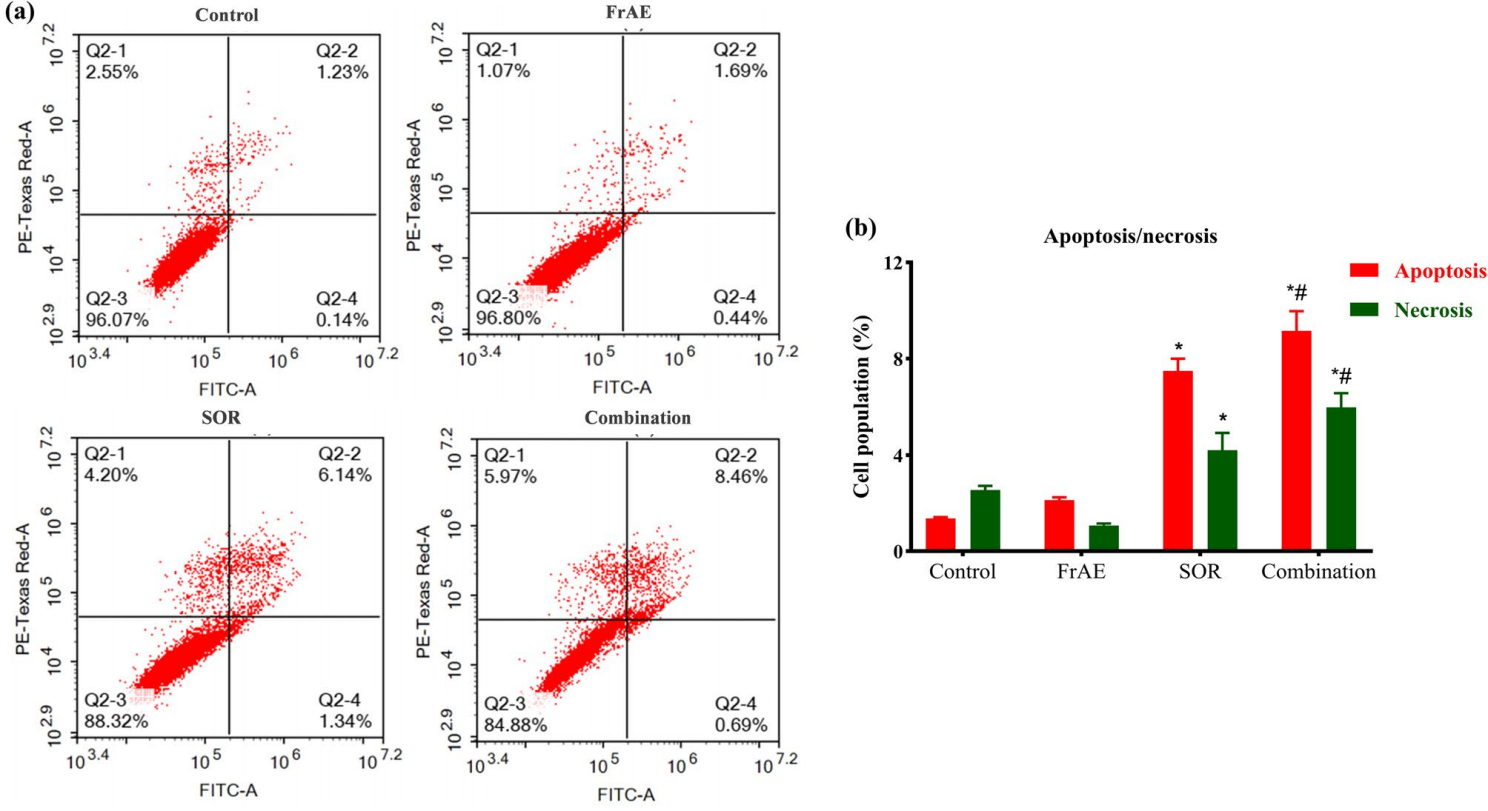
**(a)** Apoptosis/necrosis assessment by flow cytometry in HepG2 cells pretreated with frankincense aqueous extract (FrAE, 55.48 µg/ml), sorafenib (SOR, 4.28 µM), and their combination (18.44 µg/ml FrAE + 1.84 µM SOR), corresponding to a 10:1 combination ratio treatment for 48 h and stained with PI /annexin V-FITC. **(b)** The percentages of different cell populations undergoing apoptosis and necrosis were quantified and expressed as a percentage of total events. Data are presented as mean ± SD; n = 3. * Statistically significant from control, ^**#**^significantly different from sorafenib group at p < 0.0001.

As shown in Fig. 12, SOR induced both apoptotic and necrotic cell death in HepG2 cells, with apoptosis being the predominant mechanism **(**Fig. 12b**)**. SOR treatment alone resulted in approximately 12% cell death, comprising 7.48 ± 0.51% apoptosis and 4.2 ± 0.729% necrosis compared to control untreated HepG2 cells, which exhibited only 1.37 ± 0.05% apoptosis and 2.55 ± 0.17% necrosis. In comparison, FrAE treatment alone did not induce apoptotic or necrotic cell death, as observed in control cells **(**Fig. 12a and b**)**.

Compared with SOR treatment alone, combining SOR with FrAE induced additional cell death, predominantly via apoptosis. The combination resulted in approximately 15% total cell death, comprising 9.15 ± 0.82% apoptosis and 5.97 ± 0.59% necrosis in our HepG2 model, suggesting that frankincense’s anticancer effect may involve mechanisms beyond apoptosis. This finding contrasts with prior literature; Liu et al. (2002) reported that keto-boswellic acids from frankincense induce G1 arrest and caspase–8–dependent apoptosis in HepG2 cells^17^. Thus, the relatively low level of apoptosis observed here should be interpreted cautiously, as experimental differences (cell type, extract composition, or concentrations) could explain these discrepancies. Previous studies have shown that frankincense constituents exert anticancer activities by activating apoptotic pathways in melanoma^75^, breast cancer^60^, lung cancer^76^, and oral squamous cell carcinoma (OSCC)^77^. We note that our findings do not imply that frankincense never induces apoptosis; instead, in this specific HepG2 assay, apoptosis was not the dominant mechanism of action. This finding is consistent with prior research on bladder cancer, which has shown that frankincense does not induce apoptosis across all cell lines^70^. These data highlight that frankincense’s pro-apoptotic activity is tumor cell type specific.

### Role of autophagy in the synergistic effect of combined treatment

With more in-depth insights into cell death pathways, growing evidence suggests that autophagy can also induce cell death in a context-dependent manner, rather than surving the generally perceived cytoprotective role^78^. In HCC, particularly in aggressive or invasive types, autophagy-based therapy, either alone or in combination with conventional therapies, has opened new opportunities for treatment^79,80^.

**Fig. 13:**
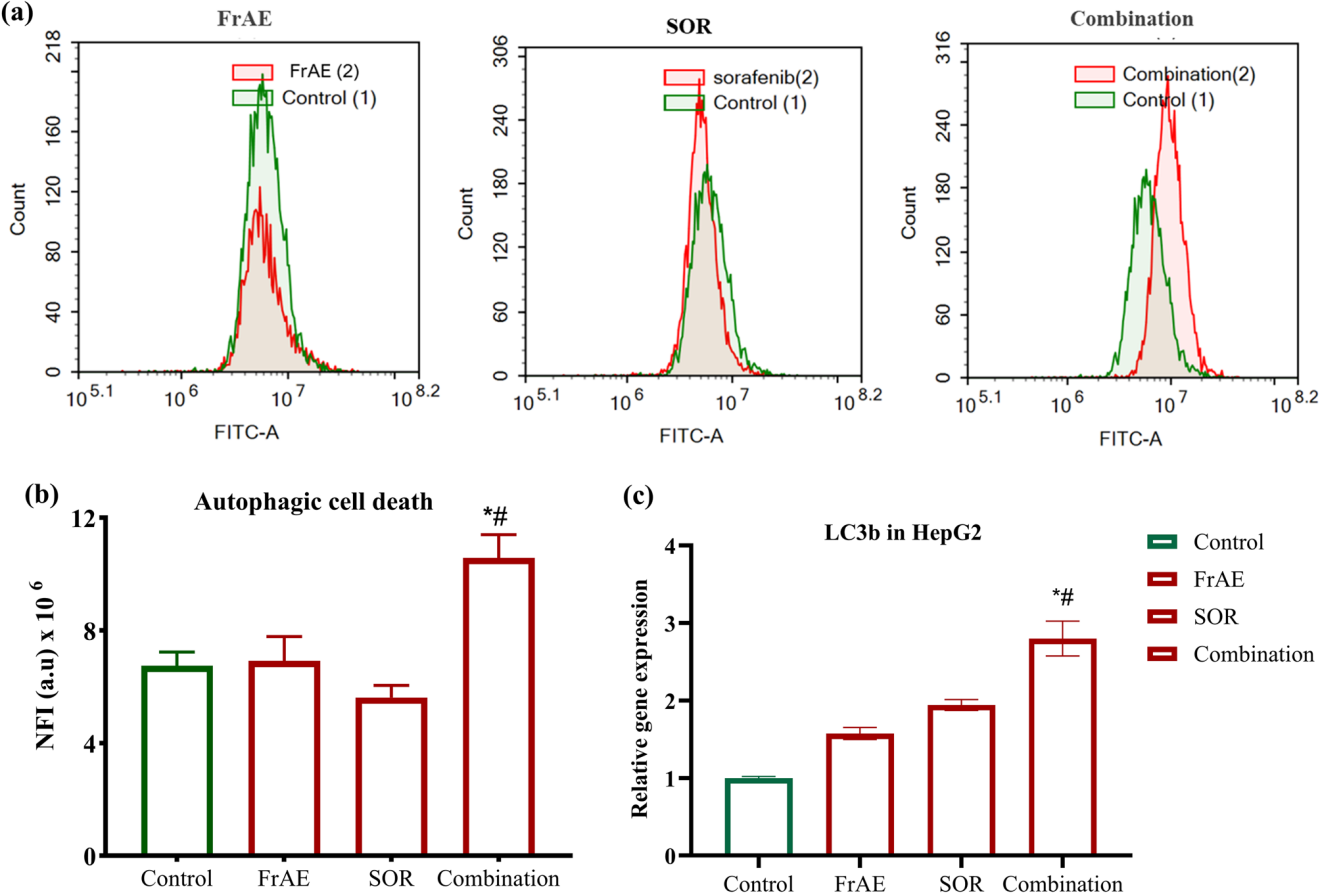
**(a)** Autophagic cell death evaluation in HepG2 cells after exposure to frankincense aqueous extract (FrAE, 55.48 µg/ml), sorafenib (SOR, 4.28 µM), and their combination (18.44 µg/ml FrAE + 1.84 µM SOR), corresponding to a 10:1 combination ratio treatment for 48 h. Cells were stained with acridine orange dye. **(b)** Net fluorescent intensity (NFI) was plotted relative to the basal fluorescence of untreated HepG2 cells. **(c)** mRNA expression levels of the autophagy-related gene LC3B (MARCH 1) were assessed by qPCR after 24 h of treatment. Data are displayed as mean ± SD; n = 3. *Statistically significant from control, ^**#**^significantly different from Sorafenib group at p ≤ 0.0001.

Therefore, autophagy was evaluated in HepG2 cells following a 48-h treatment with FrAE, SOR, and their combination, using acridine orange dye coupled with flow cytometry **(**Fig. 13**)**.

FrAE treatment alone did not induce any notable change in net fluorescent intensity (NFI) compared to untreated control HepG2 cells. However, SOR treatment led to a reduction in autophagic activity, as indicated by a decline in net fluorescent intensity (NFI) from 6.74 ± 0.49 × 10^6 NFI in control cells to 5.61 ± 0.44 × 10^^6^** (**Fig. 13a and b**)**. Interestingly, combining SOR with FrAE induced a significant increase (p < 0.0001) in the autophagic signal, with NFI rising to 10.57 ± 0.83 × 10^6^, compared to 5.61 ± 0.44 × 10^6^ with SOR alone. These findings suggest that autophagy plays a pivotal role in mediating the synergistic effect of the SOR and FrAE combination, underscoring its potential as a therapeutic approach in HCC. Moreover, these findings support recent experimental evidence indicating that frankincense inhibits cancer progression by inducing autophagy^81^.

To validate these findings, LC3B (MAP1LC3B) mRNA expression, a commonly used surrogate marker for cellular autophagy^82^ was measured. After 24 h of treatment, the combination therapy significantly (P ≤ 0.05) increased LC3B mRNA expression in HepG2 cells (2.79-fold), consistent with flow cytometry results **(**Fig. 13c**).** In contrast, FrAE and SOR individually resulted in modest increases in LC3B expression (1.57-fold and 1.87-fold, respectively). The discrepancy between these transcriptional findings and the flow cytometry data suggests a transient induction of autophagy-related gene transcription that does not necessarily translate into sustained or functional autophagy activity. This difference is most likely due to time-dependent regulation, as RT-PCR analysis was performed at 24 h and flow cytometry assessment at 48 h, highlighting the dynamic nature of autophagy regulation.

### FrAE /SOR suppressed the migration of HepG2 cells

The main challenge in HCC management is tumor recurrence and metastasis, which are driven by dysregulated cell migration and invasion^83^. Despite the availability of multiple therapies aimed at limiting tumor growth, they are often unsuccessful in effectively treating metastatic cancer. Therefore, identifying effective strategies to control metastasis is crucial for improving HCC treatment outcomes^2^.

Accordingly, a scratch assay, one of the most widely used methods for evaluating the effects of compounds on cell migration and cancer metastasis^84^, was employed to assess the potential anti-migratory impact of FrAE, SOR, and their combination on the daily migration of HepG2 cells. Sub-IC_50_ concentrations of FrAE (25 µg/ml), SOR (2 µM), and their combination (9 μg/ml FrAE + 0.9 µm SOR, corresponding to a 10:1 ratio) were used in the assay. Images of the scratch width were captured daily from time zero until the complete closure of control cells after 96 h.

At 0 h, there was no significant difference in initial scratch width among groups (p > 0.5), supporting the consistency of the scratch technique. As shown in Figs. 13A and B, untreated HepG2 cells exhibited clear migratory behavior, with significant scratch closure of 34.28 ± 1.15%, 54.47 ± 2.54%, and 87.09 ± 1.16% at 24, 48, and 72 h, respectively. Complete scratch closure at 96 h represented 100% migration.

Interestingly, treatment with FrAE alone effectively inhibited HepG2 migration compared with the control group, displaying significantly lower scratch closure percentages of 10.55 ± 2.54%, 13.46 ± 1.75%, and 37.52 ± 2.31% at 24, 48, and 72 h, respectively. However, complete scratch closure was observed at 96 h.

**Fig. 14:**
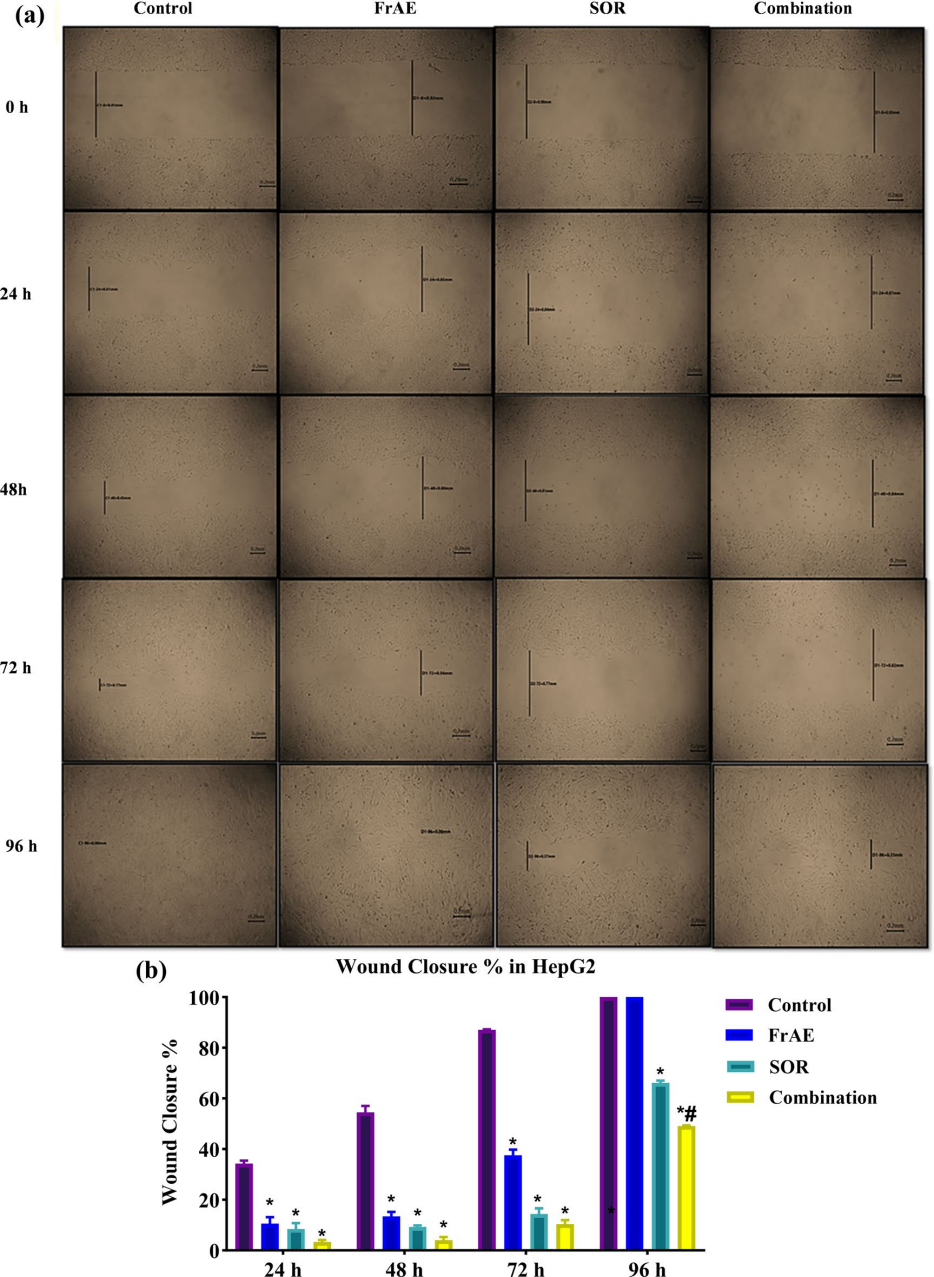
**(a)** Evaluation of the anti-migratory activity of frankincense aqueous extract (FrAE, 25 µg/ml), sorafenib (SOR, 2 µM), and their combination (9 μg/ml FrAE + 0.9 µm SOR), corresponding to a 10:1 combination ratio treatment on HepG2 cell migration using the scratch assay. Widths were assessed at 24, 48, 72, and 96 h post-treatment. **(b)** Data were plotted as wound closure percentage at each time point and represented as mean ± SD; n = 3. *Statistically significant from control, ^**#**^significantly different from the sorafenib group at p < 0.0001.

SOR significantly inhibited HepG2 migration at all time points (P < 0.0001) compared with both the control and FrAE groups. Scratch closure percentages were 8.49 ± 2.32%, 9.22 ± 0.67% 14.37 ± 2.23%, and 66.11 ± 0.94% at 24, 48, 72, and 96 h, respectively, with no complete closure even after 96 h **(**Fig. 14a and b**)**.

Notably, the combination of FrAE and SOR exhibited the most significant inhibition (P < 0.0001 compared to control), resulting in the lowest scratch closure percentages at all-time points: 3.29 ± 0.79%, 4.02 ± 1.27%, 10.38 ± 1.56%, and 49.1 ± 0.26% at 24, 48, 72, and 96 h, respectively. These findings suggest a synergistic effect between FrAE and SOR in suppressing HepG2 cell migration **(**Fig. 14b**)**.

Frankincense, either alone or in combination with other phytochemicals, has been shown to inhibit the invasion and migration of various malignancies^13,58,59,85^.

Consistent with these findings, our results demonstrated that the combination of FrAE and SOR exerts anti-migratory activity in HCC, supporting this combination as a novel therapeutic strategy to inhibit HCC initiation and progression. Further research is warranted to elucidate the underlying mechanisms and to evaluate the in vivo and clinical efficacy of the combination.

### Molecular docking analysis of major constituents

Although treatment with FrAE alone did not induce apoptotic or autophagic cell death, it enhanced the cytotoxic activity of SOR. To elucidate the potential role of FrAE bioactive compounds in potentiating SOR-induced apoptosis and autophagy, molecular docking analysis was conducted. In the present study, molecular docking was performed to investigate the interaction between terpenoids in FrAE and apoptosis and autophagy markers, by identifying amino acid residues and protein–ligand binding energies. Nine terpenoids, with PubChem IDs, Suppl. Table 1, exhibiting reported cytotoxic activities from different classes (2 diterpenes, 2 ursane triterpenes, 2 lupane triterpenes, 2 tricullane triterpene, and 1 oleane triterpene) were selected for docking into the active sites of apoptosis markers (BCL-2 and p53), and autophagy markers (mTOR and LC3C).

When p53 is activated, it downregulates anti-apoptotic proteins, such as BCL-2, and promotes the expression of pro-apoptotic Bcl-2 family members, including BAX and PUMA. These proteins trigger the caspase cascade, which leads to apoptosis by promoting cytochrome c release and mitochondrial outer membrane permeabilization^86,87^. mTOR is a protein kinase that regulates various cellular processes, including autophagy. Inhibitors of mTOR can induce autophagy, potentially leading to autophagic cell death in cancer cells^88^. LC3C is a protein that mediates selective autophagy by targeting specific proteins for degradation within the cell^89^. Therefore, the development of innovative p53 and LC3C regulators as well as BCL-2, and mTOR inhibitors derived from phytochemicals is significant. The interactions (interacting residues forming H-bonds and hydrophobic interactions) between proteins and ligands, as well as the binding energies, were used to analyze the data. The ligands were listed in order of their binding energy (kcal/mol) with different proteins (Table 5).

**Table 5:** Comparative molecular docking scores (binding free energies, kcal/mol) of co-crystallized reference ligands (as positive controls) and FrAE-derived terpenoids against BCL-2 and p53 (apoptosis-associated targets), as well as mTOR and LC3C (autophagy-associated targets).

	Class	Name	Apoptosis		Autophagy	
			BCL-2	p53	mTOR	LC3C
		*Co-crystallized ligand* (internal ligand)	-12.3	-7.3	-5.6	-3.8
1	Ursane triterpene	11-Keto boswellic acid	-8.4	-5.7	-5.2	-5.2
2		3-O-Acetyl 11-keto boswellic acid	-8.2	-5.4	-4.7	0.8
3	Lupane triterpene	3-Acetyl-27-hydroxy-lupeolic acid	-7.3	-5.1	-4.3	0.5
4		Lupenone	-8.1	-5.5	-6.0	-4.2
5	Tricullane triterpene	3-hydroxytirucallic acid (α-Elemolic acid)	-7.7	-6.2	-5.3	-4.5
6		3-oxo-tirucallic acid (β-Elemonic acid)	-7.8	-6.2	-5.2	-4.2
7	Oleane triterpene	α-Amyrenone	-8.9	-5.6	-5.6	-4.7
8	Diterpene	Boscartin C	-7.6	-4.5	-3.9	-5.0
9		Boscartol G	-7.5	-5.4	-5.2	-6.0

**Fig. 15:**
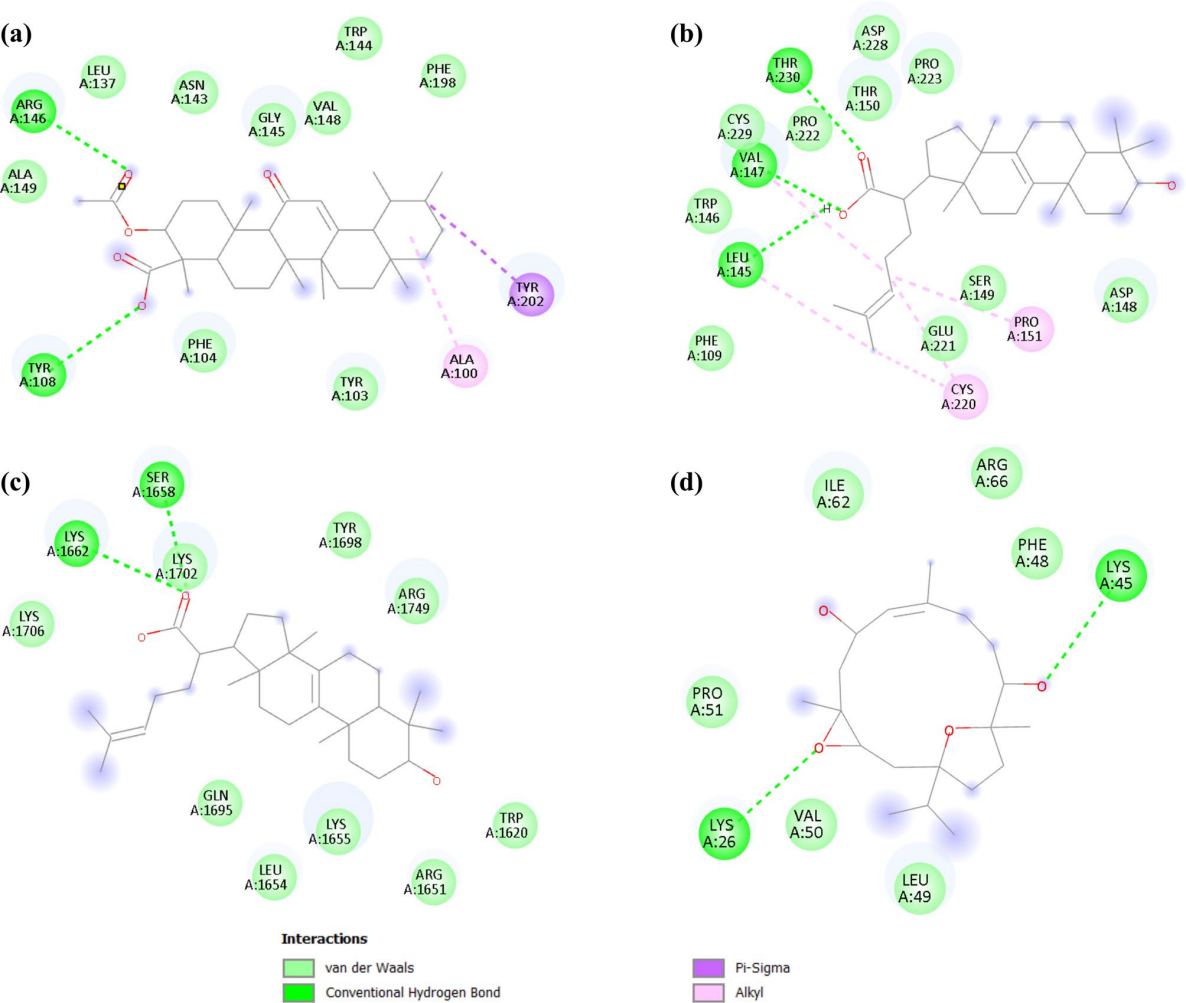
A 2D display representing the binding interactions of compounds with the highest binding energies: **(a)** 3-O**-**acetyl 11-keto boswellic acid in the active site of BCL-2, **(b)** α-Elemolic acid in the active site of p53, **(c)** α-Elemolic acid in the active site of mTOR, and **(d)** Boscartin C in the active site of LC3C.

The results revealed that all estimated binding free energies were negative, ranging from -3.9 to -8.9 kcal/mol, indicating favorable interactions. Among the ursane-type triterpenes, KBA and 3-O-Acetyl 11-keto boswellic acid (AKBA) (Fig. 15a) had the most negative free energy of binding to BCL-2 (–8.4 and –8.1 kcal/mol, respectively), suggesting strong predicted binding to this target. For the Tricullane-type triterpenes, α-Elemolic acid (Fig. 15b), and β-Elemonic acid showed binding energies of –6.2 kcal/mol, which are comparable to that of the co-crystallized p53 ligand, indicating appreciable affinity for the p53 active site (–7.3 kcal/mol). Regarding mTOR, Lupenone (–6.0 kcal/mol) and α-Elemolic acid (–5.3 kcal/mol) showed the most favorable docking energies (Fig. 15c). For LC3C, Boscartin C (–5.0 kcal/mol) formed favorable hydrogen bonds (Fig. 15d).

Table 6 highlights Ligand/protein interactions with amino acid residues of ligands with the highest binding energies.

**Table 6:** Detailed molecular docking interaction profile of co-crystallized reference ligands (as positive controls) and selected FrAE-derived terpenoids with key apoptosis- and autophagy-related target proteins (BCL-2, p53, mTOR, and LC3C), illustrating the interacting amino acid residues within each protein-binding pocket along with the corresponding interaction types.

	Ligands	Amino acids	Type of interaction
BCL-2	Sonrotoclax (Co-crystallized Ligand)	ARG146* ALA100* TYR202* TYR103	π -alkyl C-H bond π -π stacked π -alkyl
	Keto boswellic acid (KBA)	TYR202*	Conventional H-bond
	Acetyl keto boswellic acid (AKBA)	ARG146* TYR108 TYR202* ALA100*	Conventional H-bond Conventional H-bond π -sigma Alkyl
p53	2-(4-(4-fluorophenyl)-5-(1H-pyrrol-1-yl)-1H-pyrazol-1-yl)-N,N-dimethylethanamine (Co-crystallized Ligand)	ASP228 PRO223 VAL147^^^ LEU145^^^ CYS220^^^ PRO151^^^	Halogen (Fluorine) π –alkyl π –alkyl π –alkyl π –Donor H-bond π –Donor H-bond
	3-hydroxytirucallic acid (α-Elemolic acid)	THR230 VAL147^^^ LEU145^^^ PRO151^^^ CYS220^^^	Conventional H-bond Conventional H-bond Conventional H-bond Alkyl Alkyl
	3-oxo-tirucallic acid (β-Elemonic acid)	THR230 VAL147^^^ PRO151^^^ CYS220^^^	Conventional H-bond Alkyl Alkyl Alkyl
mTOR	Inositol hexakisphosphate (Co-crystallized Ligand)	LYS1662^#^ LYS1655 SRE1658^#^	Conventional H-bond, Salt bridge Salt bridge Conventional H-bond
	Lupenone	LYS1662^#^	Alkyl
	3-hydroxytirucallic acid (α-Elemolic acid)	LYS1662^#^ SRE1658^#^	Conventional H-bond Conventional H-bond
LC3C	Citric acid (Co-crystallized Ligand)	LEU A49 LYS A45^•^ ARG A66	Conventional H-bond Conventional H-bond Conventional H-bond Salt bridge Conventional H-bond Salt bridge
	Boscartin C	LYS A45^•^ LYS A26	Conventional H-bond Conventional H-bond
	3-hydroxytirucallic acid (α-Elemolic acid)	LYS A45^•^	Conventional H-bond

^*,**^**, #, •^refer to amino-acid residues that are in common between the co-crystallized Ligands and compounds.

Ligand/protein interactions of the co-crystallized ligands and remaining compounds are illustrated in the supplementary material file (Suppl. Table 2–3 and Suppl. Figure 1–9). Although the oleane triterpene, α-amyrenone, has a high free energy of binding towards tested proteins, it interacts with the active sites of proteins (mTOR and BCL-2) through multiple weak van der Waals forces (Suppl. Figure 3B).

Overall, docking analysis suggested that these terpenoids may bind to and potentially modulate key regulators of apoptosis and autophagy; however, these results provide only in silico insights rather than proof of mechanism^90,91^. The compounds were also only tentatively identified in FrAE. Thus, the docking results require further experimental validation. Notably, the docking results were partially consistent with our biological assays, which showed that FrAE alone did not induce strong apoptosis or autophagy; however, combining FrAE with SOR did induce a significant increase in apoptotic and autophagic cell death. In addition, the docking results were partially aligned with previously reported biological activities of specific terpenoids in frankincense. For example, KBA and AKBA have been reported as BCL-2 inhibitors^92^, and our docking study showed strong predicted binding of KBA and AKBA to BCL-2. These in silico insights complement our flow cytometry data but must remain speculative.

## Conclusion

This study provides evidence that the combination of frankincense aqueous extract (FrAE) with sorafenib exerts significant anticancer effects in HepG2 cells by inhibiting proliferation, migration, and invasion. These effects are likely mediated by the diverse terpenoid constituents of frankincense. FrAE demonstrated selective cytotoxicity toward cancer cells while sparing normal liver cells, highlighting its potential to reduce the adverse effects commonly associated with conventional chemotherapeutic agents. The observed synergism between FrAE and sorafenib suggests that this combination could serve as a promising therapeutic strategy for hepatocellular carcinoma (HCC), potentially enhancing efficacy while minimizing toxicity. However, given that these findings are based on a single in vitro model, further validation using additional HCC cell lines (e.g., Huh7 or Hep3B) and comprehensive in vivo studies is warranted to fully assess the translational potential of this combination therapy.

## Supplementary Information

Supplementary Information 1.

## Data Availability

The authors declare that the data supporting the findings of this study are available within the paper and its Supplementary Information files. Any raw data files in a format other than the original are available from the corresponding author upon reasonable request.
