# Supplementary material for: Synergistic anticancer activity of frankincense aqueous extract with sorafenib in HepG2 cells and its UHPLC–QTOF–MS/MS-based metabolomic profiling
Source: Sci Rep. 2026 Mar 27;16:10834. doi: 10.1038/s41598-026-42328-y (PMC13039205; doi:10.1038/s41598-026-42328-y)
Supplement: Supplementary file 1 — Supplementary Information 1. [file 41598_2026_42328_MOESM1_ESM.pdf]

UHPLC–QTOF–MS/MS Based Metabolic Profiling of Frankincense Aqueous Extract and its Synergistic Anticancer Effect with Sorafenib in HepG2 Cells.

Wesam Ragab[1*+], Kamel Mahmoud[1+] , Seham Salah El-Din[2], Osama M. Gomaa[2] , Rasha M. Allam[3], Abeer Sayed[4], Rabab Mohammed[4*].

[1]Pharmacognosy Department, Faculty of Pharmacy, MUST University, 6th October City, Giza 12566, Egypt.

[2]Pharmacognosy Department, Faculty of Pharmacy, Cairo University, Cairo 11562, Egypt.

[3]Pharmacology Department, Medical and Clinical Research Institute, National Research Centre, 33 El-Bohouth St., Dokki, P.O.12622, Cairo, Egypt.

[4]Pharmacognosy Department, Faculty of Pharmacy, Beni-Suef University, Beni-Suef 62514, Egypt.

* Corresponding authors: wesam.ragab@must.edu.eg Orcid no. https://orcid.org/0000-0002-1809-8005

rababmohammed@pharm.bsu.edu.eg https://orcid.org/0000-0001-9683-4250

[+] These authors contributed equally to this work.

Suppl. Table (1): PubChem IDs, structures, and docking scores of docked ligands.

					**Apoptosis**		**Autophagy**	
	**Class**	**Name**	**PubChem ID**	**Structure**	**BCL-2**	**P53**	**mTOR**	**LC3C**
		**Internal ligand**			**-12.3**	**-7.3**	**-5.6**	**-3.8**
1	Ursane triterpene	11-Keto boswellic acid	9847548	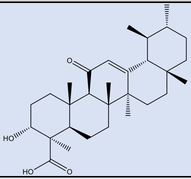	**-8.4**	-5.7	-5.2	**-5.2**
2		3-O-Acetyl 11-keto boswellic acid	11168203	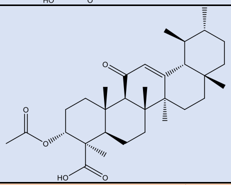	**-8.2**	-5.4	-4.7	0.8
3	Lupane triterpene	3-Acetyl-27-hydroxy-lupeolic acid	21575462	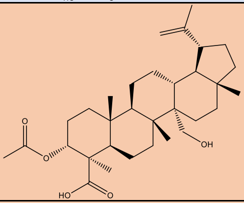	-7.3	-5.1	-4.3	0.5
4		Lupenone	92158	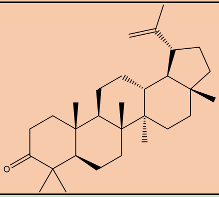	-8.1	-5.5	**-6.0**	-4.2
5	Tricullane triterpene	3-hydroxytirucallic acid (α-Elemolic acid)	441677	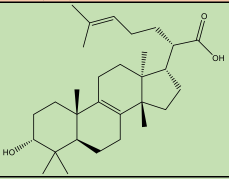	-7.7	-6.2	-5.3	-4.5
6		3-oxo-tirucallic acid (β-Elemonic acid)	15559100	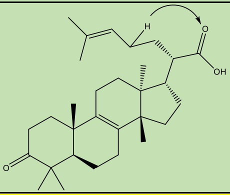	-7.8	-6.2	-5.2	-4.2
7	Oleane triterpene	α-Amyrenone	12306155	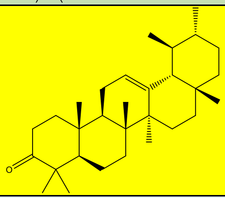	**-8.9**	-5.6	**-5.6**	**-4.7**
8	Diterpene	Boscartin C	319538644	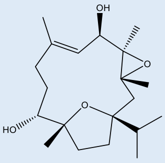	-7.6	-4.5	-3.9	-5.0
9		Boscartol G	275376549	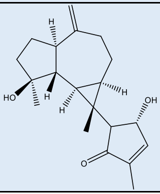	-7.5	-5.4	-5.2	**-6.0**

Suppl. Table (2): Docking scores, different amino acids bonded to docked compounds along with types of interaction in the active sites of BCL-2 and P53.

Apoptosis markers							
		**BCL-2** (8HOI)			**P53** (3ZME)		
	Compound	Docking Score (kcal/mol)	Amino acid	Type of interaction	Docking Score (kcal/mol)	Amino acid	Type of interaction
	**Internal ligand**	**-12.3**	**ARG146**<br>**ALA100**<br>TYR202<br>TYR103<br>GLN A99<br>**PHE112**<br>**MET115**	π -alkyl<br>C-H bond<br>π -π stacked<br>π –alkyl<br>Conventional H-bond<br>π –sigma<br>π -sigma	**-7.3**	ASP228<br>PRO223*<br>VAL147<br>LEU145<br>**CYS220**<br>**PRO151**	Halogen (Fluorine)<br>π –alkyl<br>π –alkyl<br>π –alkyl<br>π –Donor H-bond<br>π –Donor H-bond
1	Keto boswellic acid	-8.4	TYR202	Conventional H-bond	-5.7	PRO223*<br>PRO222	Carbon H-bond<br>Alkyl
2	Acetyl keto boswellic acid	-8.2	**ARG146**<br>TYR108<br>TYR202<br>**ALA100**	Conventional H-bond<br>Conventional H-bond<br>π -sigma<br>Alkyl	-5.4	PRO223*	Carbon H-bond
3	3-Acetyl-27-hydroxy-lupeolic acid	-7.3	TYR108	Conventional H-bond<br>π -sigma	-5.1	PRO222<br>THR150	Alkyl<br>Conventional H-bond
4	Lupenone	-8.1		multiple Van der Waals forces	-5.5	PRO222	Alkyl
5	3-hydroxytirucallic acid (α-Elemolic acid)	-7.7	PHE104	π -alkyl	**-6.2**	THR230<br>VAL147<br>LEU145<br>**PRO151**<br>**CYS220**	Conventional H-bond<br>Conventional H-bond<br>Conventional H-bond<br>Alkyl<br>Alkyl
6	3-oxo-tirucallic acid (β-Elemonic acid)	-7.8	PHE104	π -alkyl	**-6.2**	THR230<br>VAL147<br>**PRO151**<br>**CYS220**<br>LEU145	Conventional H-bond<br>Alkyl<br>Alkyl<br>Alkyl<br>Unfavorable acceptor-acceptor
7	α-Amyrenone	-8.9		multiple Van der Waals forces	-5.6	THR150	Conventional H-bond
8	Boscartin C	-7.6	**PHE112**<br>**MET115**	π –sigma<br>Alkyl	-4.5	THR150<br>ASP148	Conventional H-bond<br>Unfavorable acceptor-acceptor
9	Boscartol G	-7.5	**ALA100**<br>TYR103<br>TYR202	Alkyl & π -alkyl<br>Alkyl & π -alkyl<br>π donor H-bond	-5.4	THR150<br>VAL147<br>PRO223*	Conventional H-bond<br>Conventional H-bond<br>Alkyl

Suppl. Table (3): Docking scores, different amino acids bonded to docked compounds along with types of interaction in the active sites of mTOR and LC3C.

		**Autophagy marker**					
		**mTOR** (9F44)			**LC3C** (3WAM)		
	Compound	Docking Score (kcal/mol)	Amino acid	Type of interaction	Docking Score (kcal/mol)	Amino acid	Type of interaction
	**Internal ligand**	**-5.6**	**LYS1662**<br><br>**LYS1655**<br>SRE1658<br>TYR1698<br>LYS1702<br>**LYS1788**<br><br>ARG1749	Conventional H-bond<br>Salt bridge<br>Salt bridge<br>Conventional H-bond<br>Conventional H-bond<br>Salt bridge<br>Conventional H-bond<br>Salt bridge<br>Salt bridge	**-3.8**	**LEU A49**<br><br>**LYS A45**<br><br>ARG A66	Conventional H-bond<br>Conventional H-bond<br>Conventional H-bond<br>Salt bridge<br>Conventional H-bond<br>Salt bridge
1	Keto boswellic acid	-5.2	**LYS1655**	Conventional H-bond	-5.2	LYS A45<br>PRO A51	Unfavorable donor-donor<br>Alkyl
2	Acetyl keto boswellic acid	-4.7	ARG1749<br>**LYS1662**	Conventional H-bond<br>Alkyl	~~0.8~~	……..	……..
3	3-Acetyl-27-hydroxy-lupeolic acid	-4.3	**LYS1788**<br>ARG1651	Conventional H-bond<br>Alkyl	~~0.5~~	……..	……..
4	Lupenone	-6.0	**LYS1662**	Alkyl	-4.2	**LEU A49**	Alkyl
5	3-hydroxytirucallic acid (α-Elemolic acid)	-5.3	**LYS1662**<br>SRE1658	Conventional H-bond<br>Conventional H-bond	**-4.5**	**LYS A45**	Conventional H-bond
6	3-oxo-tirucallic acid (β-Elemonic acid)	-5.2	GLN1695<br>ARG1651<br>TRP1620<br>**LYS1655**<br>ARG1749	Conventional H-bond<br>Alkyl<br>π –alkyl<br>Alkyl<br>Unfavorable donor-donor	-4.2	SER A65	Conventional H-bond
7	α-Amyrenone	-5.6		multiple Van der Waals forces	-4.7	PRO A51	Alkyl
8	Boscartin C	-3.9		multiple Van der Waals forces	**-5.0**	**LYS A45**<br>LYS A26	Conventional H-bond<br>Conventional H-bond
9	Boscartol G	-5.2	LYS1702	Alkyl	-6.0	LEU A49<br>ILE A62	Unfavorable donor-donor<br>Alkyl

Suppl. Table (4): Alignment of co-crystallized ligand with the docked ligand pose (yellow).

<table>
<tr><th>BCL-2</th><th>P53</th></tr>
<tr><td>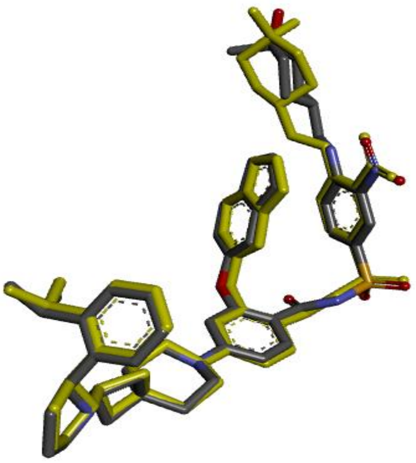</td><td>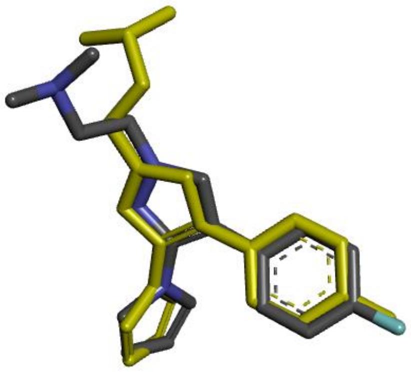</td></tr>
<tr><td>RMSD: 0.724</td><td>RMSD: 1.361</td></tr>
<tr><th>mTOR</th><th>LC3C</th></tr>
<tr><td>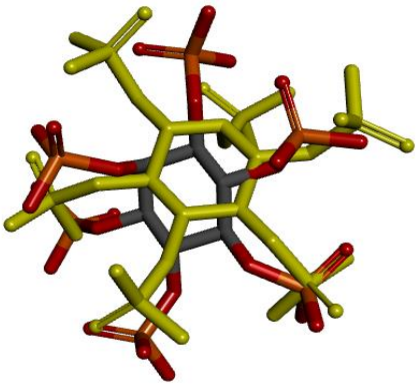</td><td>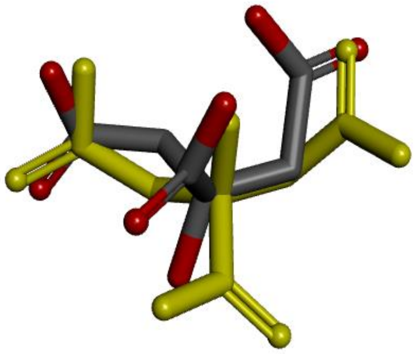</td></tr>
<tr><td>RMSD: 2.690</td><td>RMSD: 1.901</td></tr>
</table>

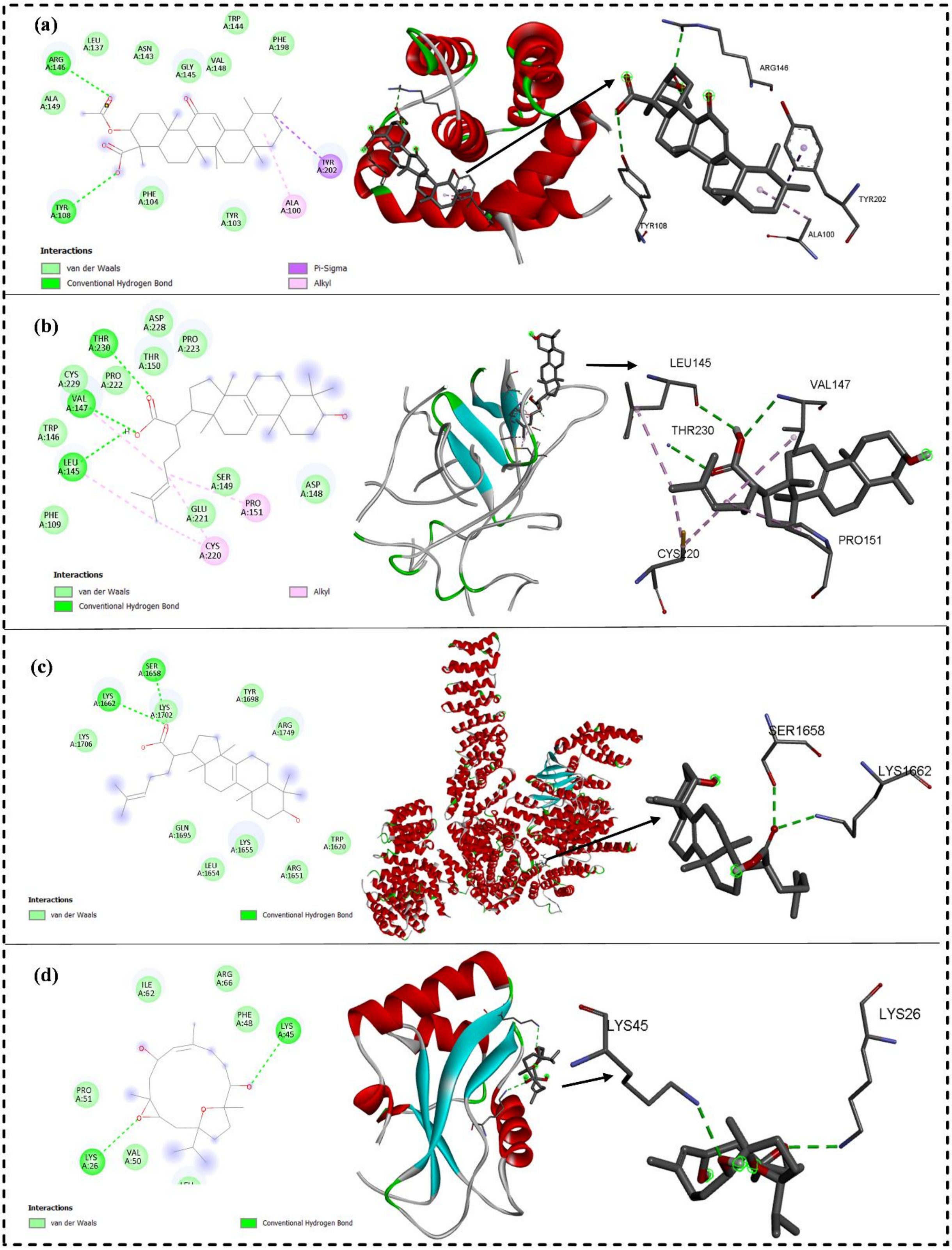

Suppl. Fig. 1. 2D and 3D pictures representing the Binding interactions of compounds with the highest binding energies: **(a)** 3-O-acetyl 11-keto boswellic acid in the active site of BCL-2, **(b)** α-Elemolic acid in the active site of P53, **(c)** α-Elemolic acid in the active site of mTOR, and **(d)** Boscartin C in the active site of LC3C.

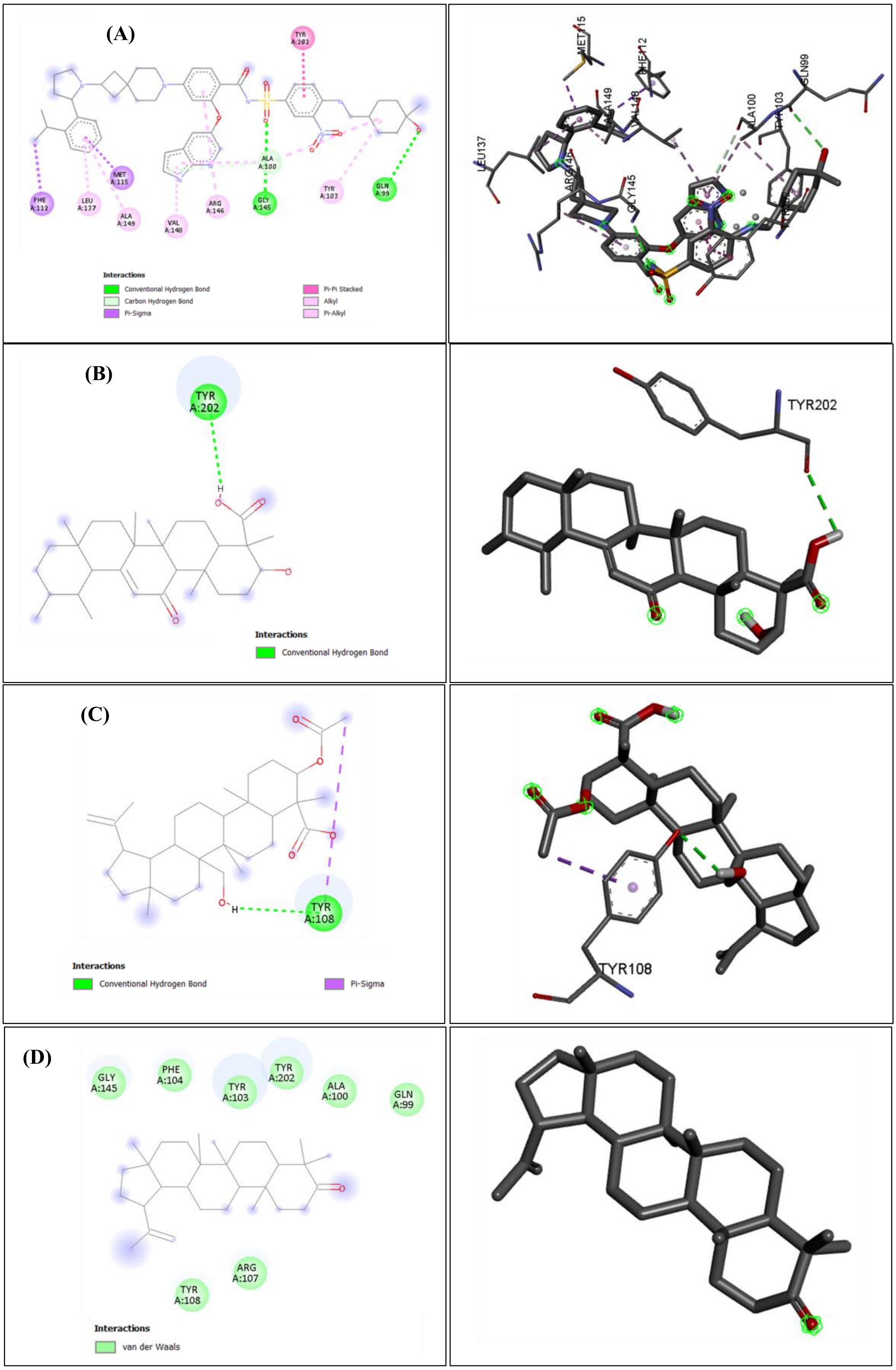

Suppl. Fig. 2. 2D and 3D pictures representing the Binding interactions of **(A)** Internal ligand, **(B)** 11-Keto boswellic acid, **(C)** 3-Acetyl-27-hydroxy-lupeolic acid, and **(D)** Lupenone in the active site of Bcl-2.

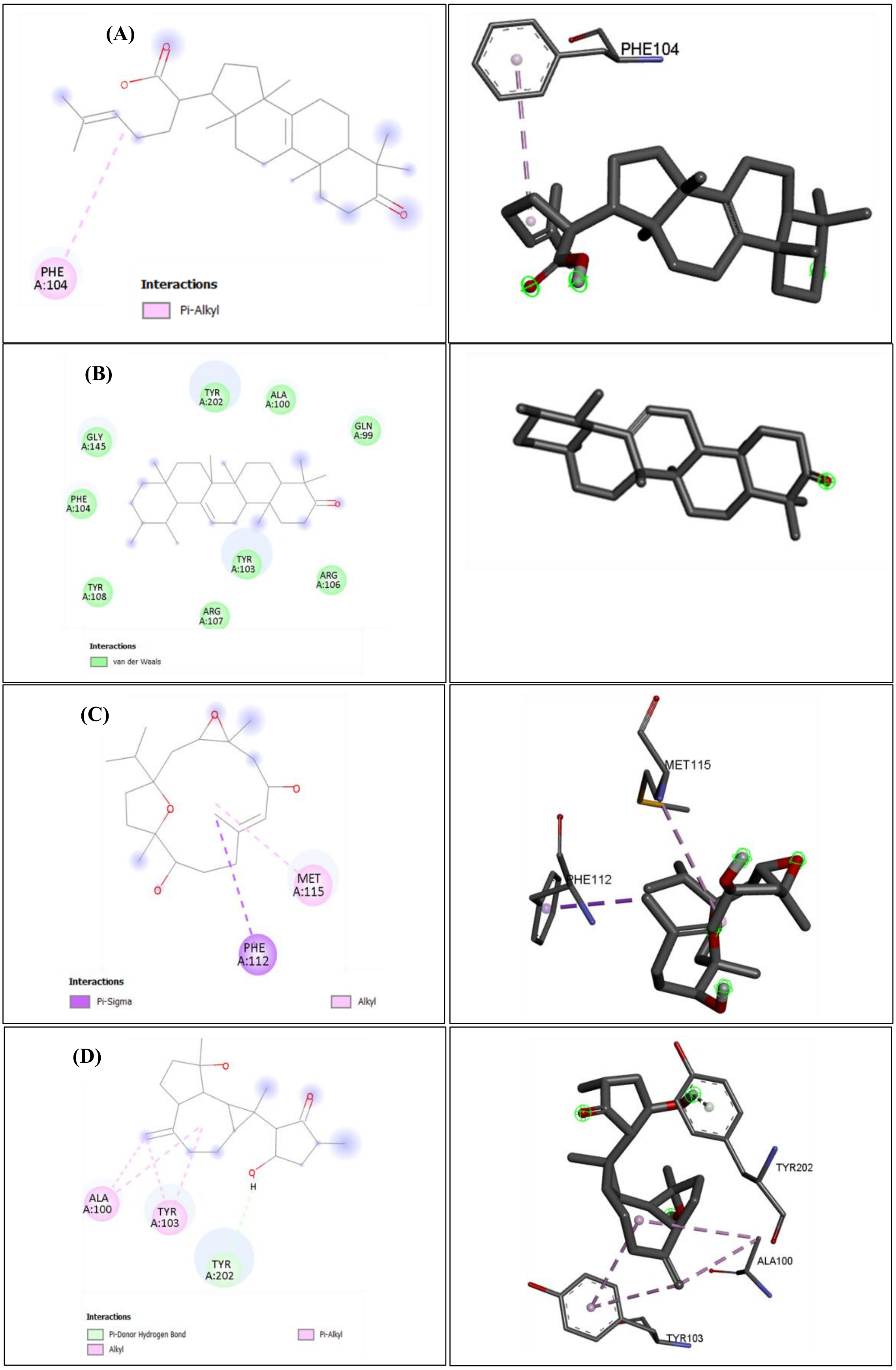

Suppl. Fig. 3. 2D and 3D pictures representing the Binding interactions of **(A)** 3-oxo-tirucallic acid, **(B)**, α-Amyrenone **(C)** Boscartin C, and **(D)** Boscartol G in the active site of Bcl-2.

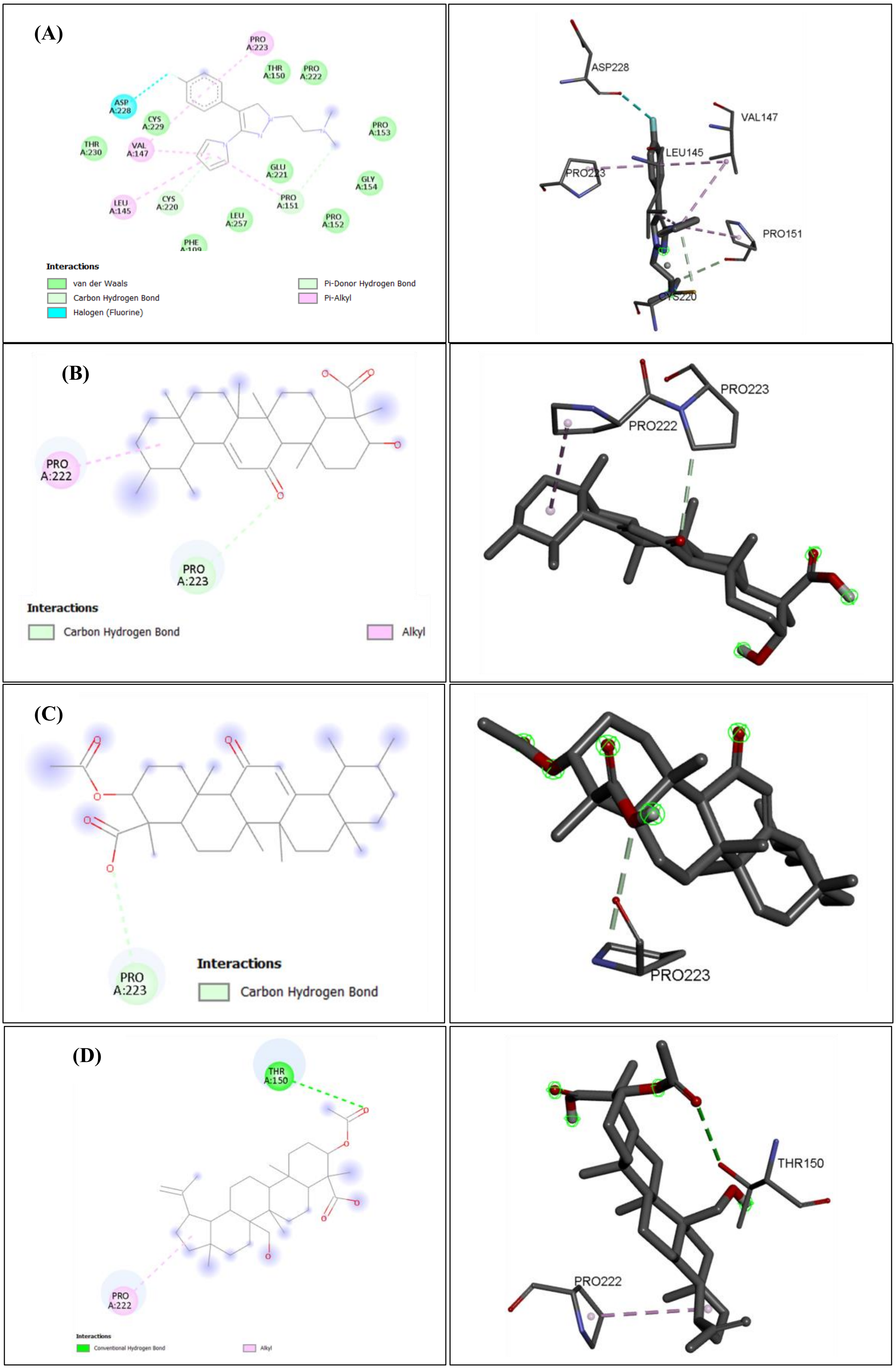

Suppl. Fig. 4. 2D and 3D pictures representing the Binding interactions of **(A)** Internal ligand, **(B)** 11-Keto boswellic acid, **(C)** 3-O-Acetyl 11-keto boswellic acid, and **(D)** 3-Acetyl-27-hydroxy-lupeolic acid in the active site of P53.

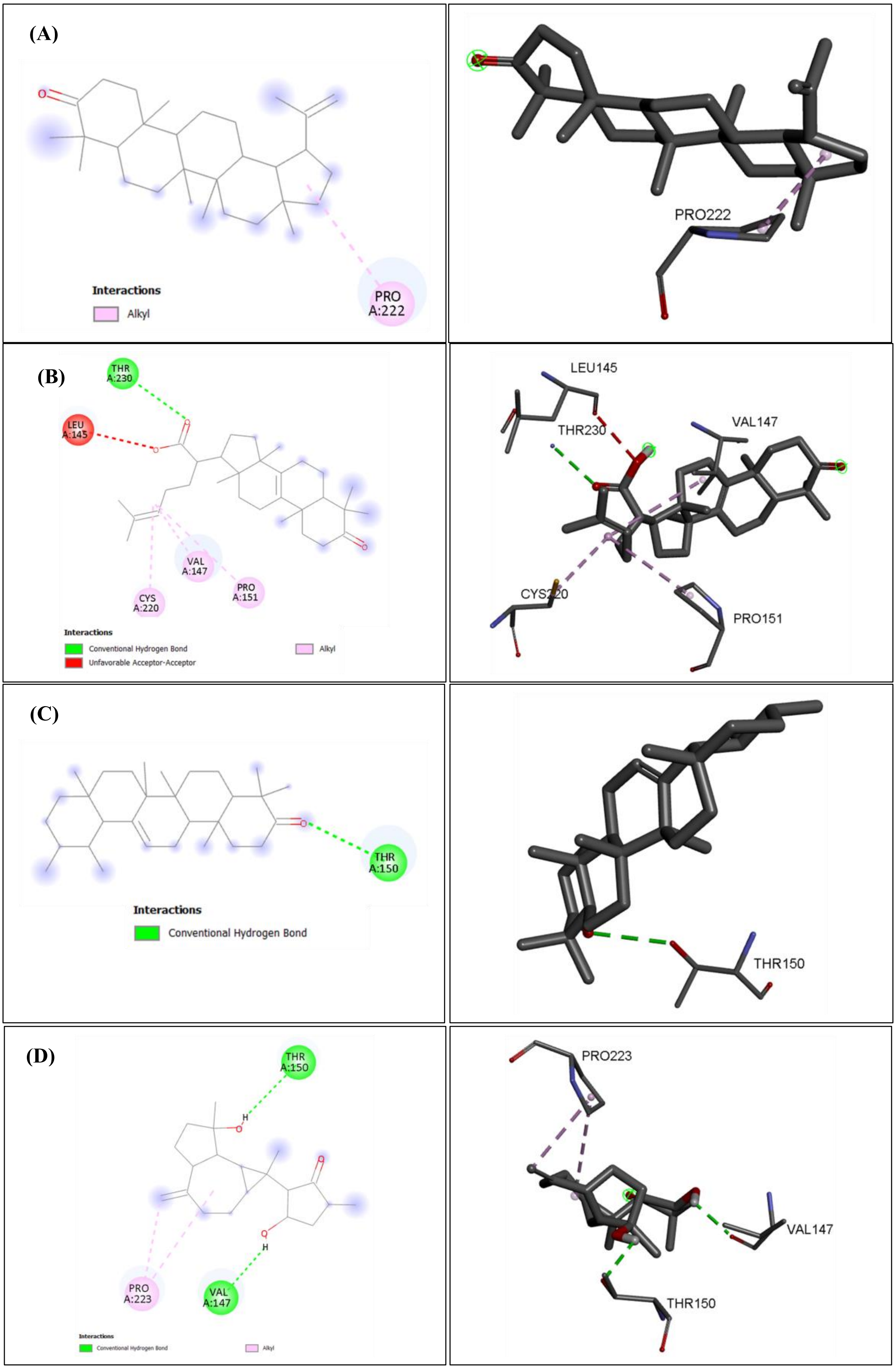

Suppl. Fig. 5. 2D and 3D pictures representing the Binding interactions of **(A)** Lupenone, **(B)** 3-oxo-tirucallic acid, **(C)** α-Amyrenone, and **(D)** Boscartol G in the active site of P53.

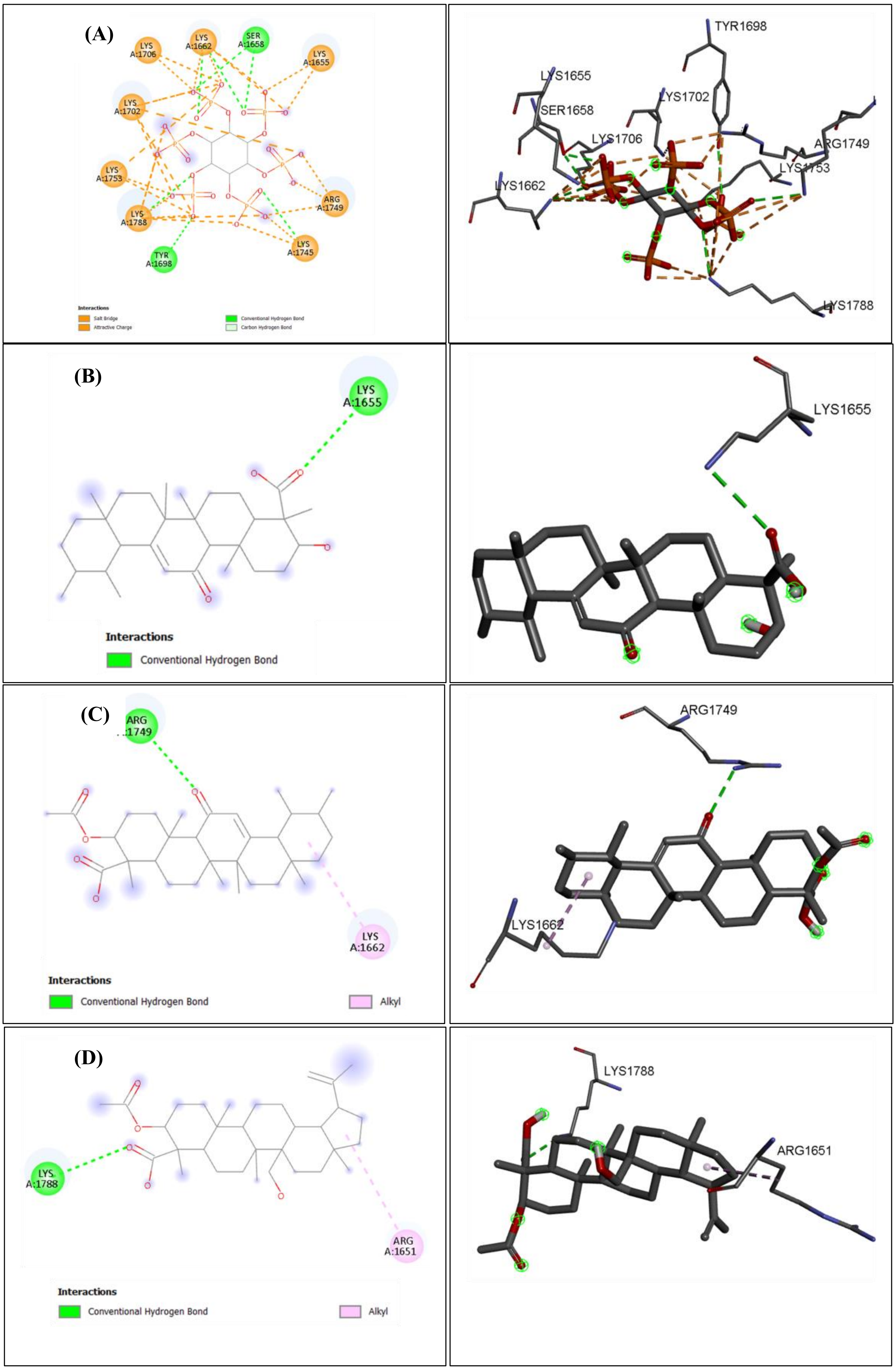

Suppl. Fig. 6. 2D and 3D pictures representing the Binding interactions of **(A)** Internal ligand, **(B)** 11-Keto boswellic acid, **(C)** 3-O-Acetyl 11-keto boswellic acid, and **(D)** 3-Acetyl-27-hydroxy-lupeolic acid in the active site of mTOR.

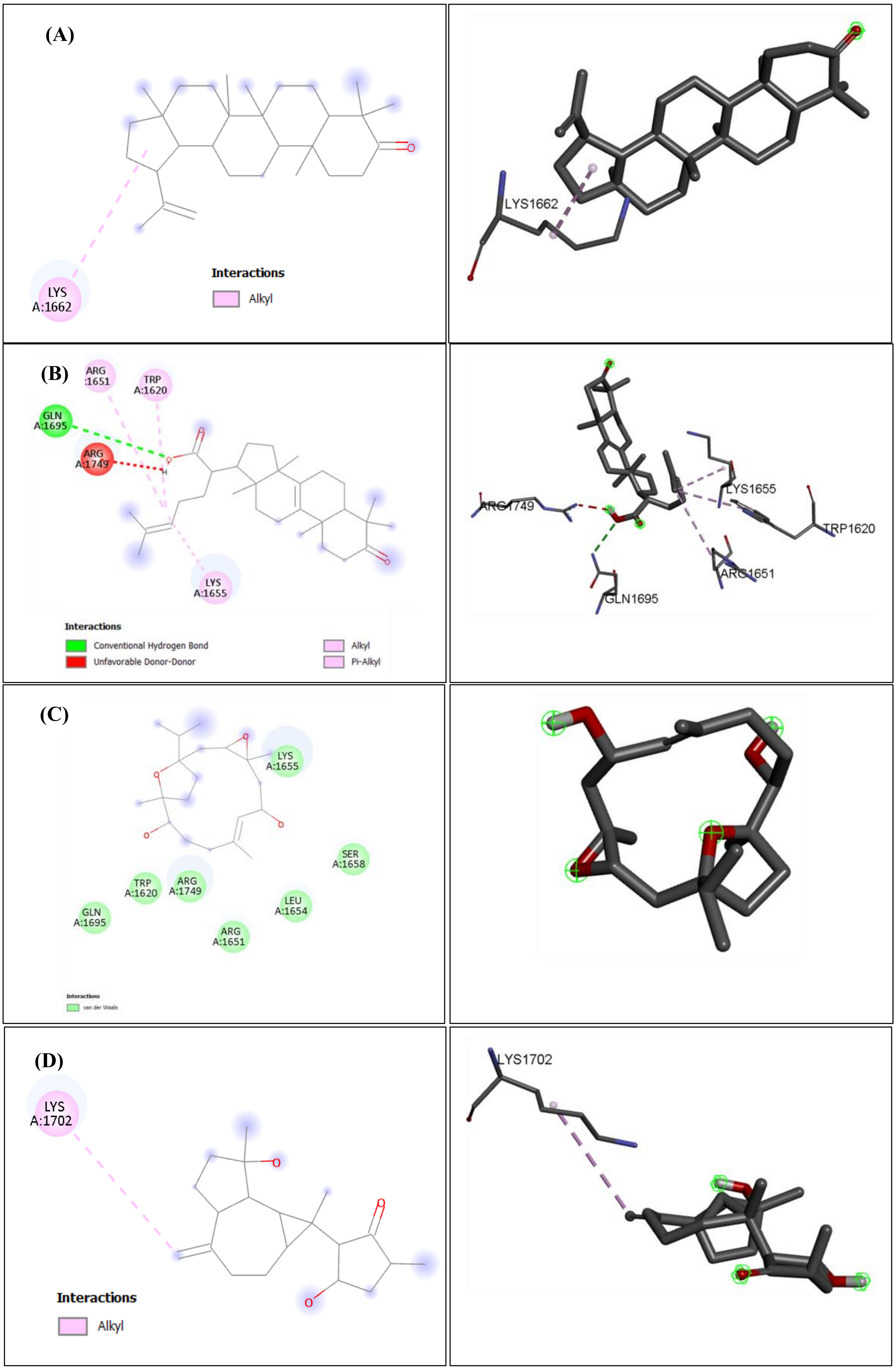

Suppl. Fig. 7. 2D and 3D pictures representing the Binding interactions of **(A)** Lupenone, **(B)** 3-oxo-tirucallic acid, **(C)** Boscartin C, and **(D)** Boscartol G in the active site of mTOR.

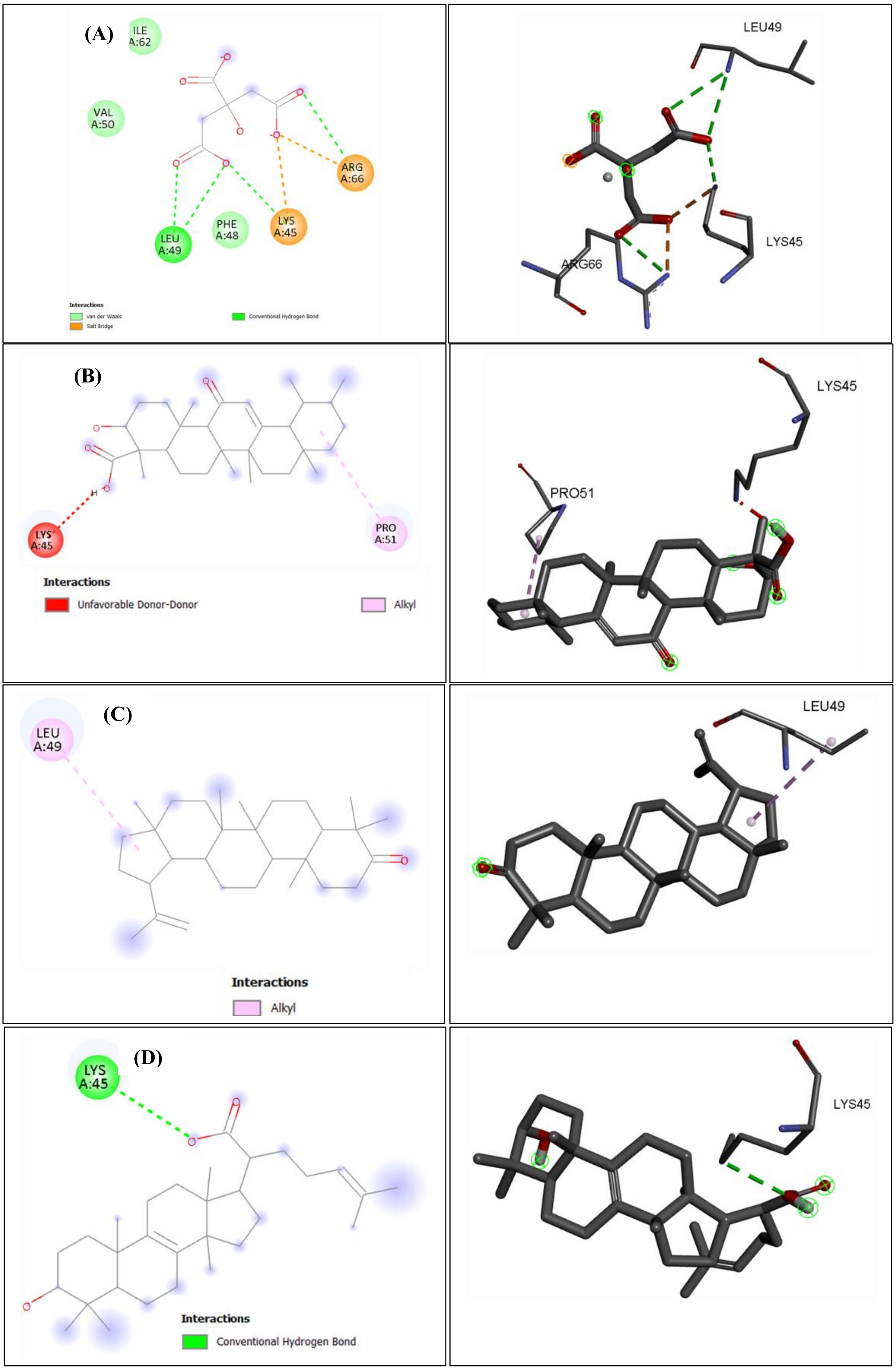

Suppl. Fig. 8. 2D and 3D pictures representing the Binding interactions of **(A)** Internal ligand, **(B)** 11-Keto boswellic acid, **(C)** Lupenone, and **(D)** 3-hydroxytirucallic acid in the active site of LC3C.

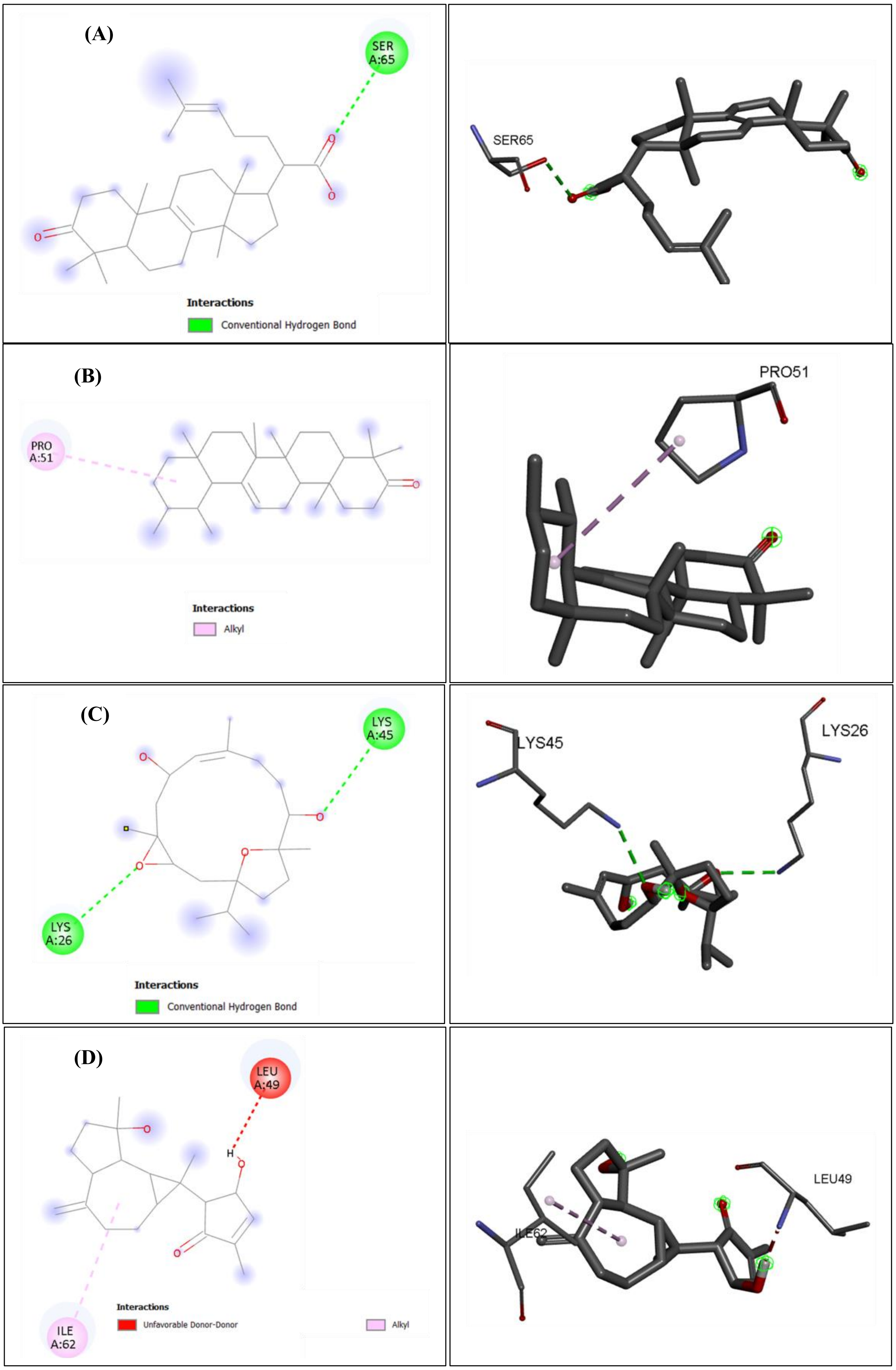

Suppl. Fig. 9. 2D and 3D pictures representing the Binding interactions of **(A)** 3-oxo-tirucallic acid, **(B)** α-Amyrenone, **(C)** Boscartin C, and **(D)** Boscartol G in the active site of LC3C.

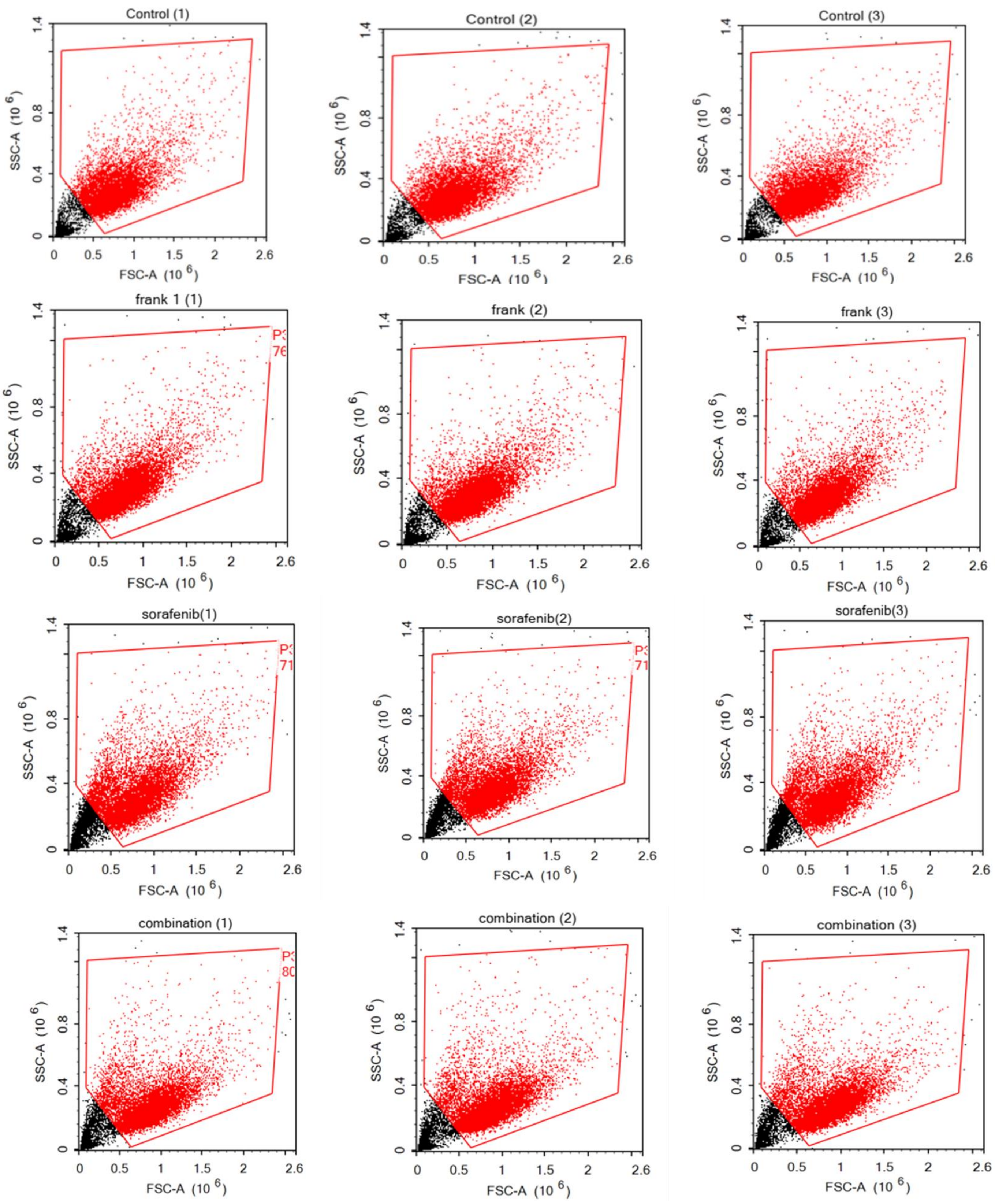

Suppl. Fig. 10. The raw apoptosis/necrosis assay data for Figure 12 demonstrate consistent gating across all tested experimental groups, for clarification and reliable verification of the results.

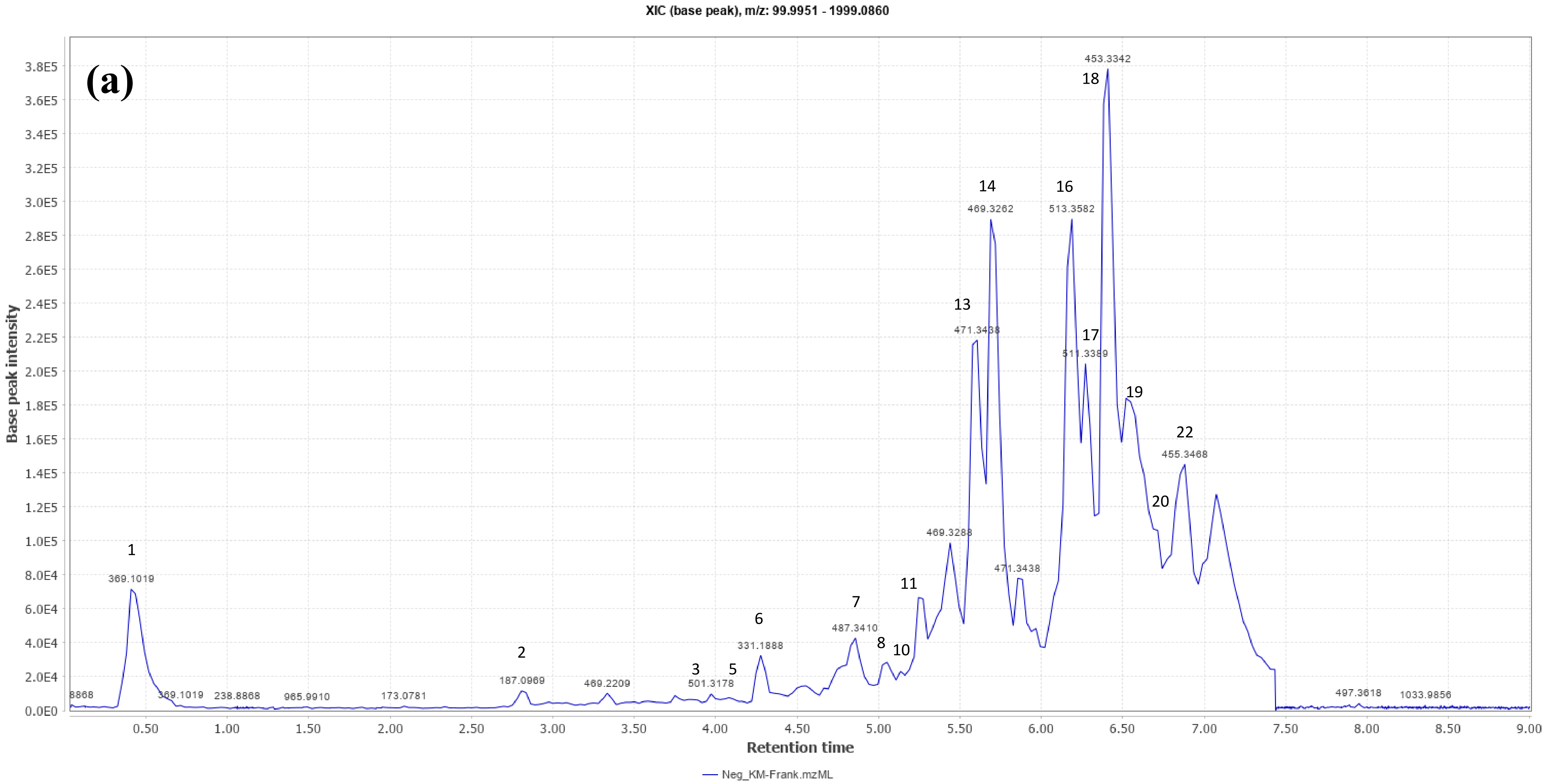

XIC (base peak), m/z: 99.9951 - 1999.0860
(a)
Base peak intensity
Retention time
Neg_KM-Frank.mzML
1
369.1019
2
187.0969
3
501.3178
5
6
331.1888
7
487.3410
8
10
11
469.3288
13
471.3438
14
469.3262
471.3438
16
513.3582
17
511.3389
18
453.3342
19
20
22
455.3468
497.3618
1033.9856

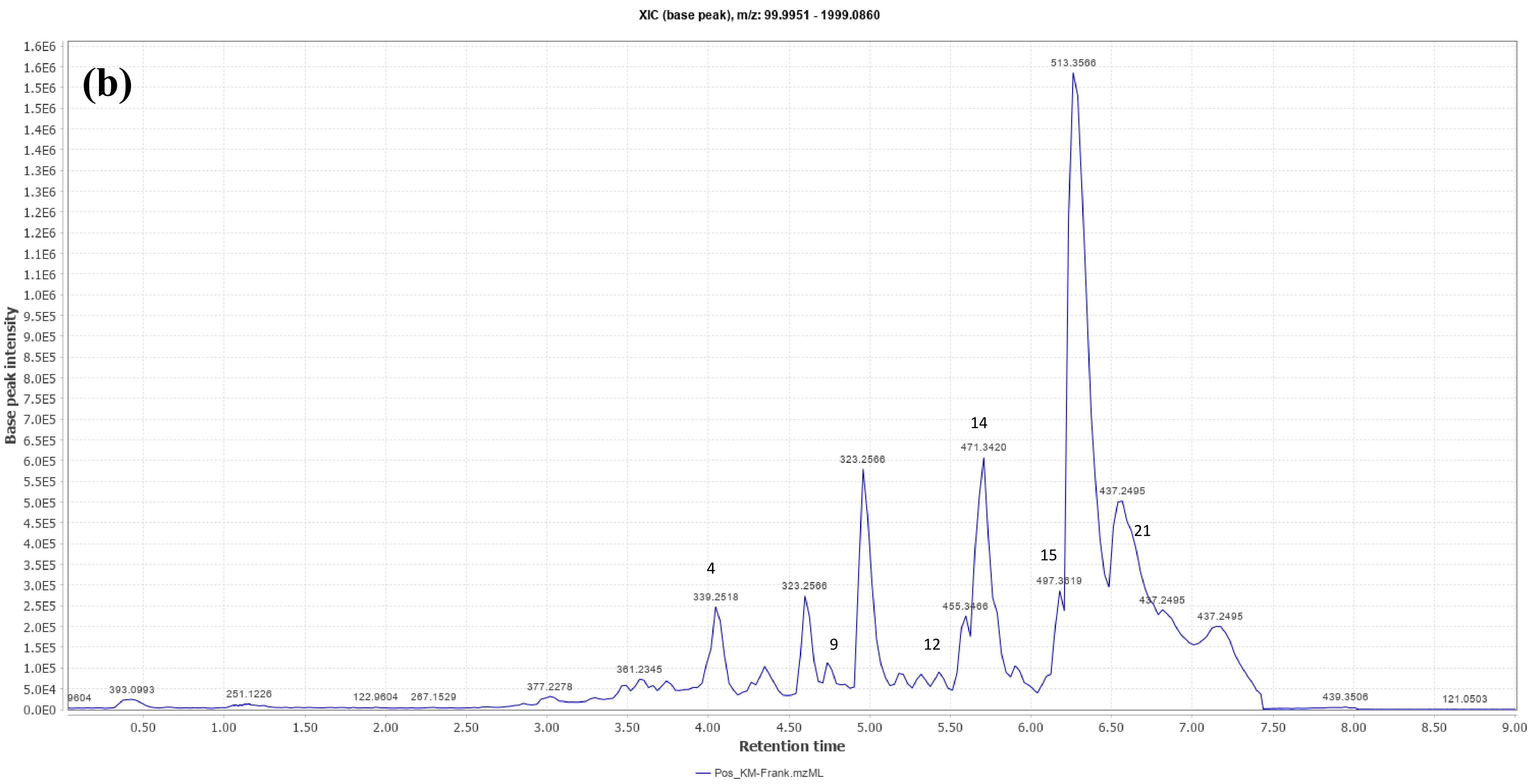
XIC (base peak), m/z: 99.9951 - 1999.0860
(b)
Base peak intensity
Retention time
Pos_KM-Frank.mzML
513.3566
471.3420
323.2566
437.2495
497.3619
455.3466
339.2518
361.2345
377.2278
393.0993
251.1226
122.9604
267.1529
439.3506
121.0503
4
9
12
14
15
21

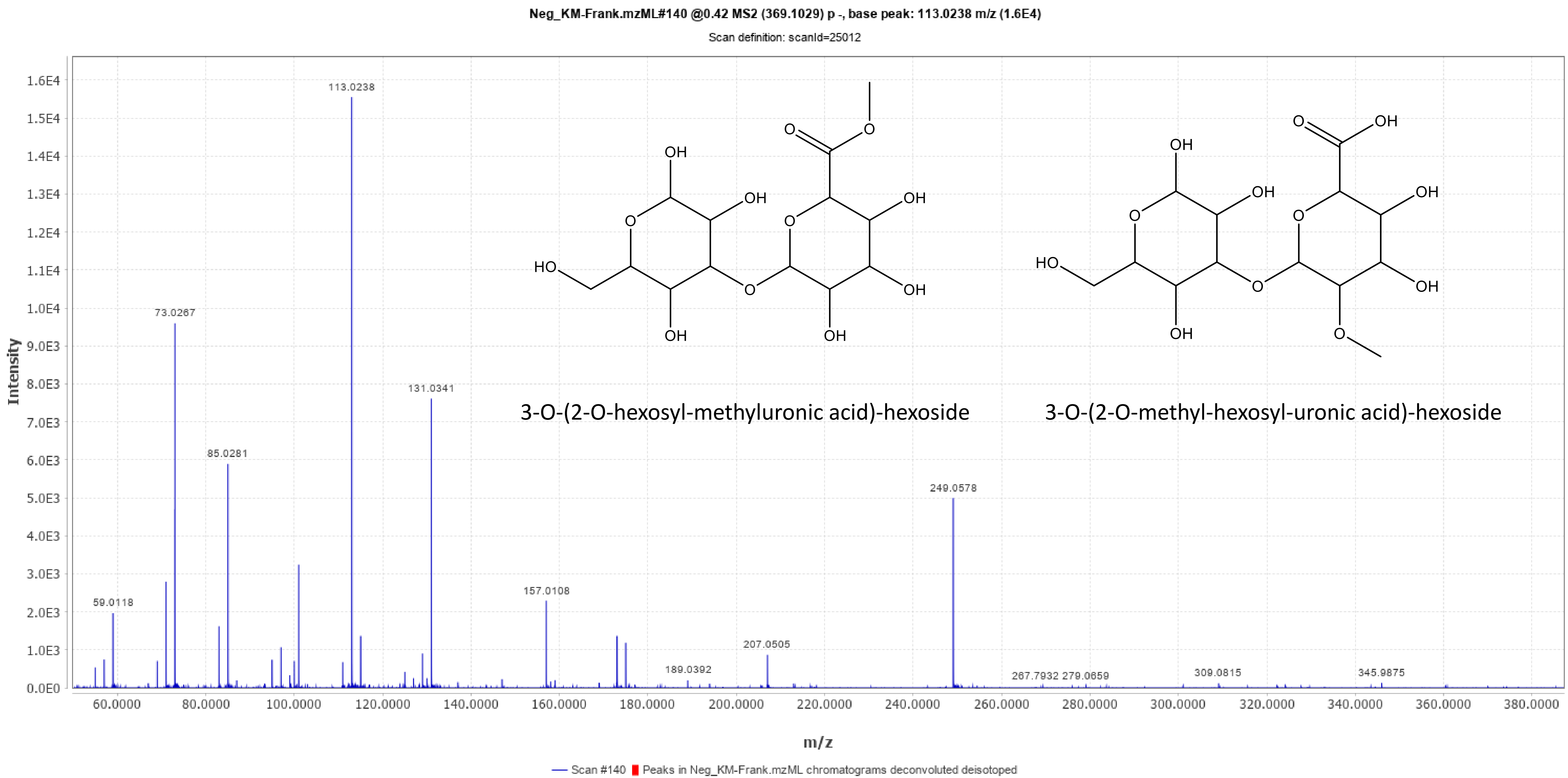
Neg_KM-Frank.mzML#140 @0.42 MS2 (369.1029) p -, base peak: 113.0238 m/z (1.6E4)
Scan definition: scanId=25012
Intensity
m/z
113.0238
73.0267
85.0281
131.0341
59.0118
157.0108
249.0578
207.0505
189.0392
267.7932 279.0659
309.0815
345.9875
3-O-(2-O-hexosyl-methyluronic acid)-hexoside
3-O-(2-O-methyl-hexosyl-uronic acid)-hexoside
Scan #140
Peaks in Neg_KM-Frank.mzML chromatograms deconvoluted deisotoped

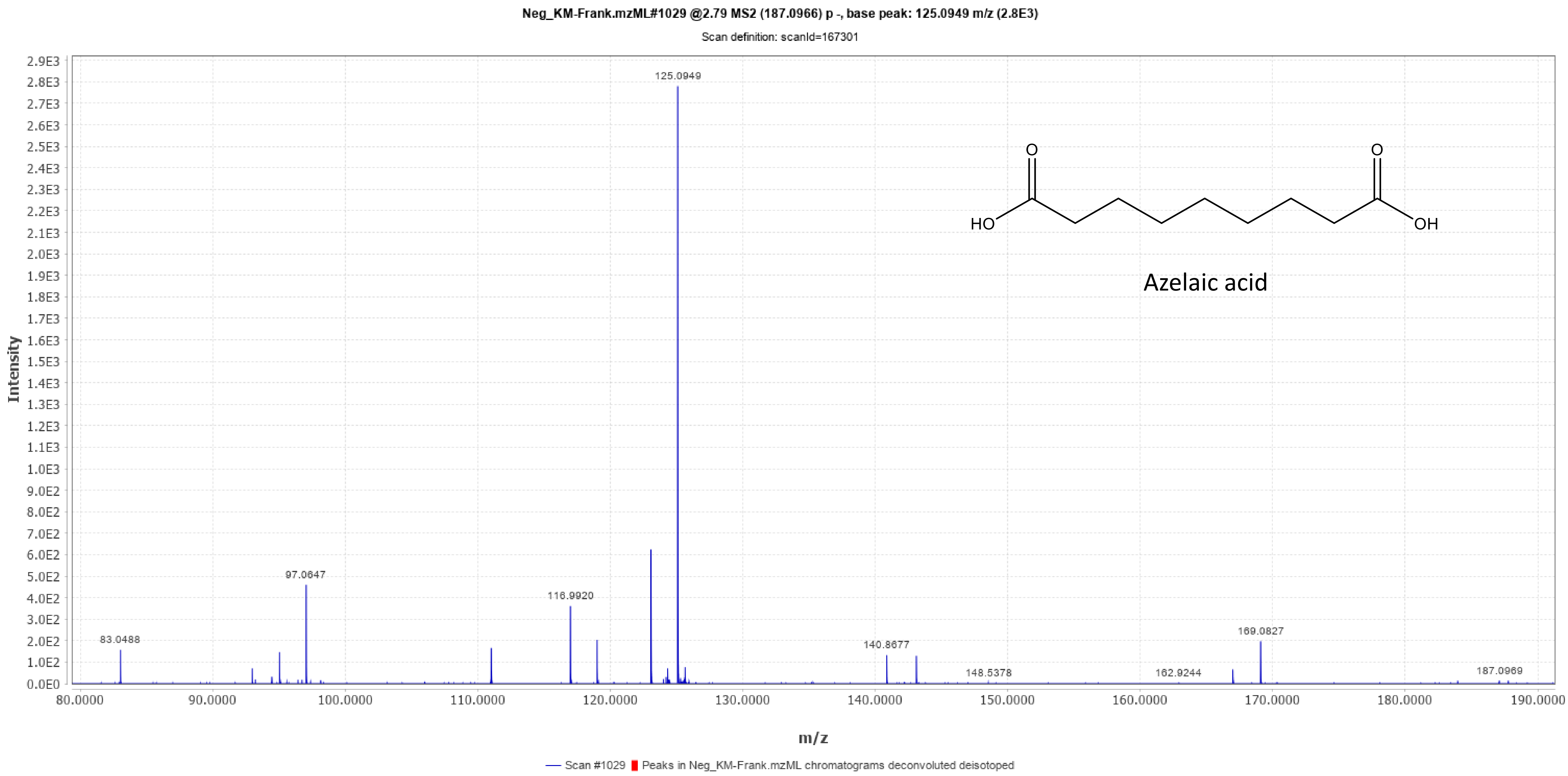
Neg_KM-Frank.mzML#1029 @2.79 MS2 (187.0966) p -, base peak: 125.0949 m/z (2.8E3)
Scan definition: scanId=167301
Intensity
m/z
125.0949
97.0647
116.9920
83.0488
140.8677
148.5378
162.9244
169.0827
187.0969
HO
OH
O
O
Azelaic acid
Scan #1029
Peaks in Neg_KM-Frank.mzML chromatograms deconvoluted deisotoped

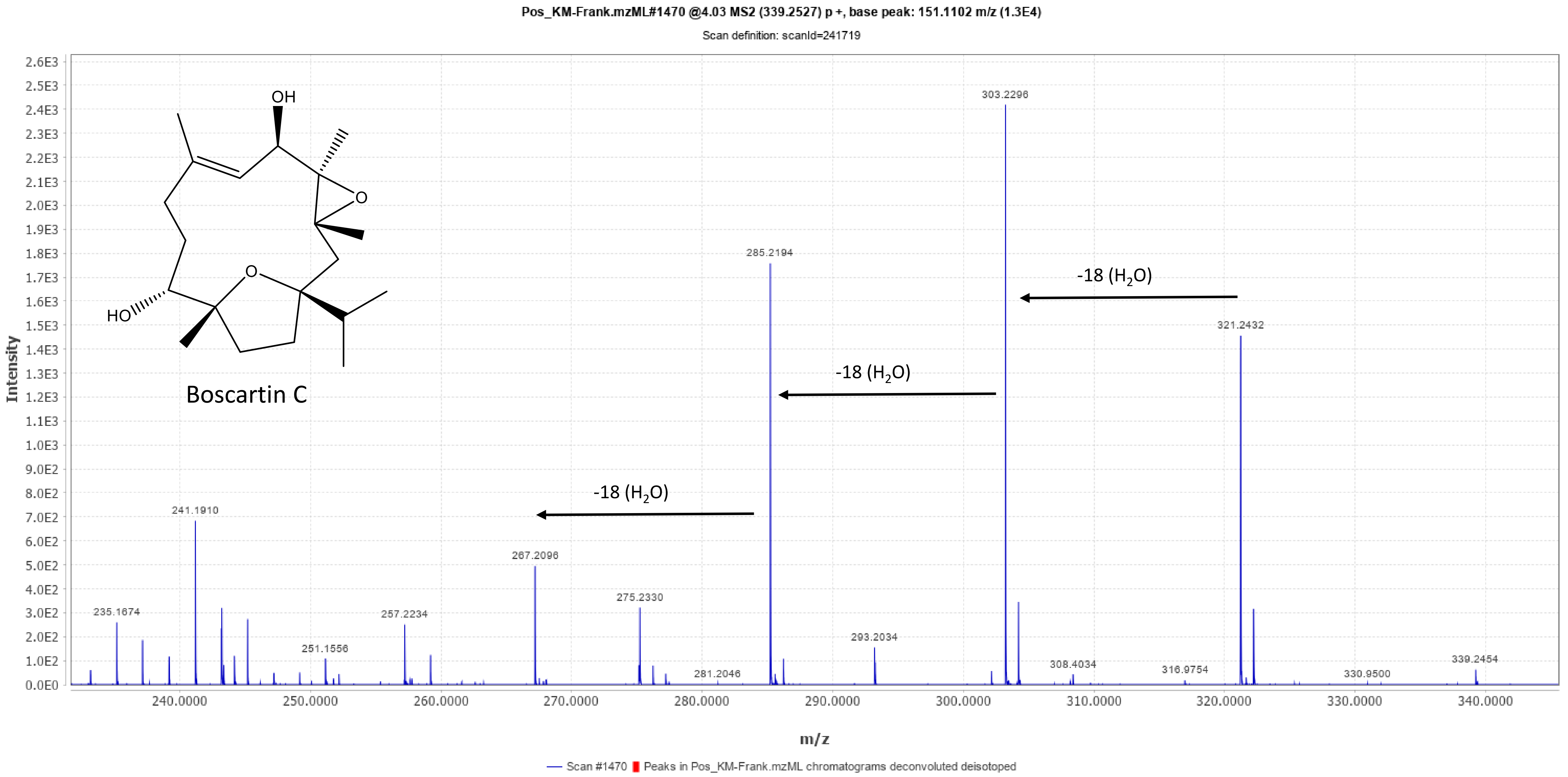

Pos_KM-Frank.mzML#1470 @4.03 MS2 (339.2527) p +, base peak: 151.1102 m/z (1.3E4)
Scan definition: scanId=241719
Intensity
m/z
Boscartin C
OH
O
O
HO
-18 (H_2O)
-18 (H_2O)
-18 (H_2O)
303.2296
285.2194
321.2432
241.1910
267.2096
275.2330
235.1674
257.2234
251.1556
293.2034
281.2046
308.4034
316.9754
330.9500
339.2454
Scan #1470
Peaks in Pos_KM-Frank.mzML chromatograms deconvoluted deisotoped

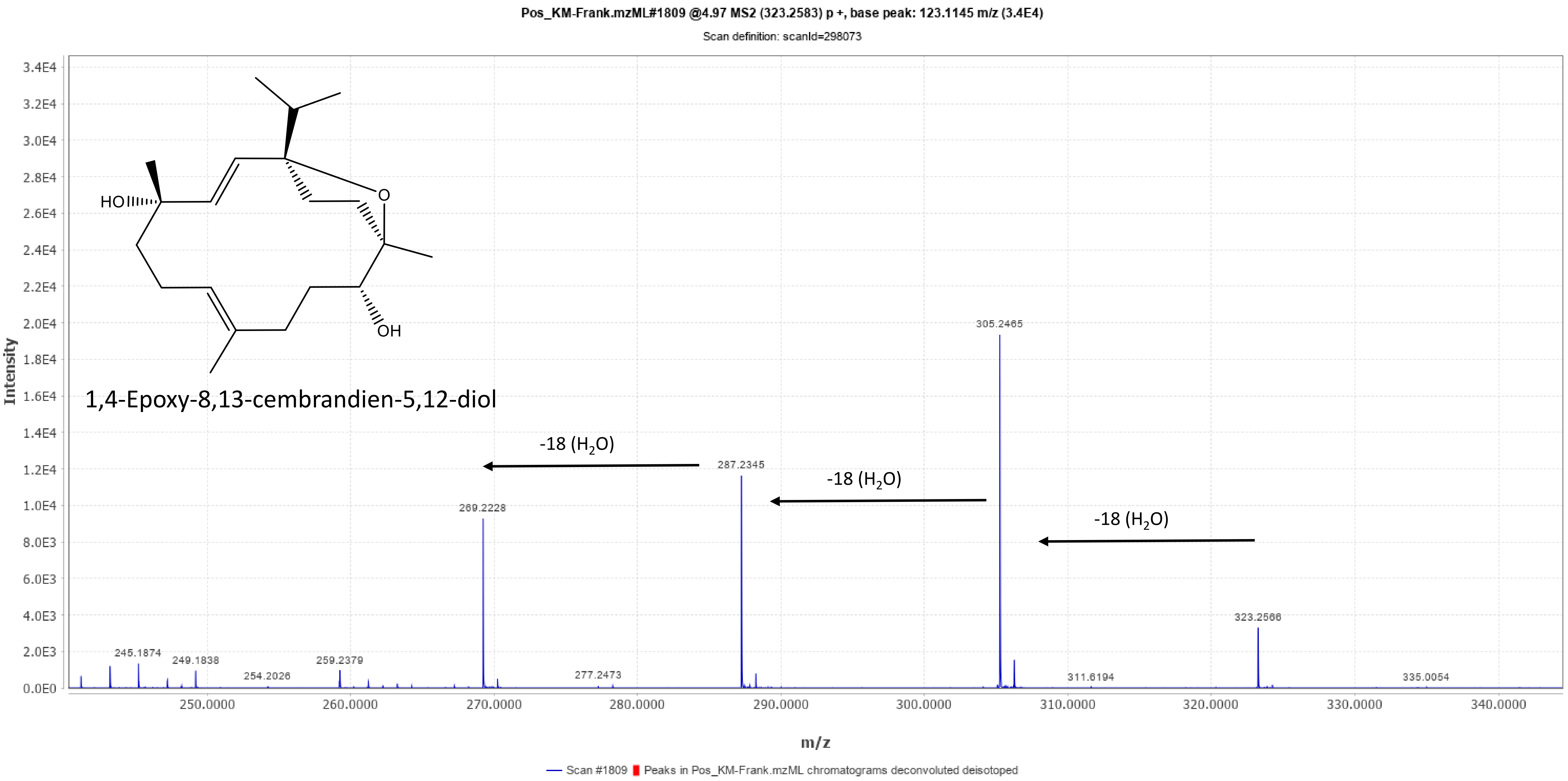
Pos_KM-Frank.mzML#1809 @4.97 MS2 (323.2583) p +, base peak: 123.1145 m/z (3.4E4)
Scan definition: scanId=298073
HO
O
OH
1,4-Epoxy-8,13-cembrandien-5,12-diol
-18 (H_2O)
-18 (H_2O)
-18 (H_2O)
305.2465
287.2345
269.2228
323.2566
245.1874
249.1838
254.2026
259.2379
277.2473
311.6194
335.0054
Intensity
m/z
Scan #1809 Peaks in Pos_KM-Frank.mzML chromatograms deconvoluted deisotoped

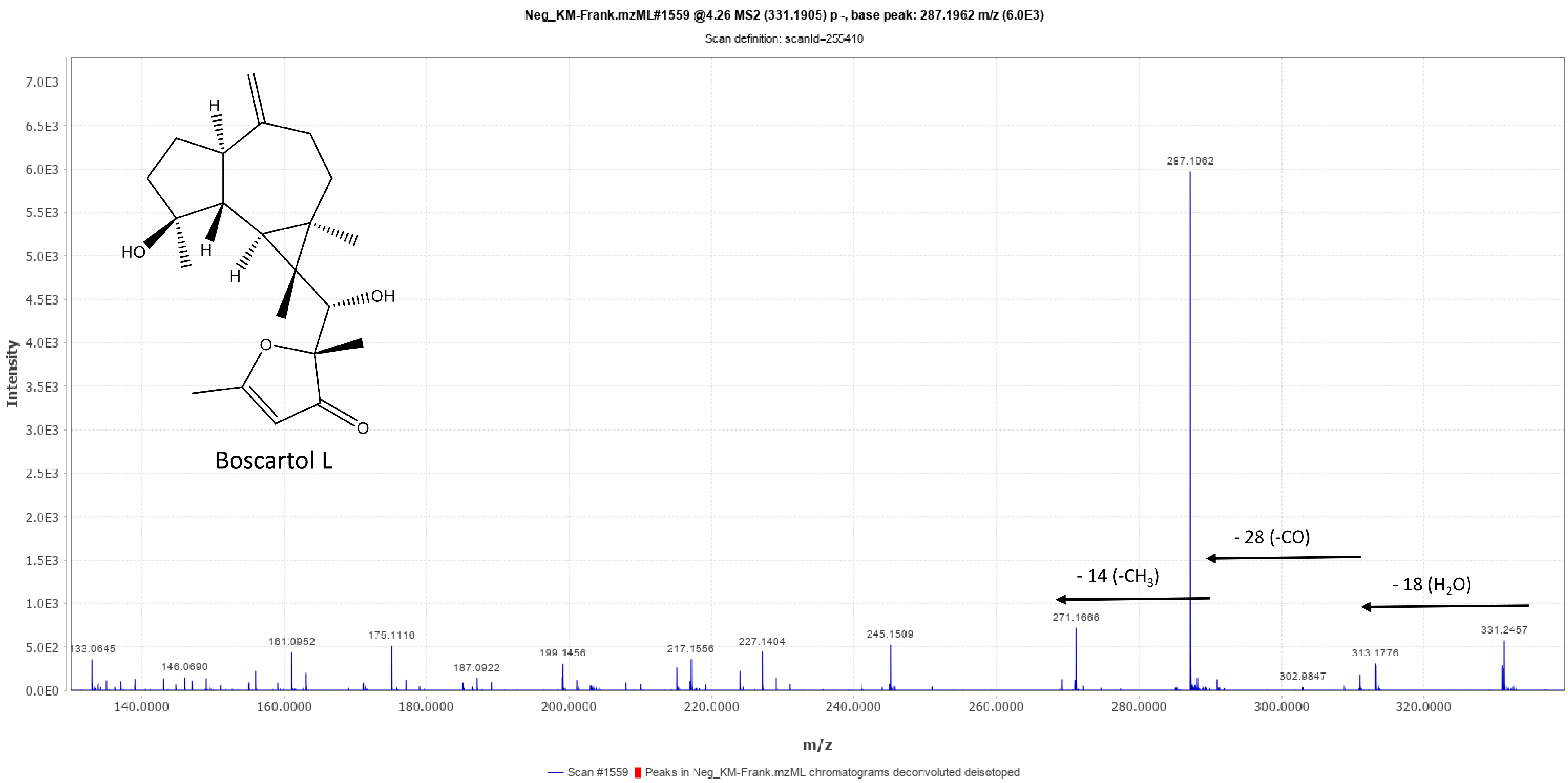

Neg_KM-Frank.mzML#1559 @4.26 MS2 (331.1905) p -, base peak: 287.1962 m/z (6.0E3)
Scan definition: scanId=255410
Intensity
m/z
Boscartol L
HO
H
OH
O
287.1962
271.1666
331.2457
313.1776
302.9847
245.1509
227.1404
217.1556
199.1456
187.0922
175.1116
161.0952
146.0690
133.0645
- 28 (-CO)
- 14 (-CH3)
- 18 (H2O)
Scan #1559
Peaks in Neg_KM-Frank.mzML chromatograms deconvoluted deisotoped

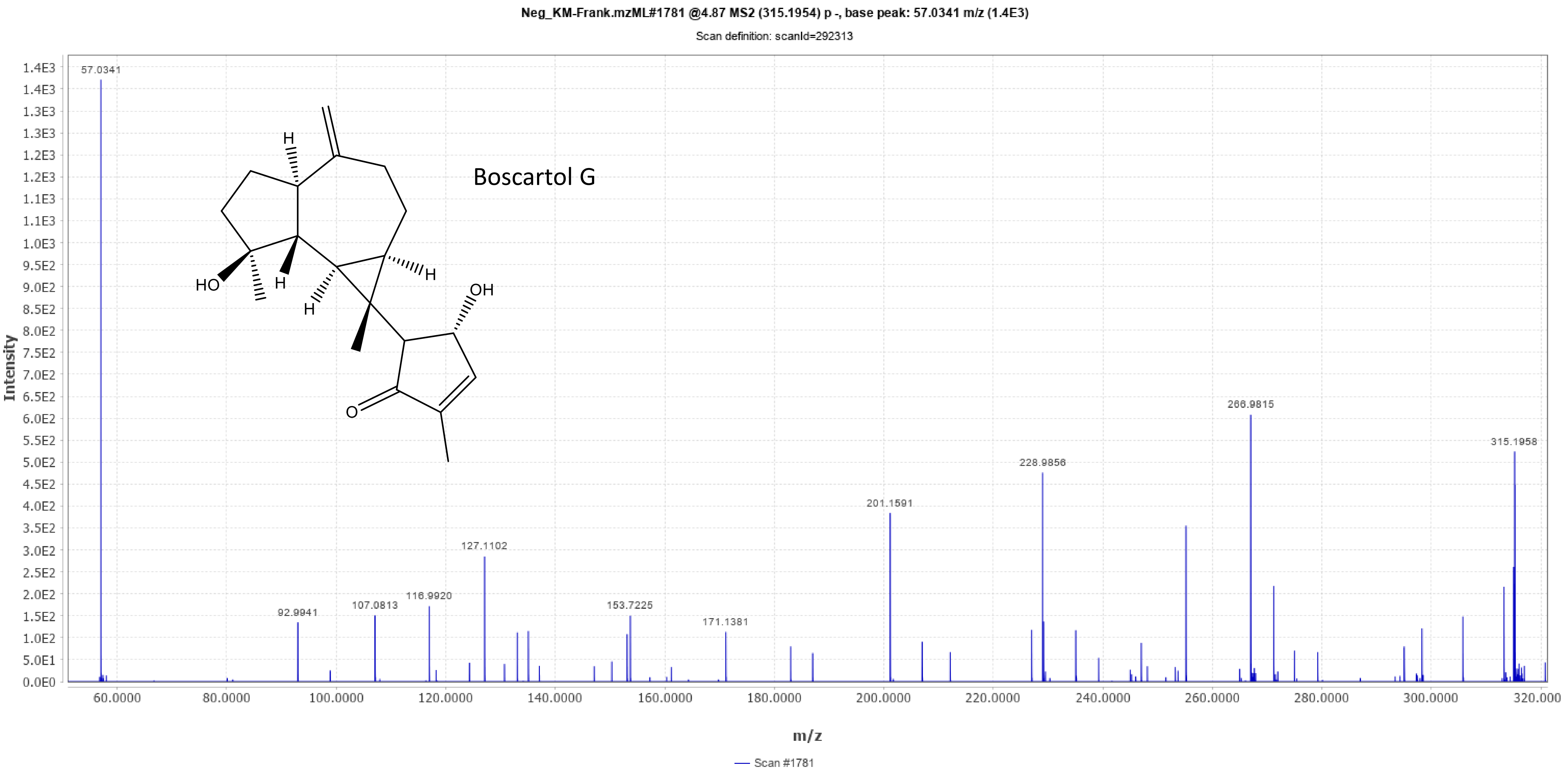
Neg_KM-Frank.mzML#1781 @4.87 MS2 (315.1954) p -, base peak: 57.0341 m/z (1.4E3)
Scan definition: scanId=292313
Intensity
m/z
57.0341
92.9941
107.0813
116.9920
127.1102
153.7225
171.1381
201.1591
228.9856
266.9815
315.1958
Boscartol G
HO
OH
H
O
Scan #1781

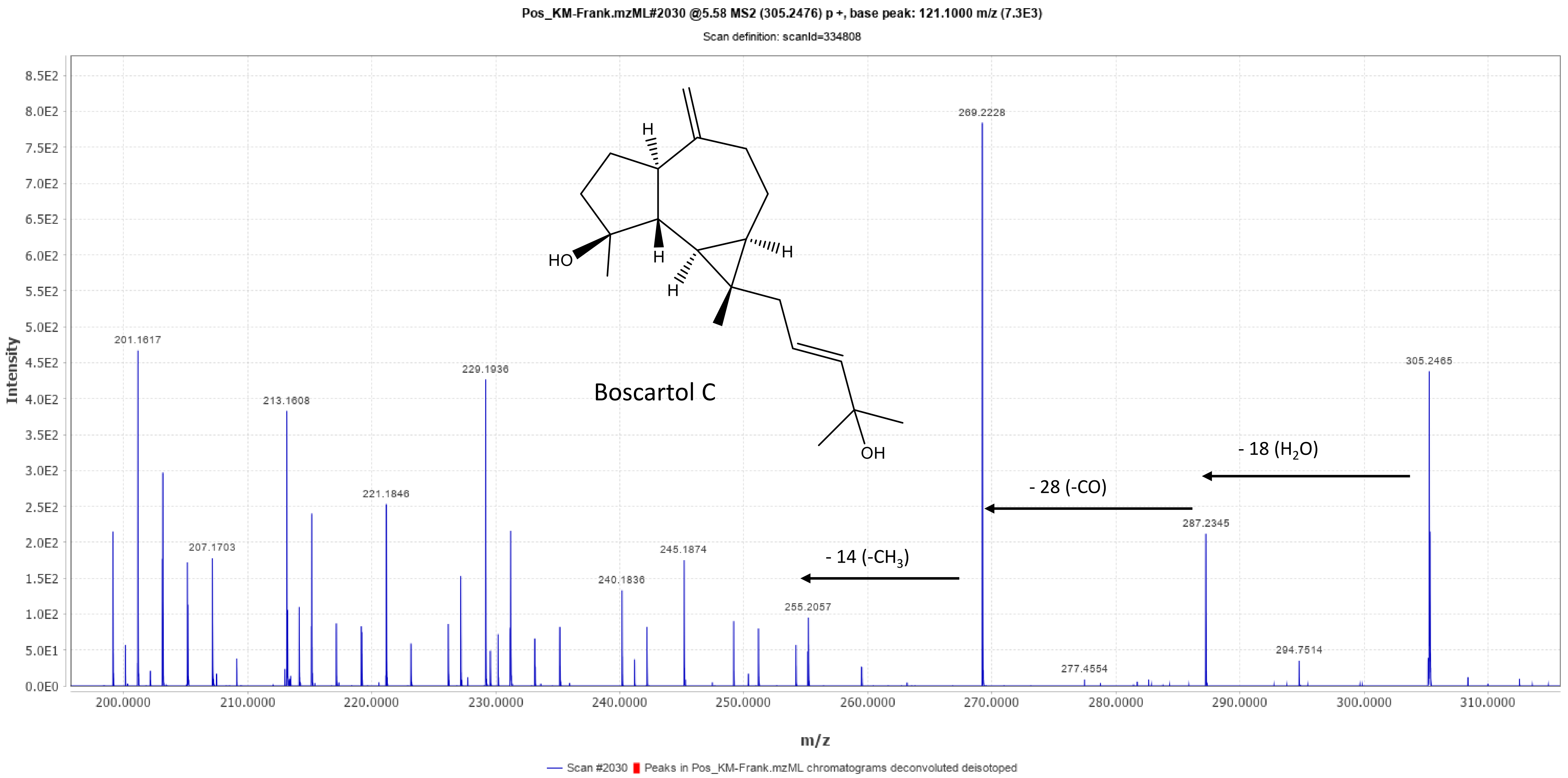

Pos_KM-Frank.mzML#2030 @5.58 MS2 (305.2476) p +, base peak: 121.1000 m/z (7.3E3)
Scan definition: scanId=334808
Intensity
m/z
201.1617
207.1703
213.1608
221.1846
229.1936
240.1836
245.1874
255.2057
269.2228
277.4554
287.2345
294.7514
305.2465
- 18 (H_2O)
- 28 (-CO)
- 14 (-CH_3)
Boscartol C
HO
OH
H
Scan #2030
Peaks in Pos_KM-Frank.mzML chromatograms deconvoluted deisotoped

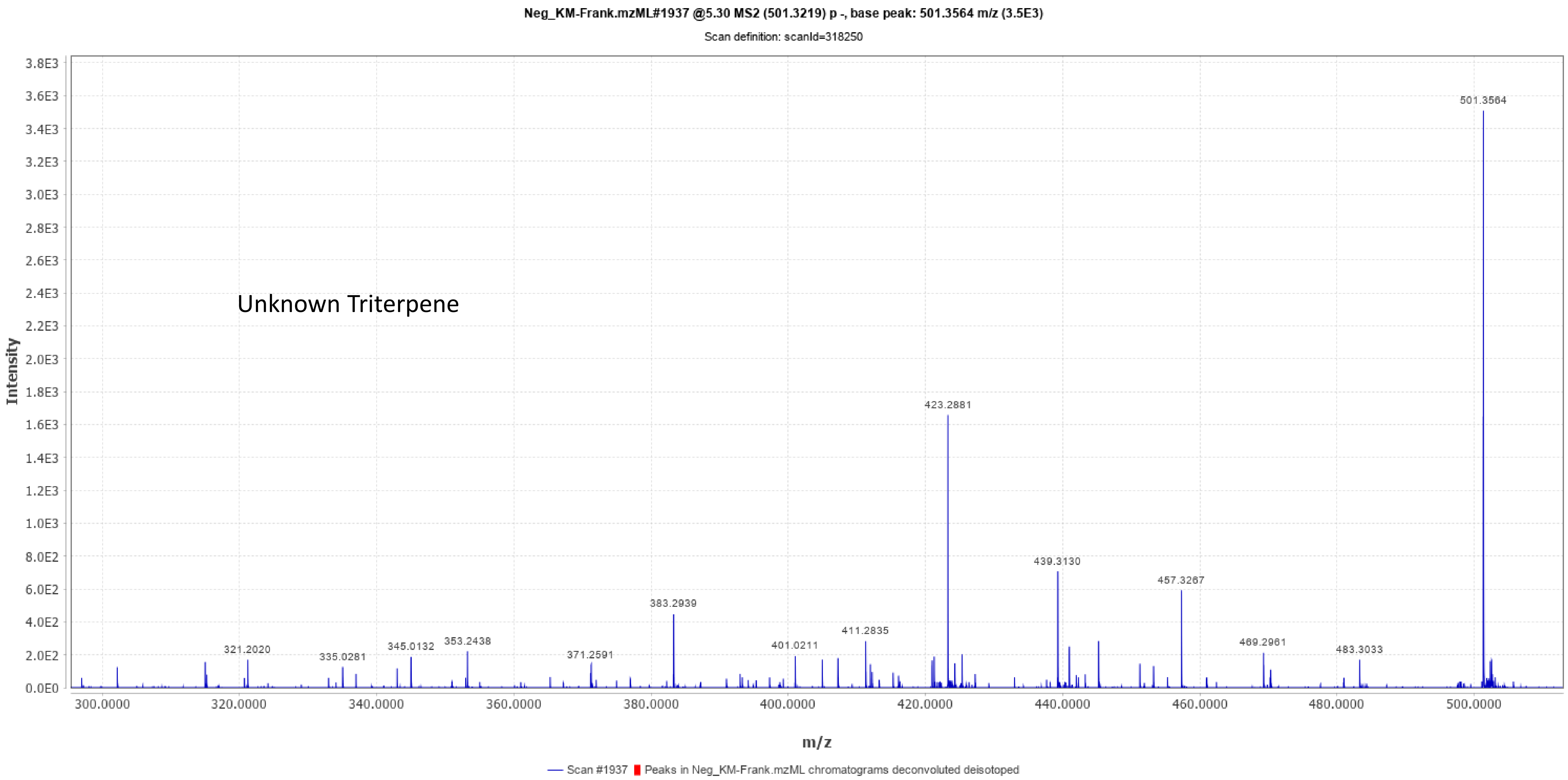

Neg_KM-Frank.mzML#1937 @5.30 MS2 (501.3219) p -, base peak: 501.3564 m/z (3.5E3)
Scan definition: scanId=318250
Unknown Triterpene
Intensity
m/z
501.3564
423.2881
439.3130
457.3267
383.2939
411.2835
469.2961
483.3033
401.0211
353.2438
345.0132
321.2020
335.0281
371.2591
Scan #1937
Peaks in Neg_KM-Frank.mzML chromatograms deconvoluted deisotoped

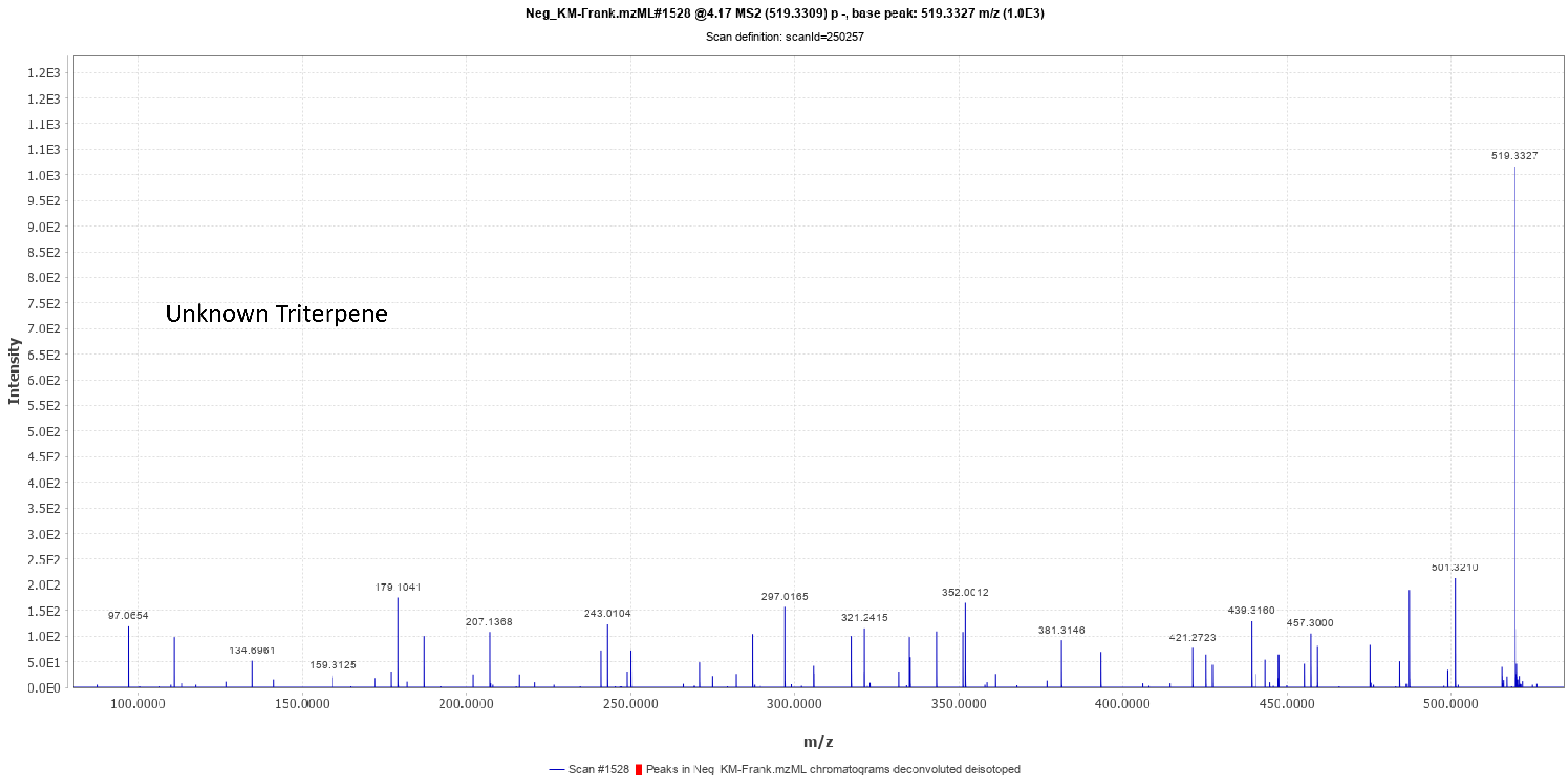
Neg_KM-Frank.mzML#1528 @4.17 MS2 (519.3309) p -, base peak: 519.3327 m/z (1.0E3)
Scan definition: scanId=250257
Unknown Triterpene
Intensity
m/z
519.3327
501.3210
97.0654
134.6961
159.3125
179.1041
207.1368
243.0104
297.0165
321.2415
352.0012
381.3146
421.2723
439.3160
457.3000
Scan #1528
Peaks in Neg_KM-Frank.mzML chromatograms deconvoluted deisotoped

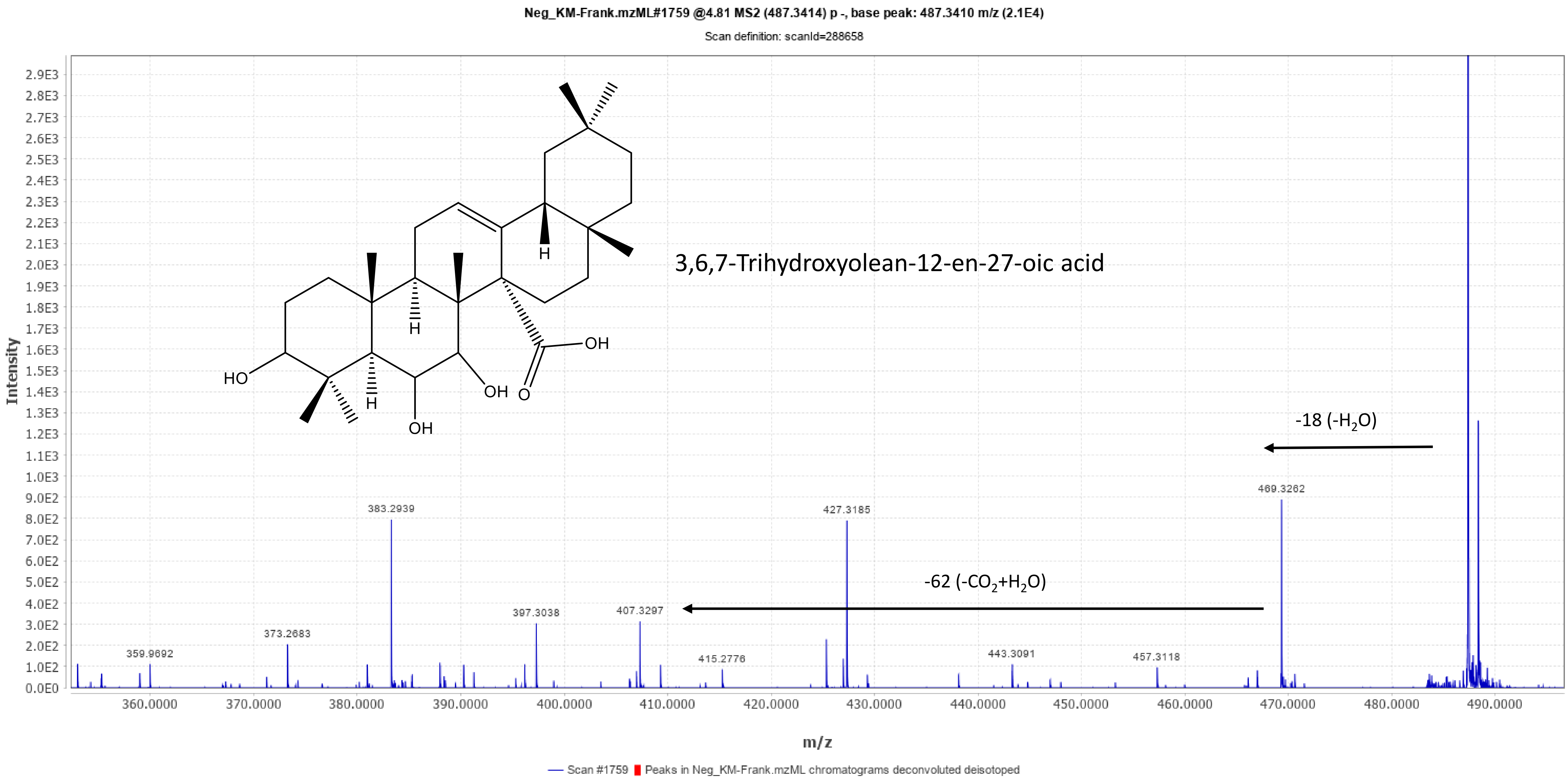

Neg_KM-Frank.mzML#1759 @4.81 MS2 (487.3414) p -, base peak: 487.3410 m/z (2.1E4)
Scan definition: scanId=288658
3,6,7-Trihydroxyolean-12-en-27-oic acid
HO
OH
OH
OH
O
H
H
H
-18 (-H2O)
-62 (-CO2+H2O)
469.3262
427.3185
383.2939
397.3038
407.3297
373.2683
359.9692
415.2776
443.3091
457.3118
Intensity
m/z
Scan #1759
Peaks in Neg_KM-Frank.mzML chromatograms deconvoluted deisotoped

Neg_KM-Frank.mzML#1949 @5.34 MS2 (485.3263) p -, base peak: 453.2973 m/z (7.2E3)

Scan definition: scanId=320243

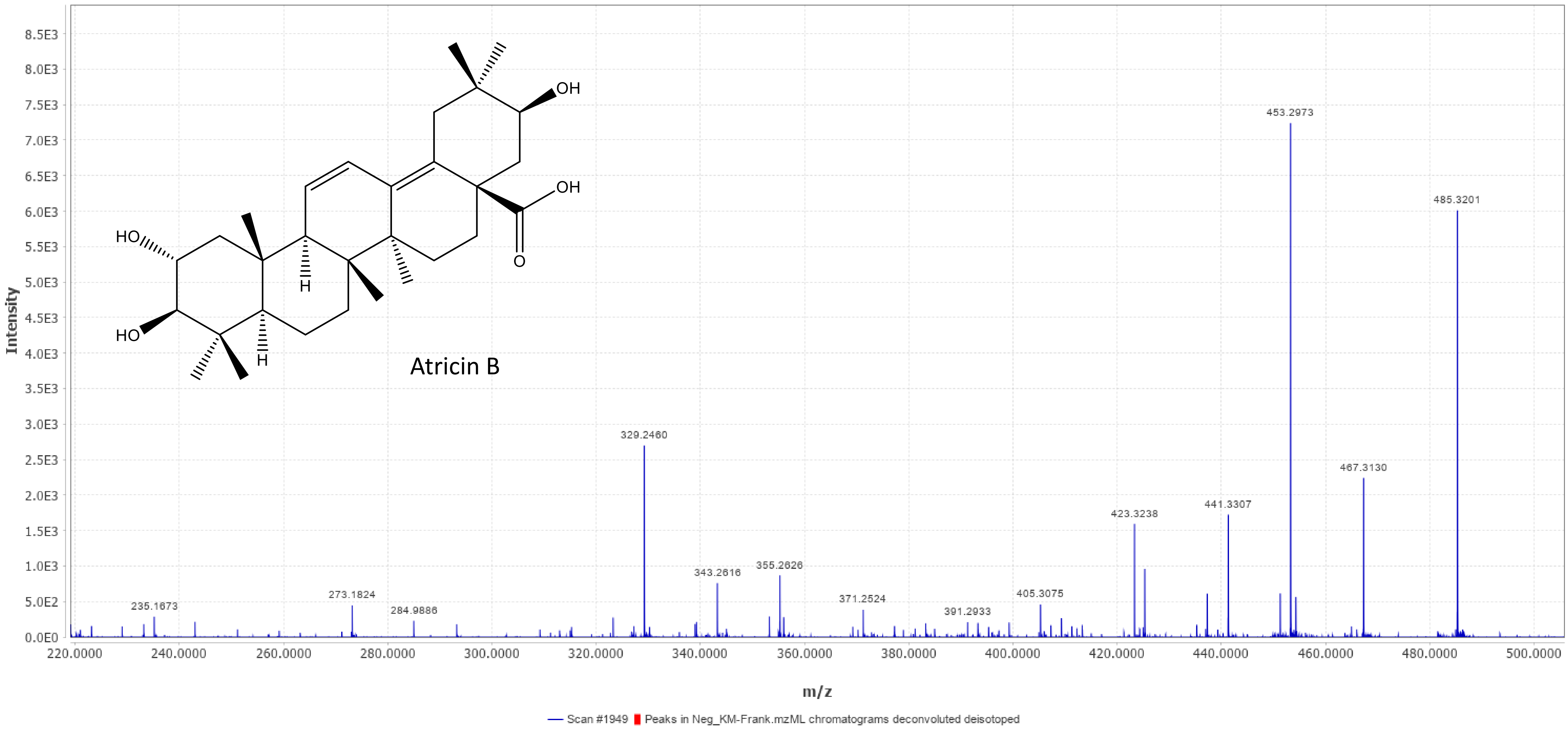

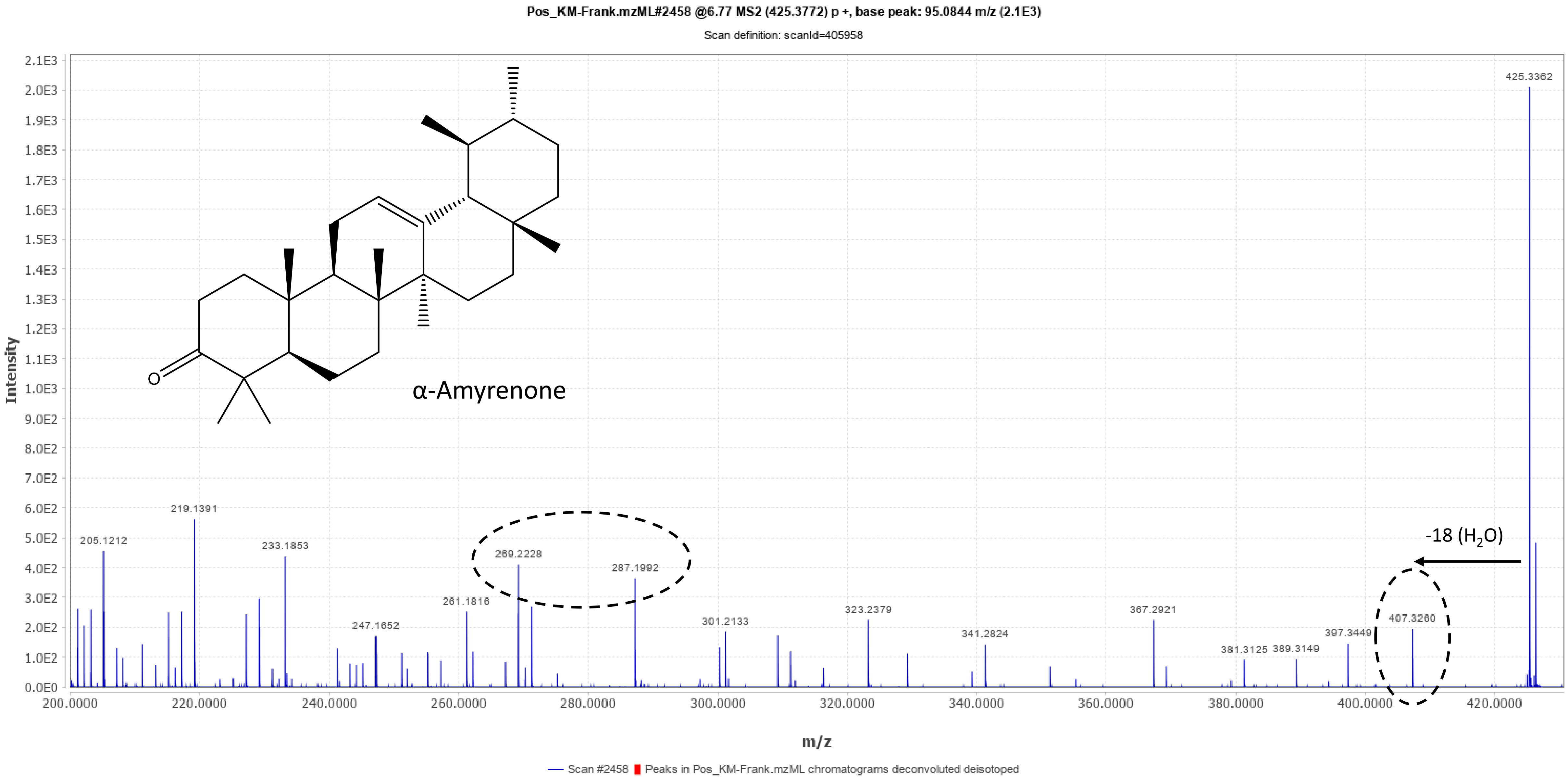
Pos_KM-Frank.mzML#2458 @6.77 MS2 (425.3772) p +, base peak: 95.0844 m/z (2.1E3)
Scan definition: scanId=405958
Intensity
m/z
α-Amyrenone
O
-18 (H_2O)
425.3362
407.3260
397.3449
389.3149
381.3125
367.2921
341.2824
323.2379
301.2133
287.1992
269.2228
261.1816
247.1652
233.1853
219.1391
205.1212
Scan #2458
Peaks in Pos_KM-Frank.mzML chromatograms deconvoluted deisotoped

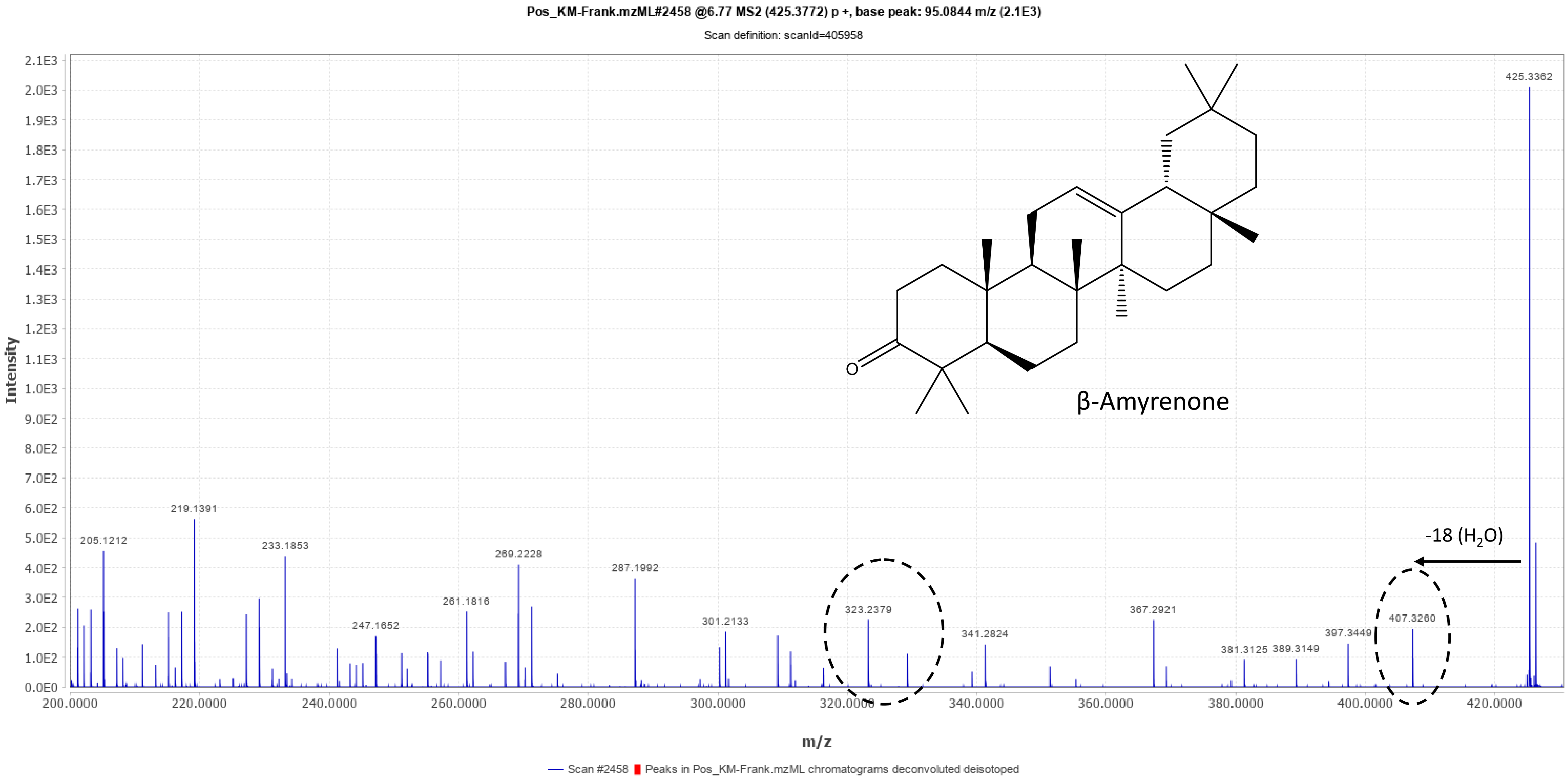

Pos_KM-Frank.mzML#2458 @6.77 MS2 (425.3772) p +, base peak: 95.0844 m/z (2.1E3)
Scan definition: scanId=405958
Intensity
m/z
205.1212
219.1391
233.1853
247.1652
261.1816
269.2228
287.1992
301.2133
323.2379
341.2824
367.2921
381.3125
389.3149
397.3449
407.3260
425.3362
-18 (H_2O)
O
β-Amyrenone
Scan #2458
Peaks in Pos_KM-Frank.mzML chromatograms deconvoluted deisotoped

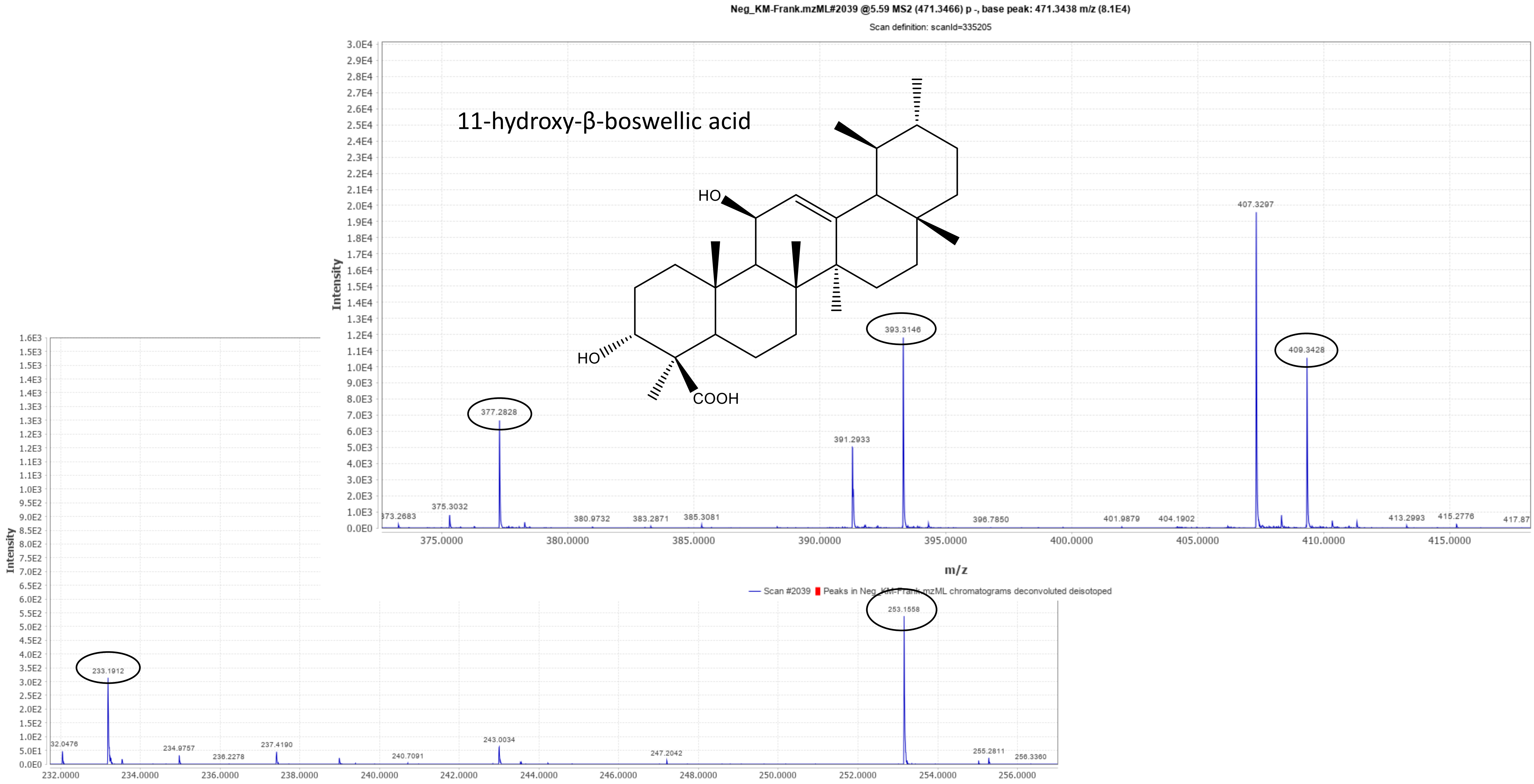
Neg_KM-Frank.mzML#2039 @5.59 MS2 (471.3466) p -, base peak: 471.3438 m/z (8.1E4)
Scan definition: scanId=335205
11-hydroxy-β-boswellic acid
HO
HO
COOH
Intensity
m/z
407.3297
409.3428
393.3146
391.2933
377.2828
375.3032
373.2683
380.9732
383.2871
385.3081
396.7850
401.9879
404.1902
413.2993
415.2776
Scan #2039
Peaks in Neg_KM-Frank.mzML chromatograms deconvoluted deisotoped
253.1558
233.1912
234.9757
236.2278
237.4190
240.7091
243.0034
247.2042
255.2811
256.3360

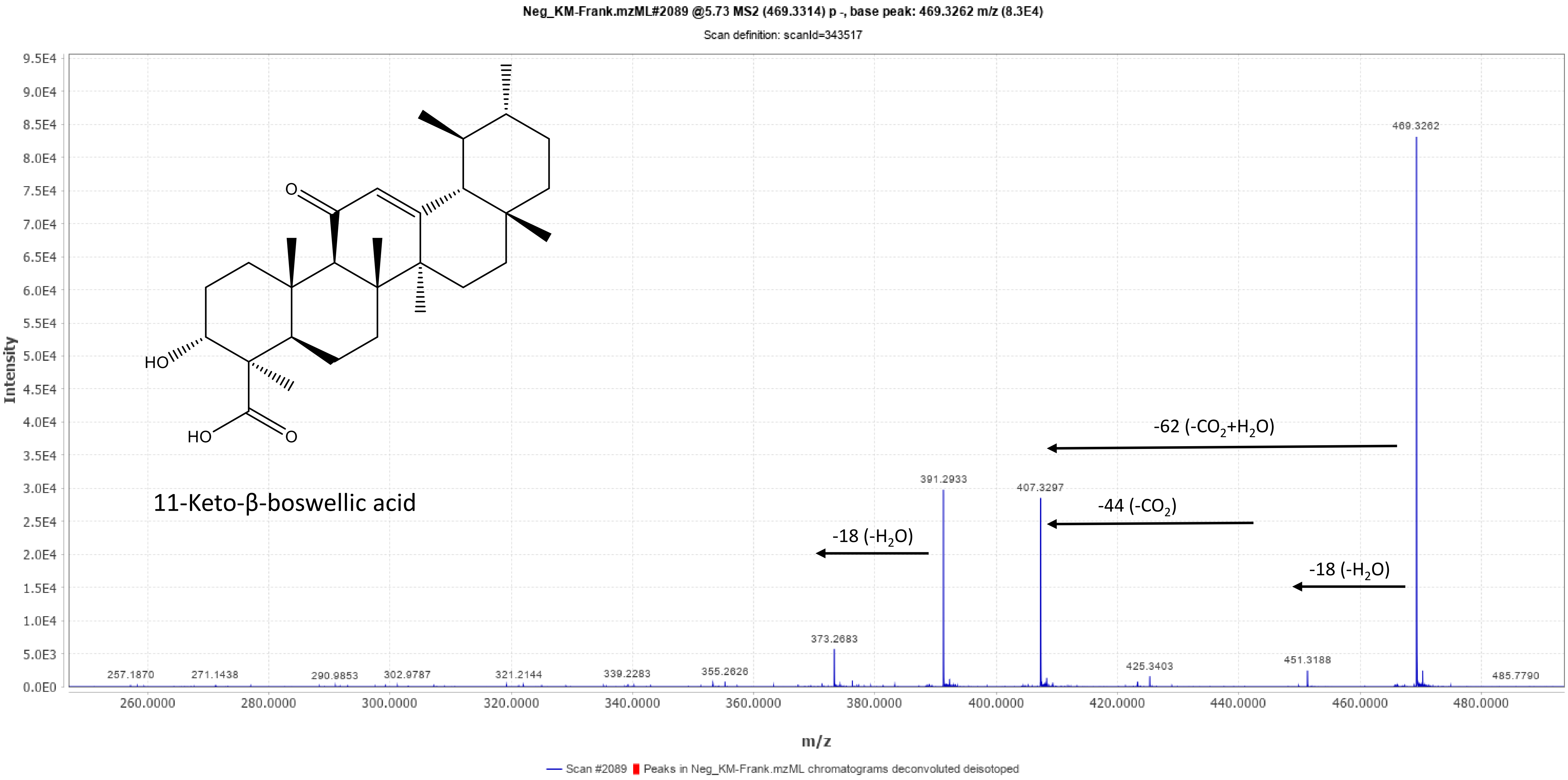

Neg_KM-Frank.mzML#2089 @5.73 MS2 (469.3314) p -, base peak: 469.3262 m/z (8.3E4)
Scan definition: scanId=343517
Intensity
m/z
11-Keto-β-boswellic acid
-62 (-CO2+H2O)
-44 (-CO2)
-18 (-H2O)
-18 (-H2O)
469.3262
407.3297
391.2933
373.2683
451.3188
425.3403
355.2626
339.2283
321.2144
302.9787
290.9853
271.1438
257.1870
485.7790
Scan #2089
Peaks in Neg_KM-Frank.mzML chromatograms deconvoluted deisotoped

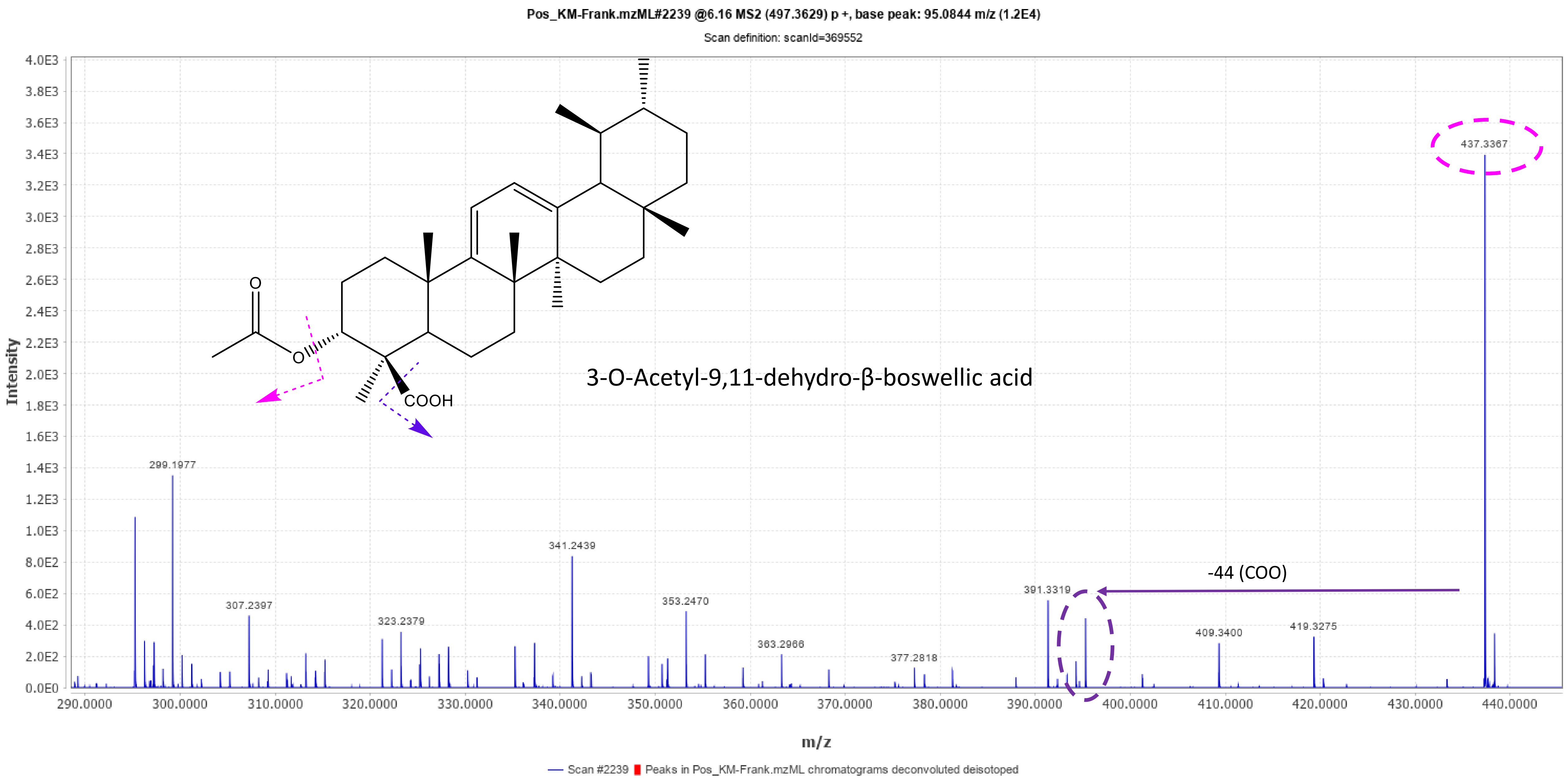

Pos_KM-Frank.mzML#2239 @6.16 MS2 (497.3629) p +, base peak: 95.0844 m/z (1.2E4)
Scan definition: scanId=369552
Intensity
m/z
3-O-Acetyl-9,11-dehydro-β-boswellic acid
COOH
-44 (COO)
437.3367
299.1977
341.2439
307.2397
353.2470
323.2379
391.3319
363.2966
409.3400
419.3275
377.2818
Scan #2239 Peaks in Pos_KM-Frank.mzML chromatograms deconvoluted deisotoped

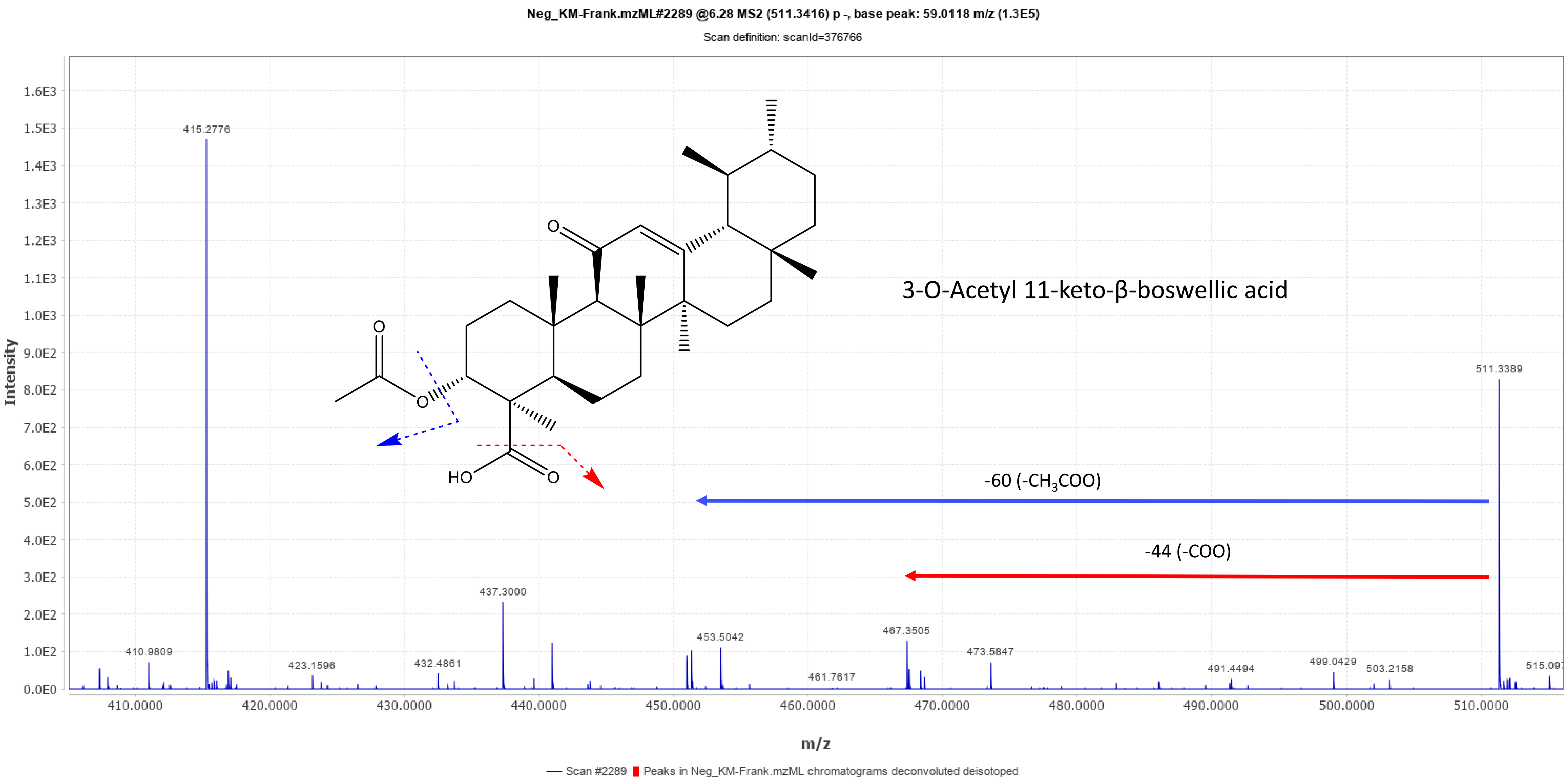
Neg_KM-Frank.mzML#2289 @6.28 MS2 (511.3416) p -, base peak: 59.0118 m/z (1.3E5)
Scan definition: scanId=376766
3-O-Acetyl 11-keto-β-boswellic acid
-60 (-CH3COO)
-44 (-COO)
415.2776
437.3000
453.5042
467.3505
473.5847
511.3389
Intensity
m/z
Scan #2289 Peaks in Neg_KM-Frank.mzML chromatograms deconvoluted deisotoped

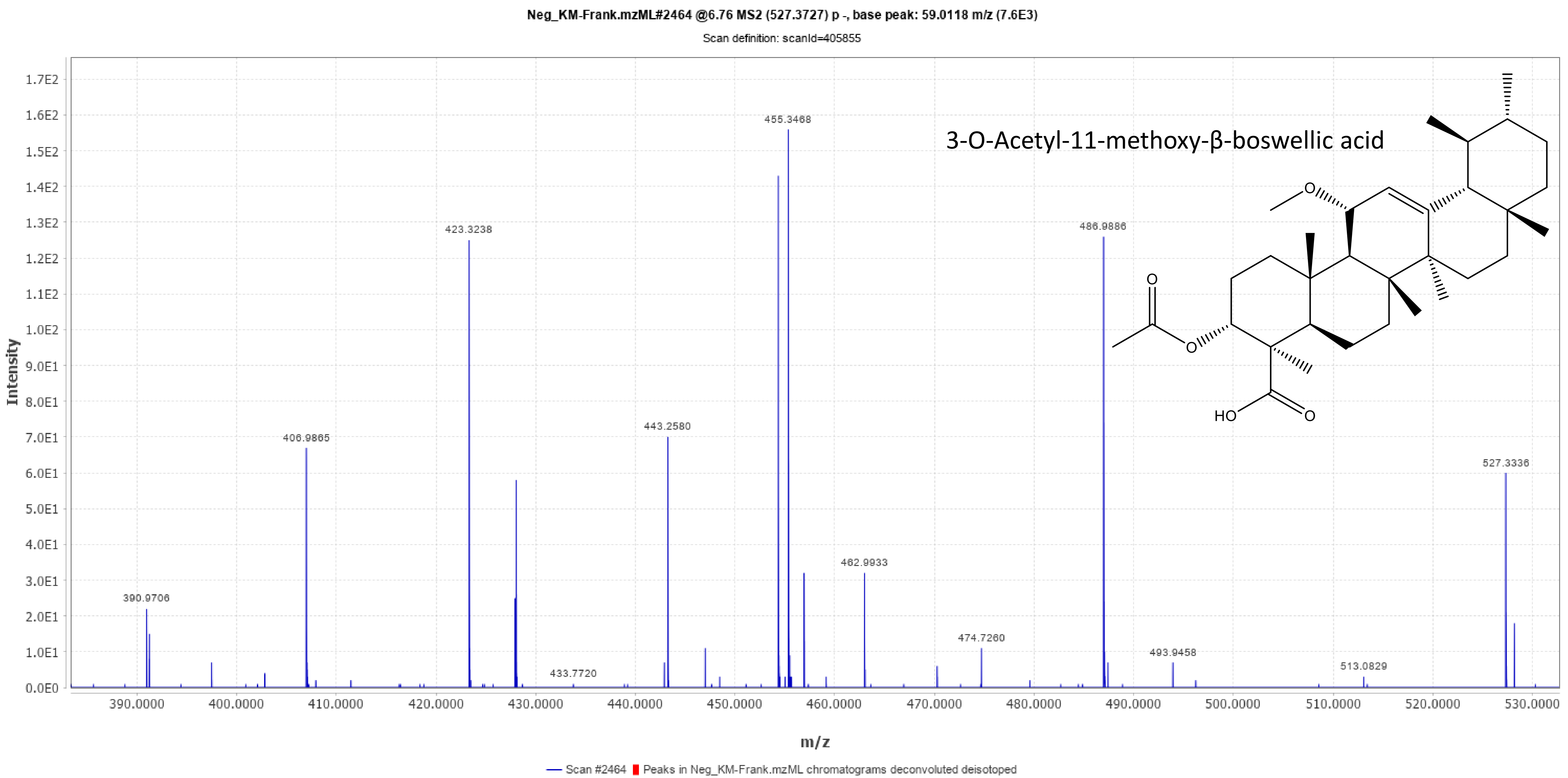

Neg_KM-Frank.mzML#2464 @6.76 MS2 (527.3727) p -, base peak: 59.0118 m/z (7.6E3)
Scan definition: scanId=405855
3-O-Acetyl-11-methoxy-β-boswellic acid
Intensity
m/z
390.9706
406.9865
423.3238
433.7720
443.2580
455.3468
462.9933
474.7260
486.9886
493.9458
513.0829
527.3336
Scan #2464
Peaks in Neg_KM-Frank.mzML chromatograms deconvoluted deisotoped

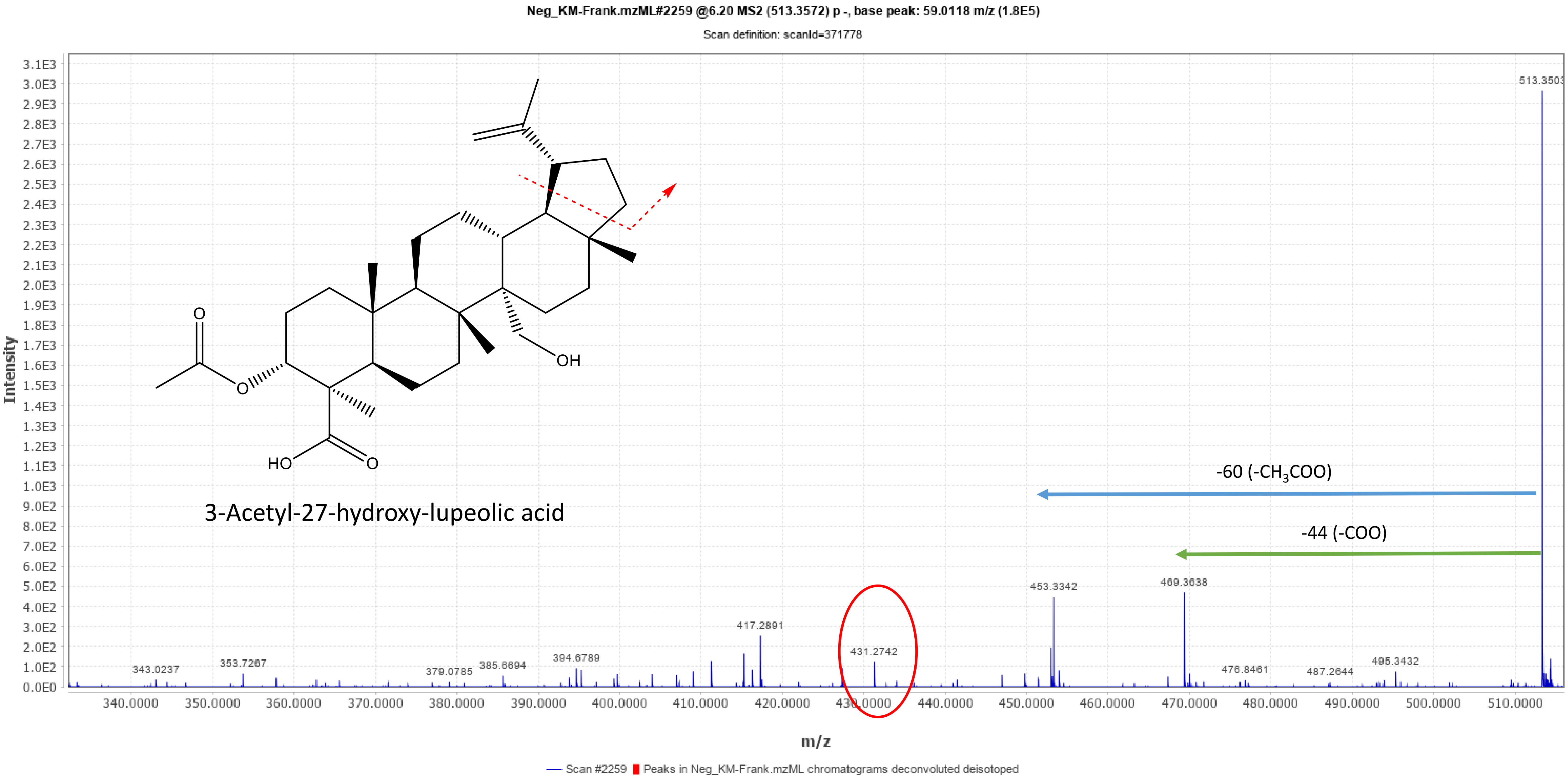
Neg_KM-Frank.mzML#2259 @6.20 MS2 (513.3572) p -, base peak: 59.0118 m/z (1.8E5)
Scan definition: scanId=371778
Intensity
m/z
513.3503
469.3638
453.3342
431.2742
417.2891
495.3432
487.2644
476.8461
394.6789
385.6694
379.0785
353.7267
343.0237
-60 (-CH3COO)
-44 (-COO)
3-Acetyl-27-hydroxy-lupeolic acid
OH
HO
O
Scan #2259
Peaks in Neg_KM-Frank.mzML chromatograms deconvoluted deisotoped

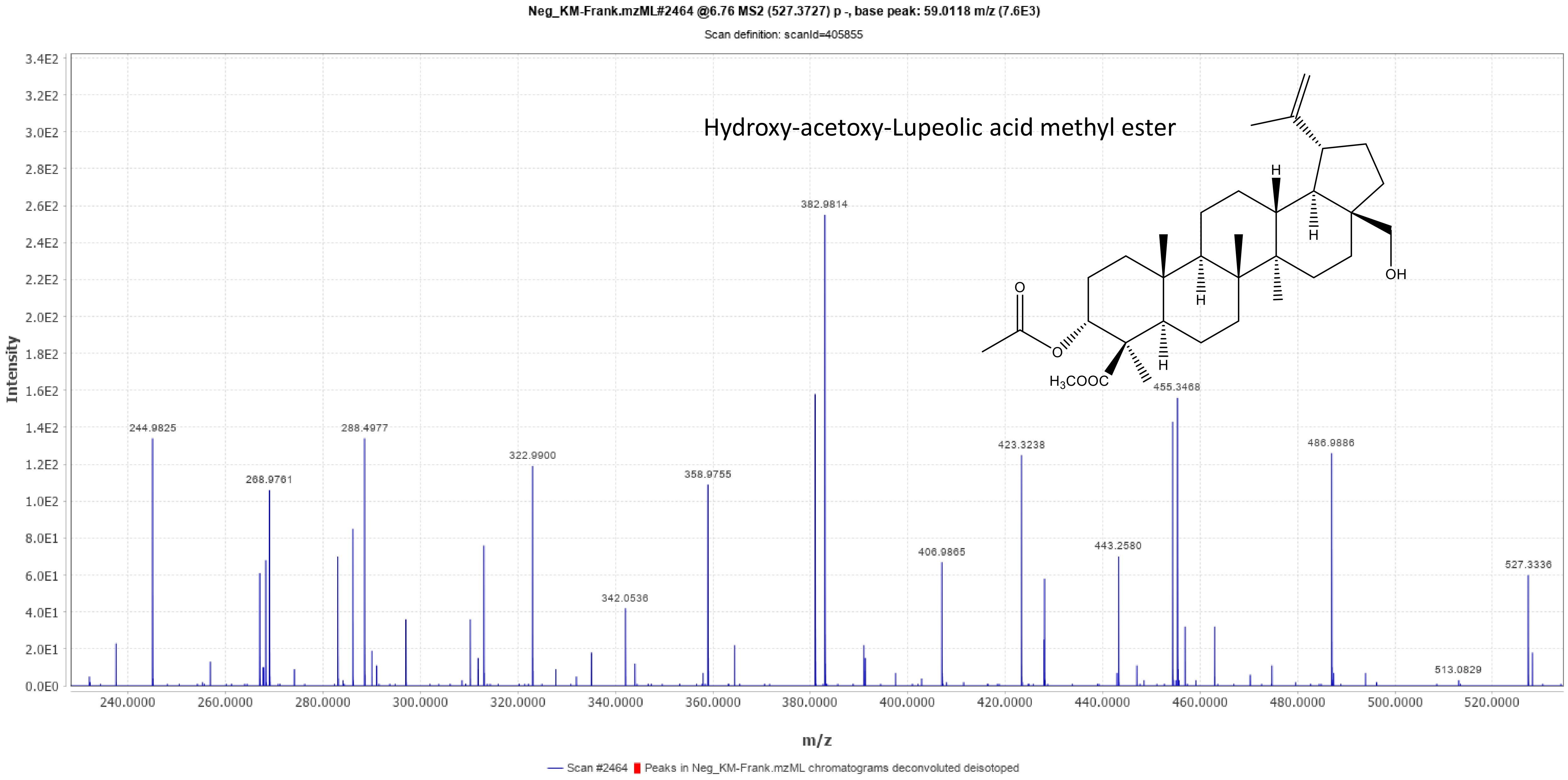

Neg_KM-Frank.mzML#2464 @6.76 MS2 (527.3727) p -, base peak: 59.0118 m/z (7.6E3)
Scan definition: scanId=405855
Hydroxy-acetoxy-Lupeolic acid methyl ester
H
H
H
H
O
O
OH
H3COOC
382.9814
244.9825
288.4977
268.9761
322.9900
358.9755
342.0536
406.9865
423.3238
443.2580
455.3468
486.9886
527.3336
513.0829
Intensity
m/z
Scan #2464
Peaks in Neg_KM-Frank.mzML chromatograms deconvoluted deisotoped

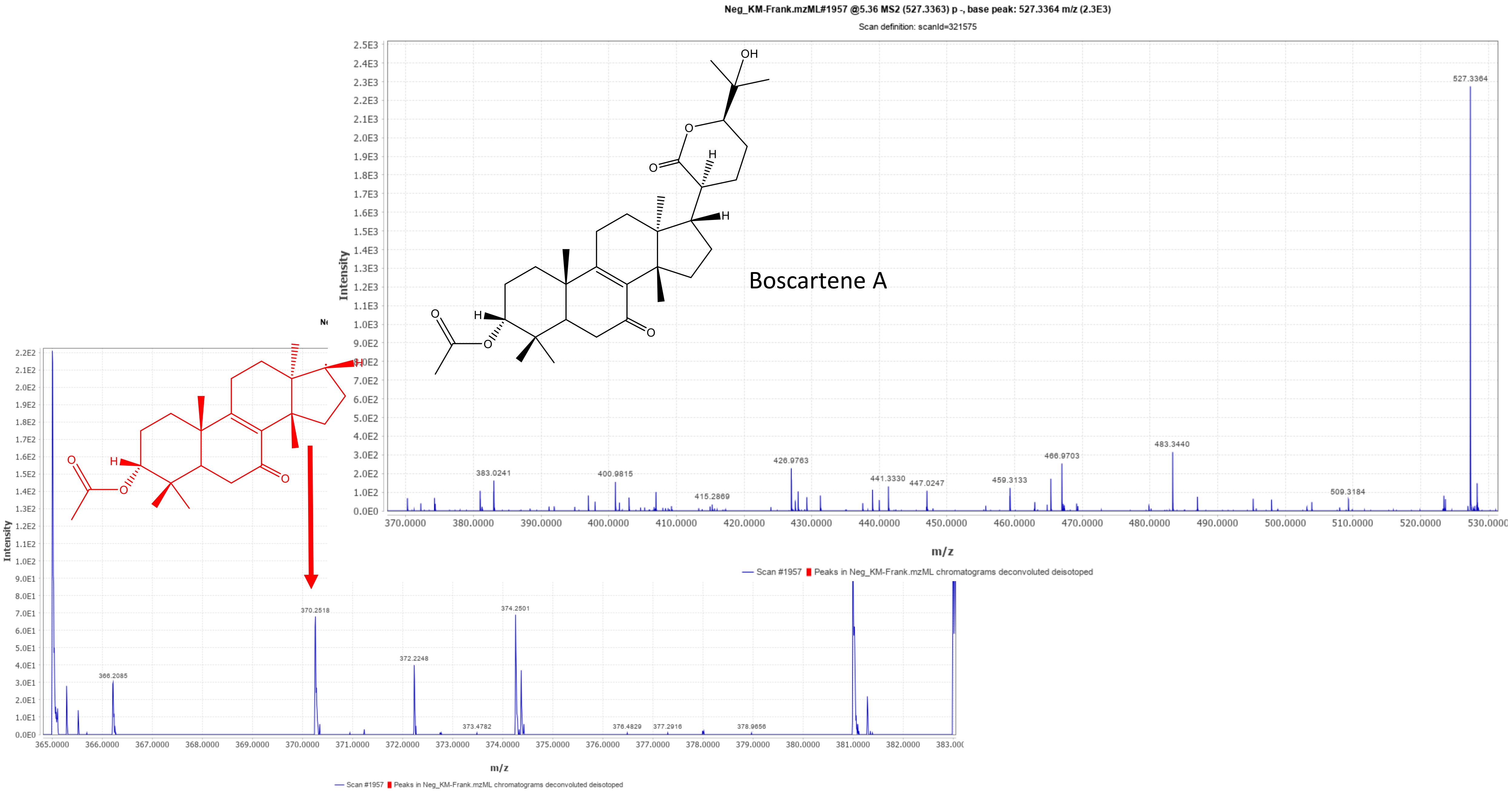

Neg_KM-Frank.mzML#1957 @5.36 MS2 (527.3363) p -, base peak: 527.3364 m/z (2.3E3)
Scan definition: scanId=321575
Intensity
m/z
527.3364
483.3440
466.9703
459.3133
447.0247
441.3330
426.9763
415.2869
400.9815
383.0241
509.3184
Boscartene A
OH
O
H
Scan #1957 Peaks in Neg_KM-Frank.mzML chromatograms deconvoluted deisotoped
366.2085
370.2518
372.2248
373.4782
374.2501
376.4829
377.2916
378.9656

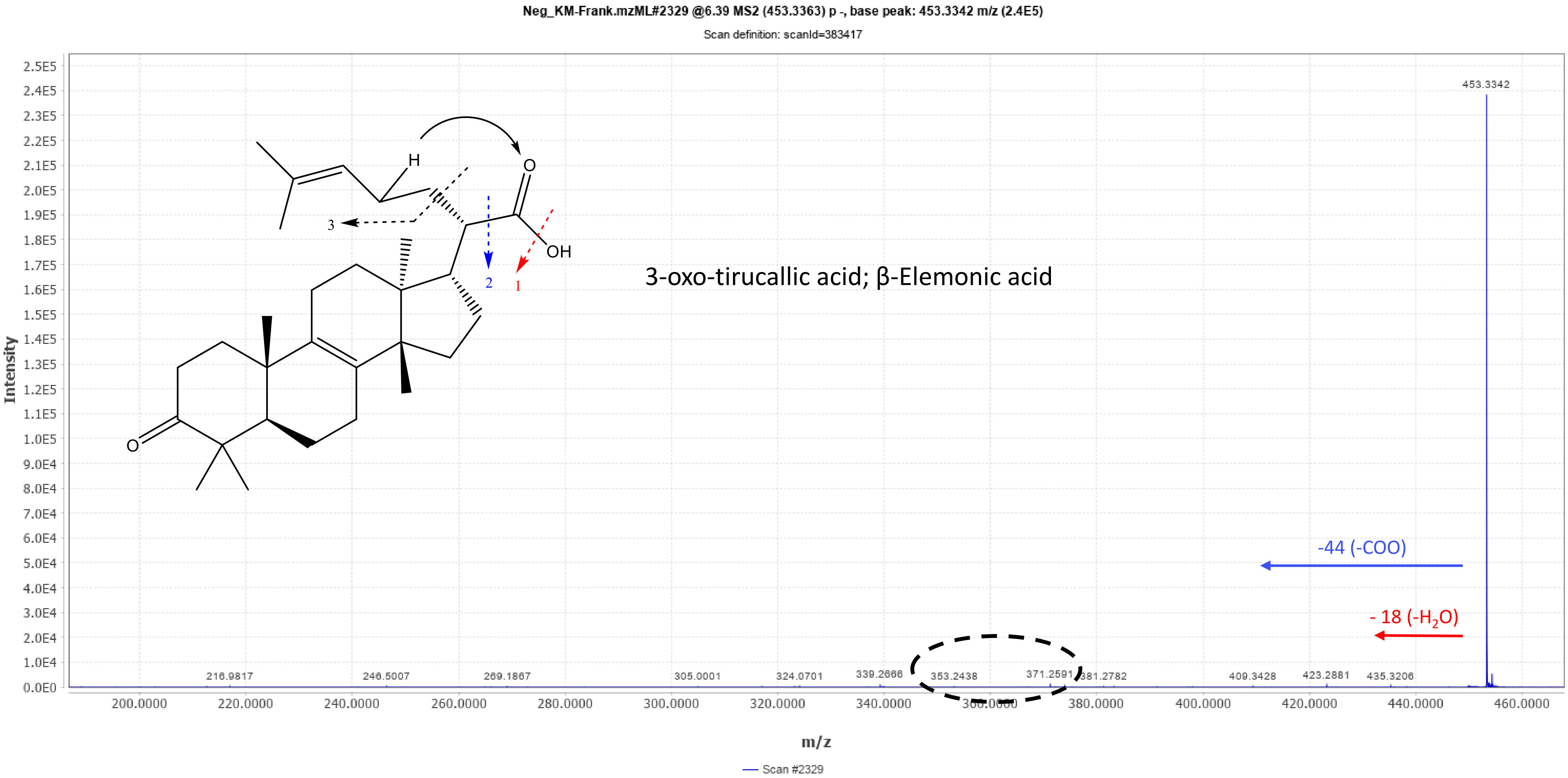

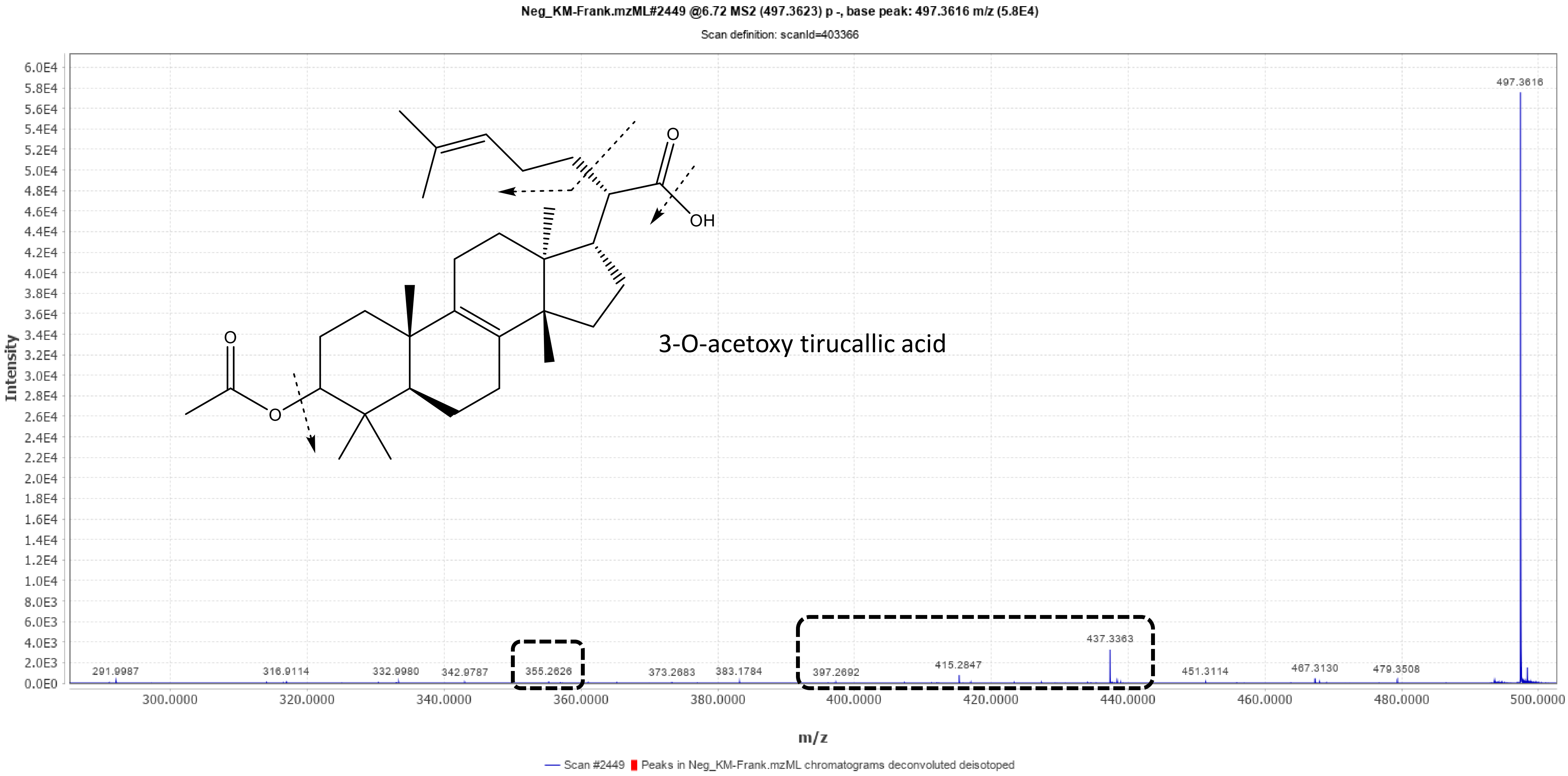

Neg_KM-Frank.mzML#2449 @6.72 MS2 (497.3623) p -, base peak: 497.3616 m/z (5.8E4)
Scan definition: scanId=403366
Intensity
m/z
3-O-acetoxy tirucallic acid
497.3616
437.3363
415.2847
397.2692
355.2626
291.9987
316.9114
332.9980
342.9787
373.2683
383.1784
451.3114
467.3130
479.3508
Scan #2449
Peaks in Neg_KM-Frank.mzML chromatograms deconvoluted deisotoped

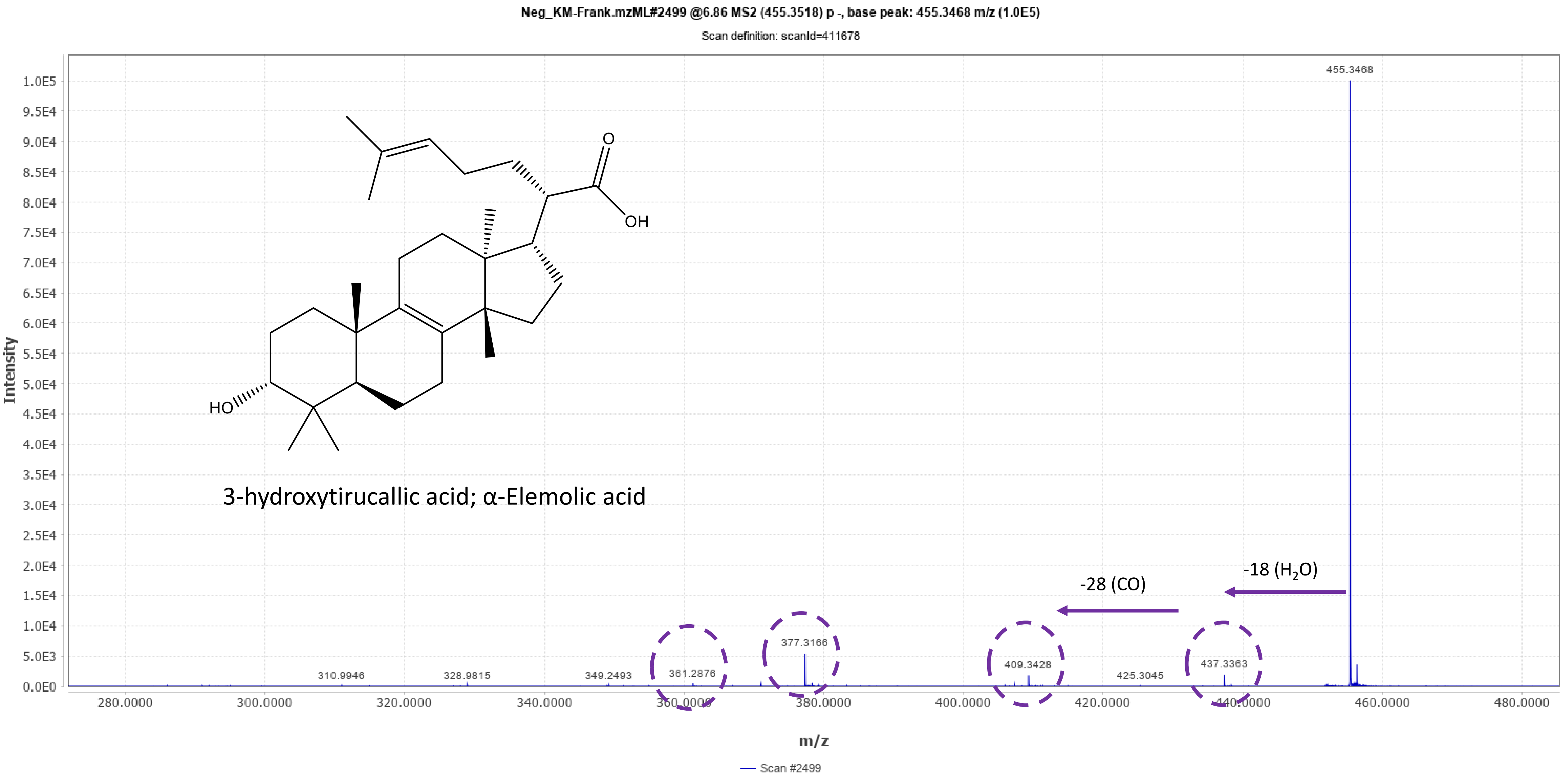

Neg_KM-Frank.mzML#2499 @6.86 MS2 (455.3518) p -, base peak: 455.3468 m/z (1.0E5)
Scan definition: scanId=411678
Intensity
m/z
Scan #2499
455.3468
437.3363
425.3045
409.3428
377.3166
361.2876
349.2493
328.9815
310.9946
-18 (H_2O)
-28 (CO)
O
OH
HO
3-hydroxytirucallic acid; α-Elemolic acid

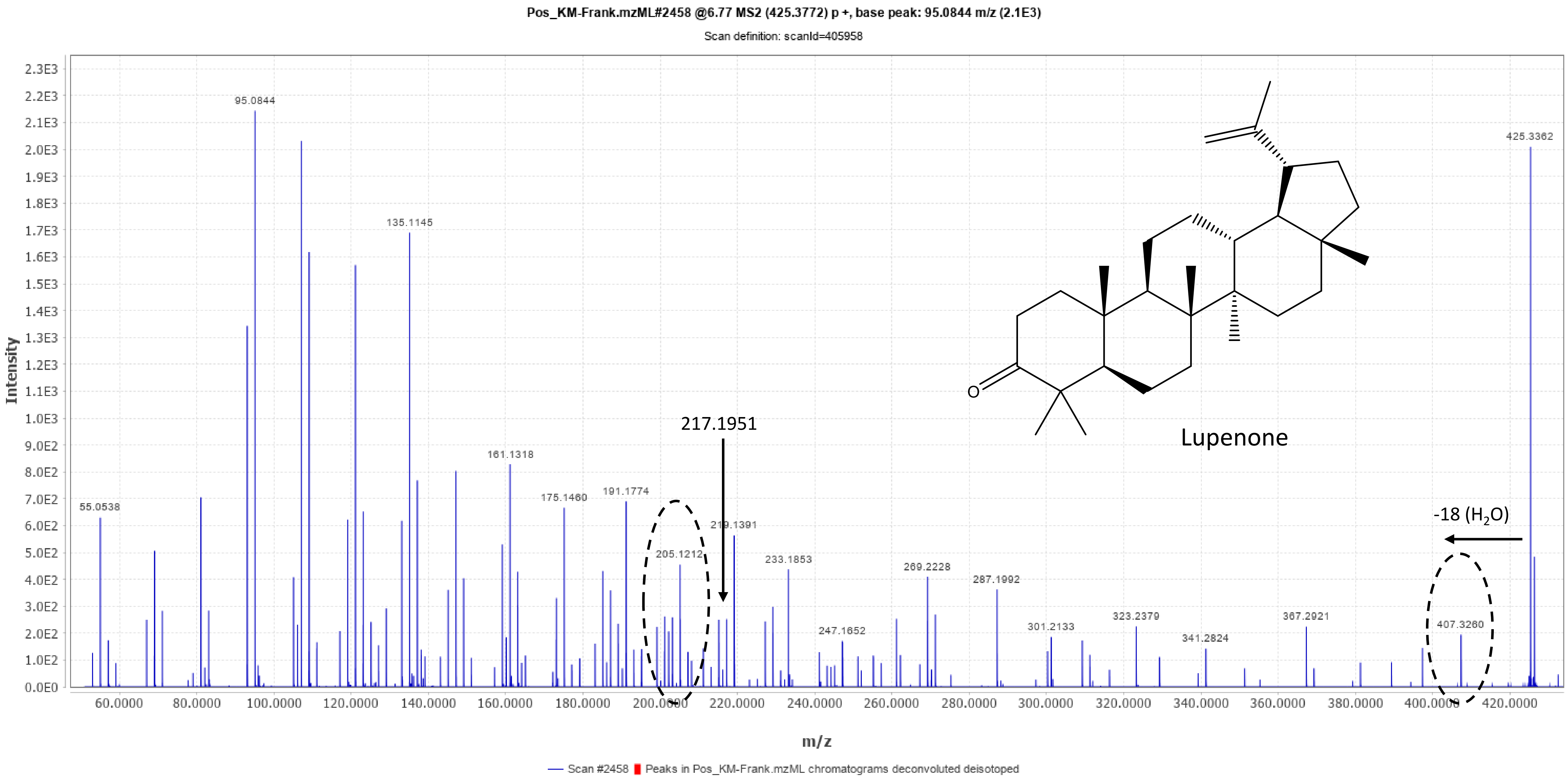
Pos_KM-Frank.mzML#2458 @6.77 MS2 (425.3772) p +, base peak: 95.0844 m/z (2.1E3)
Scan definition: scanId=405958
Intensity
m/z
95.0844
135.1145
161.1318
175.1460
191.1774
55.0538
205.1212
219.1391
217.1951
233.1853
247.1652
269.2228
287.1992
301.2133
323.2379
341.2824
367.2921
407.3260
425.3362
-18 (H_2O)
O
Lupenone
Scan #2458
Peaks in Pos_KM-Frank.mzML chromatograms deconvoluted deisotoped

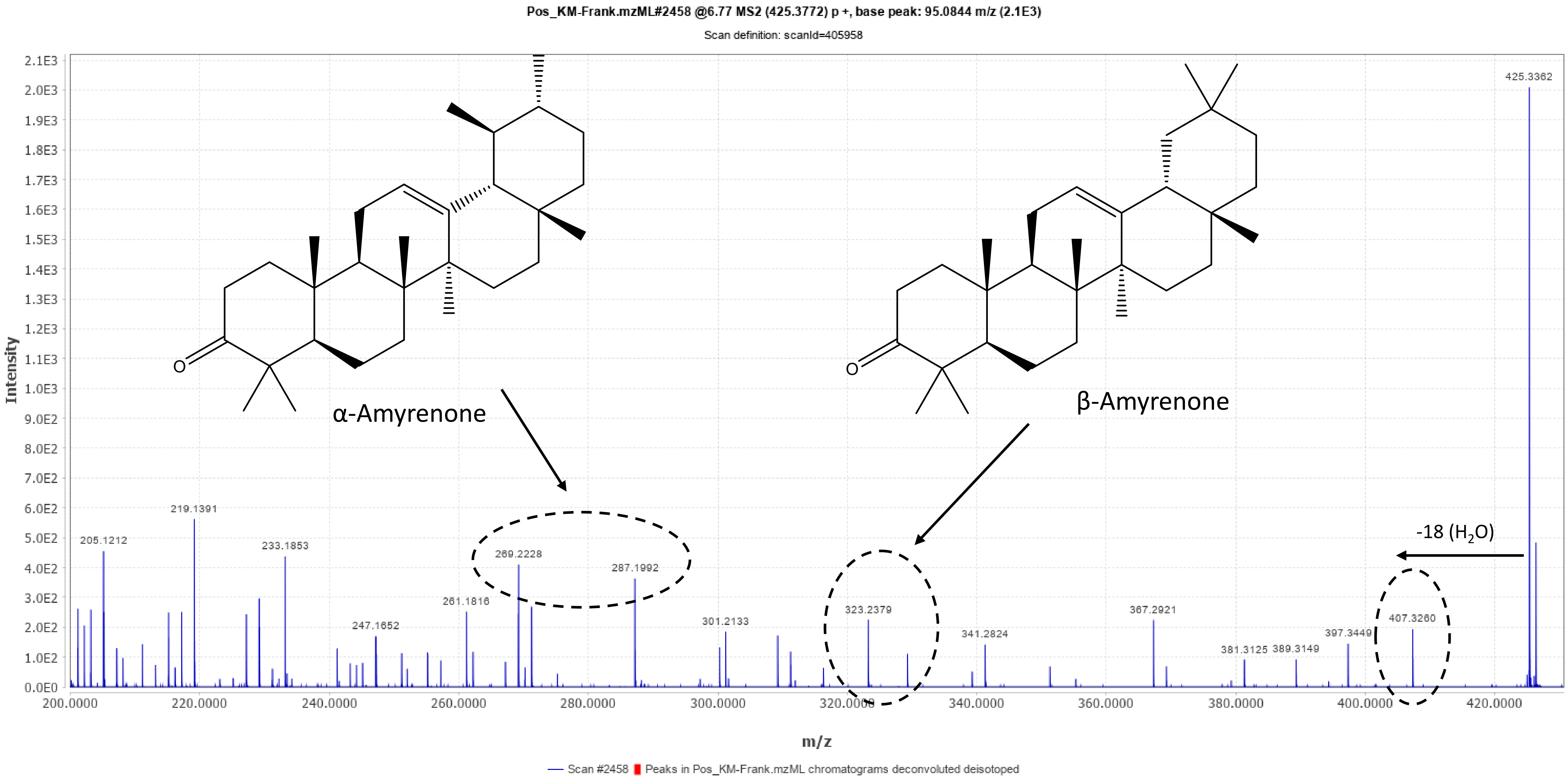

Pos_KM-Frank.mzML#2458 @6.77 MS2 (425.3772) p +, base peak: 95.0844 m/z (2.1E3)
Scan definition: scanId=405958
Intensity
m/z
α-Amyrenone
β-Amyrenone
-18 (H2O)
425.3362
407.3260
397.3449
389.3149
381.3125
367.2921
341.2824
323.2379
301.2133
287.1992
269.2228
261.1816
247.1652
233.1853
219.1391
205.1212
Scan #2458
Peaks in Pos_KM-Frank.mzML chromatograms deconvoluted deisotoped
